# Genetic analysis of *ATP7B* in 102 south Indian families with Wilson disease

**DOI:** 10.1371/journal.pone.0215779

**Published:** 2019-05-06

**Authors:** Nivedita Singh, Pradeep Kallollimath, Mohd Hussain Shah, Saketh Kapoor, Vishwanath Kumble Bhat, Lakshminarayanapuram Gopal Viswanathan, Madhu Nagappa, Parayil S. Bindu, Arun B. Taly, Sanjib Sinha, Arun Kumar

**Affiliations:** 1 Department of Molecular Reproduction, Development and Genetics, Indian Institute of Science, Bangalore, India; 2 Department of Neurology, National Institute of Mental Health and Neuro Sciences, Bangalore, India; Medizinische Universitat Innsbruck Department fur Kinder- und Jugendheilkunde, AUSTRIA

## Abstract

Wilson disease (WD) is an autosomal recessive disorder, characterized by excessive deposition of copper in various parts of the body, mainly in the liver and brain. It is caused by mutations in *ATP7B*. We report here the genetic analysis of 102 WD families from a south Indian population. Thirty-six different *ATP7B* mutations, including 13 novel ones [p.Ala58fs*19, p.Lys74fs*9, p.Gln281*, p.Pro350fs*12, p.Ser481*, p.Leu735Arg, p.Val752Gly, p.Asn812fs*2, p.Val845Ala, p.His889Pro, p.Ile1184fs*1, p.Val1307Glu and p.Ala1339Pro], were identified in 76/102 families. Interestingly, the mutation analysis of affected individuals in two families identified two different homozygous mutations in each family, and thus each affected individual from these families harbored two mutations in each *ATP7B* allele. Of 36 mutations, 28 were missense, thus making them the most prevalent mutations identified in the present study. Nonsense, insertion and deletion represented 3/36, 2/36 and 3/36 mutations, respectively. The haplotype analysis suggested founder effects for all the 14 recurrent mutations. Our study thus expands the mutational landscape of *ATP7B* with a total number of 758 mutations. The mutations identified during the present study will facilitate carrier and pre-symptomatic detection, and prenatal genetic diagnosis in affected families.

## Introduction

Wilson disease (WD, MIM #277900) is an autosomal recessive disorder, characterized by the excessive deposition of copper in the body, mainly in the liver and brain. The worldwide disease incidence of WD is 1/5,000–1/30,000 live births. The disease presentation varies from as early as 2 years to as old as 72 years of age [1–3]. WD patients commonly manifest with hepatic and neuropsychiatric problems. The hepatic manifestations are acute hepatitis, chronic active hepatitis, cirrhosis and acute fulminant hepatic failure. Patients with neuropsychiatric manifestations have dysarthria, dystonia, tremor, ataxia, parkinsonian features, behavioral problems and cognitive disturbances. *ATP7B* (MIM #606882; ATPase copper transporting beta), the causative gene of WD, is located on the chromosome 13q14.3-q21 [4–6]. It has 21 coding exons and codes for a 1,465 amino acid long protein of 165 kDa, which contains following domains: six copper binding domains (CBD1-6), eight transmembrane domains (TMS1-8), A-domain and ATP binding domain. It shows granular cytoplasmic expression in most tissues (https://www.proteinatlas.org/ENSG00000123191-ATP7B/tissue), and resides mainly in the trans-Golgi network (TGN). Under normal physiological conditions, ATP7B delivers copper to apoceruloplasmin. As the copper level increases inside the cells, ATP7B traffics to the vesicular compartments and lysosomes to remove excess of copper into the bile [7].

Currently, the diagnosis of WD is based mainly on a combination of different clinical features (e.g., the presence of corneal Kayser-Fleischer ring, hepatic and neurological abnormalities) and biochemical tests such as serum ceruloplasmin concentration, 24 hours urinary copper excretion, hepatic copper determination, and serum copper levels. However, although the first case of WD was reported in 1912, even after so many years of the disease identification, its diagnosis often remains a challenge. Wrong and delayed diagnosis of WD patients is not uncommon and might affect their outcome. The treatment of WD at present is performed mainly by administration of chelating agents (eg., penicillamine, trientine and ammonium tetrathiomolybdate) or zinc salts (eg, zinc sulphate or zinc acetate) that prevent absorption of copper in the body. The accurate diagnosis of WD could be enhanced by genetic testing. Therefore, there is a need to perform a genetic study of WD families for carrier and pre-symptomatic detection, and genotype-phenotype correlation. Several mutations in *ATP7B* have been reported from different countries, including India (http://www.wilsondisease.med.ualberta.ca/database.asp). However, only a few large cohort studies have been documented so far from India [8–10]. In this study, we have screened the entire coding region of *ATP7B* in a large cohort of 102 families from a south Indian population, mainly from the state of Karnataka, and identified 36 different mutations in the gene.

## Materials and methods

### Families

A total of 102 WD families (Figure A in S1 File) were recruited in the Wilson’s disease clinic of the Department of Neurology, National Institute of Mental Health and Neuro Sciences (NIMHANS), Bangalore, Karnataka. The diagnosis was based on the presence of typical clinical features, Kayser-Fleischer ring by slit lamp and biochemical tests (viz., low serum copper, low serum ceruloplasmin and elevated 24 hours urinary copper), which were accompanied by brain MRI and other tests wherever required (Table A in S1 File). The informed written consent for research was obtained from individuals following the approval of the Institutional Ethics Committee of the National Institute of Mental Health and Neuro Sciences (NIMHANS), Bangalore. All methods were performed in accordance with the relevant guidelines and regulations.

### Genetic analysis

For genetic analysis, 3–5 ml of peripheral blood was drawn from each individual in a Vacutainer^TM^ EDTA tube (Becton Dickinson, Franklin Lakes, NJ) for genomic DNA isolation, using a Wizard^TM^ genomic DNA extraction kit (Promega, Madison, WI). Genomic DNA was isolated from a total of 314 individuals, including 113 affected individuals. To determine if the cause of WD in these families is due to *ATP7B* gene (GenBank accession # NM_000053.3) mutation(s), its entire coding region, including the intron-exon boundaries, was amplified using specific primers (Table B in S1 File). The 5’and 3’UTRs of the *ATP7B* gene were screened in patients without mutations in the coding region using specific primers (Table C in S1 File). For the mutation identification, PCR products were Sanger sequenced from one affected individual from each family on an ABIprism A310-automated sequencer (Life Technologies, Carlsbad, CA). Once the mutation was identified, the remaining family members were also sequenced for the presence or absence of the same mutation. To rule out the possibility that the novel variants are also present in the general population, single-strand conformation polymorphism (SSCP) and allele-specific PCR (ASP) (Table D in S1 File) were performed. The ClustalW2 program (http://www.ebi.ac.uk/Tools/msa/clustalw2/) was used for multiple sequence alignment to see the conservation of an amino acid residue affected due to a missense mutation. To determine if novel missense variants identified in the study are mutations, different bioinformatics tools (e.g., SIFT, PolyPhen-2 and Mutation Taster) were used. Other databases such as the 1000 Genomes (http://www.1000genomes.org/) and the ExAC (Exome Aggregation Consortium; http://exac.broadinstitute.org/) were used to confirm if the variants identified in this study are novel. The allele frequency of a mutation was calculated as the number of mutant alleles present in affected individuals/total number of alleles in the affected individuals.

### Genotype-phenotype correlation

For the genotype-phenotype correlation, the mutations were divided into two groups: missense and nonsense/insertion/deletion. The mutations present in only homozygous and compound heterozygous states were included in the correlation study. The phenotypes that were included for the study were as follows: age of onset, serum copper, serum ceruloplasmin, 24 hr urinary copper, Kayser-Fleischer ring, dysphagia, dysarthria, tremor, dystonia, writing difficulty, chorea, athetosis, parkinsonism, rigidity, bradykinesia, cerebral atrophy, cerebellar atrophy, brain stem atrophy, giant panda sign, hepatomegaly, splenomegaly, and jaundice. For the phenotypes which are based on the presence or absence of the symptoms, the analysis was performed by assigning ‘+’ for the presence of symptom and ‘-‘ for the absence of symptoms. The statistical significance was assessed by two-tailed unpaired t-test with Welch’s correction using the GraphPad PRISM5 software (GraphPad Software Inc., San Diego, CA, USA).

### Haplotype analysis

To establish founder effects for recurrent mutations, the haplotype analysis was performed, using microsatellite markers flanking the *ATP7B* gene (Table E in S1 File) and intragenic SNPs. The marker genotyping was carried out as described by Kumar et al. [11].

## Results and discussion

**Fig 1:**
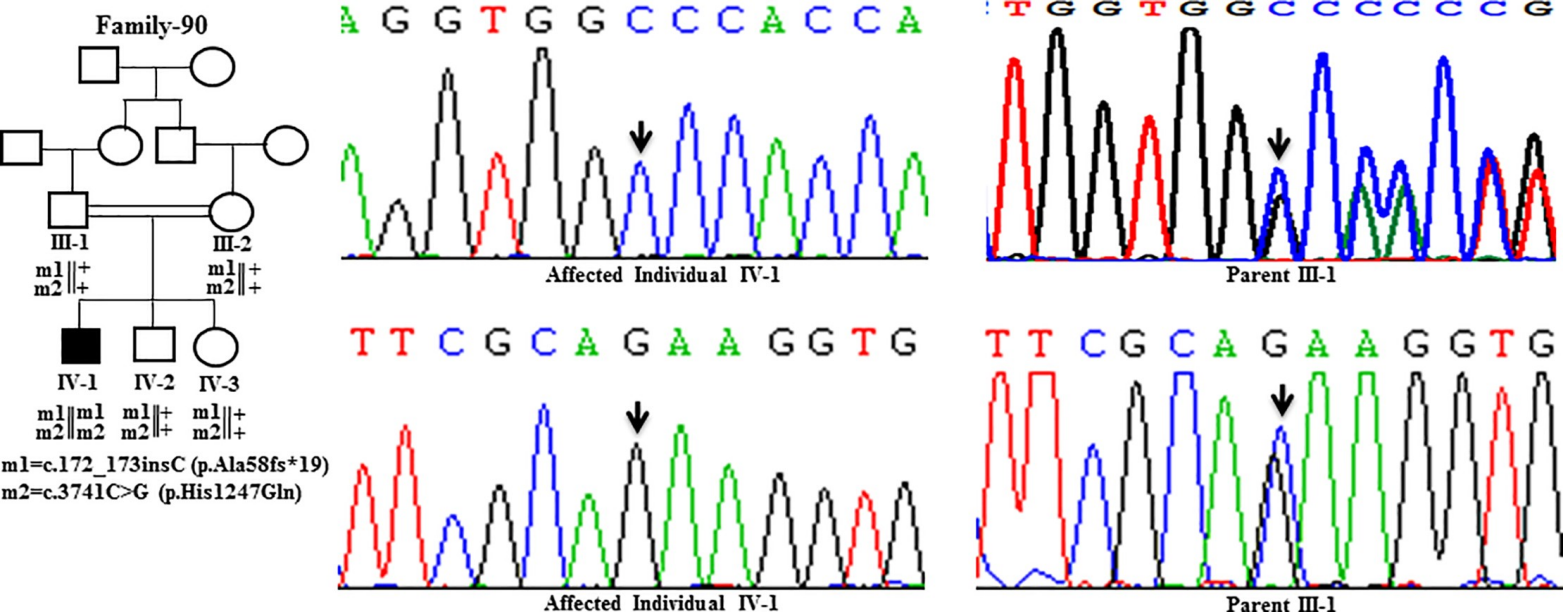
DNA sequence analysis of individuals from family-90. (Upper panel) Sequencing chromatograms from the affected individual IV-1 and the parent III-1 showing c.172_173insC mutation. Arrows mark the insertion of C in a homozygous state in the affected individual IV-1 and in a heterozygous state in the parent III-1. (Lower panel) Sequencing chromatograms from the affected individual IV-1 and the parent III-1 showing c.3741C>G mutation. Arrows mark the C>G change in a homozygous state in the affected individual IV-1 and in a heterozygous state in the parent III-1. + denotes the wild-type allele. m1 and m2 denote different mutations.

**Fig 2:**
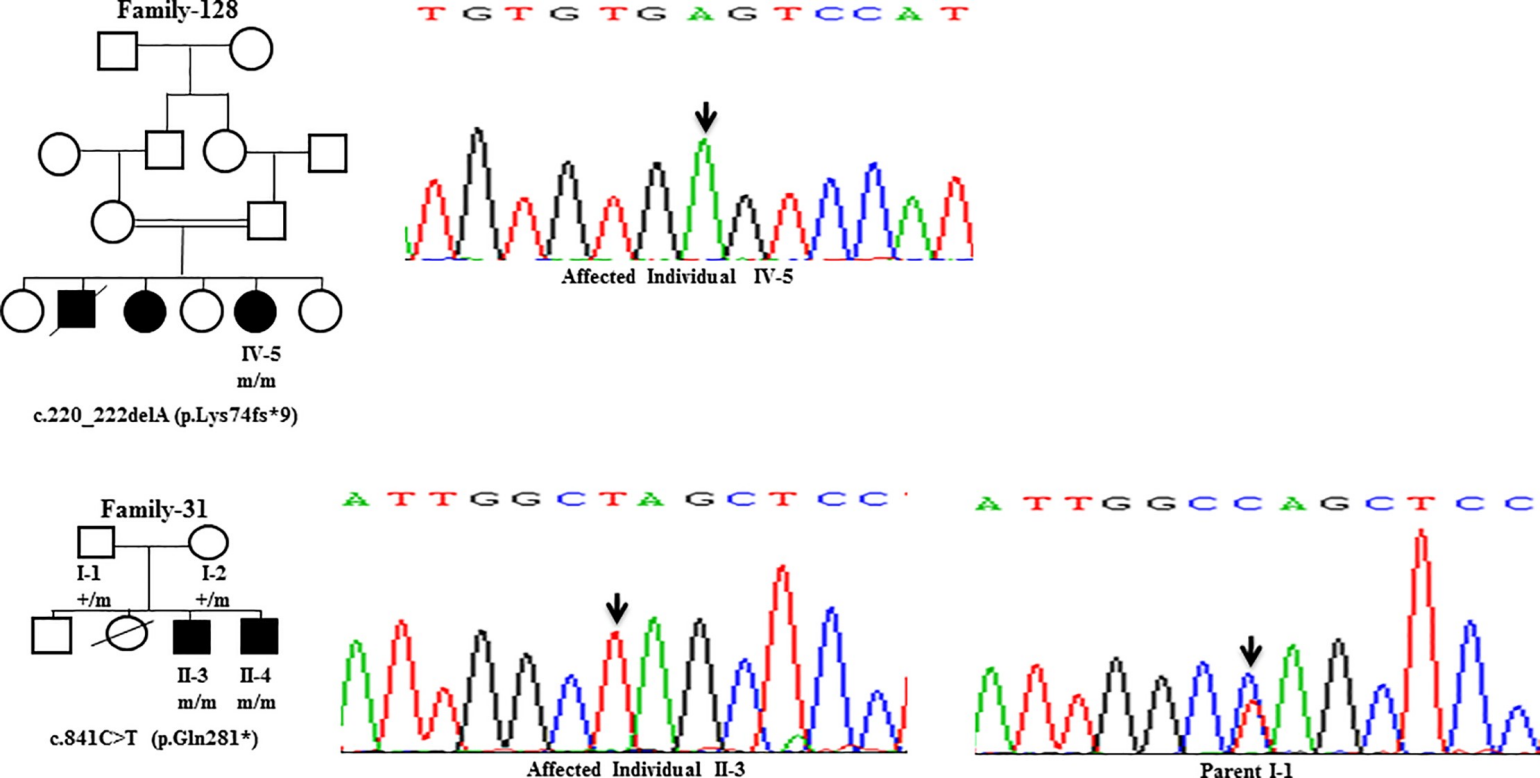
DNA sequence analysis of individuals from family-128 and family-31. (Upper panel) Sequencing chromatogram of the affected individual IV-5 from family-128. Arrow marks the deletion of A residue in a homozygous state. (Lower panel) Sequencing chromatograms of the affected individual II-3 and the parent I-1 from family-31. Arrows mark the C>T change in a homozygous state in the affected individual II-3 and in a heterozygous state in the parent I-1. + and m denote the wild-type and mutant alleles, respectively.

**Fig 3:**
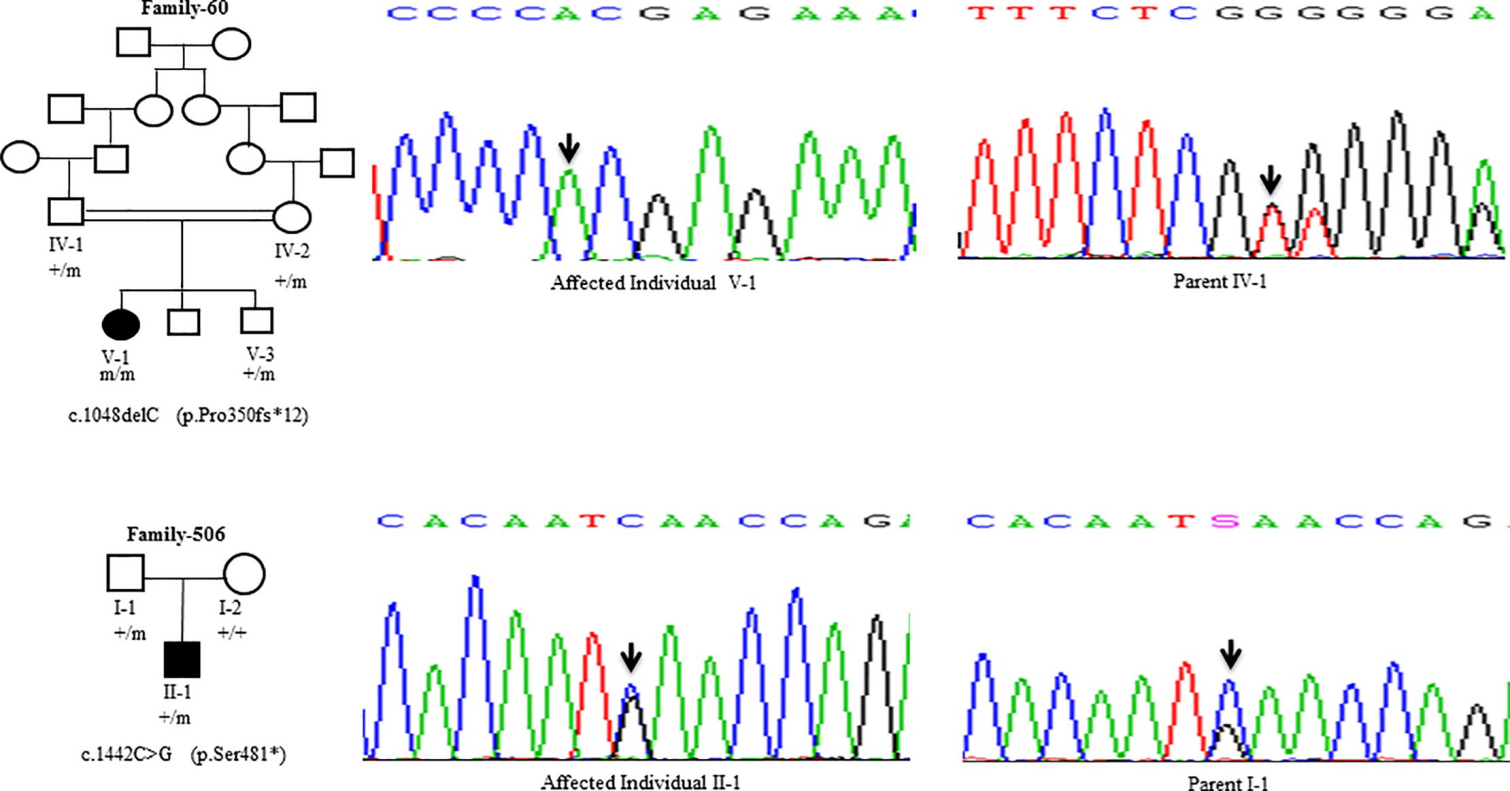
DNA sequence analysis of individuals from family-60 and family-506. (Upper panel) Sequencing chromatograms from the affected individual V-1 and parent IV-1 from family-60. Arrows mark the deletion of the C residue in a homozygous state in the affected individual V-1 and in a heterozygous state in the parent IV-1. (Lower panel) Sequencing chromatograms from the affected individual II-1 and parent I-1 from family-506. Arrows mark the C>G change in a heterozygous state in the affected individual II-1 and parent I-1. + and m denote the wild type and mutant alleles, respectively.

**Fig 4:**
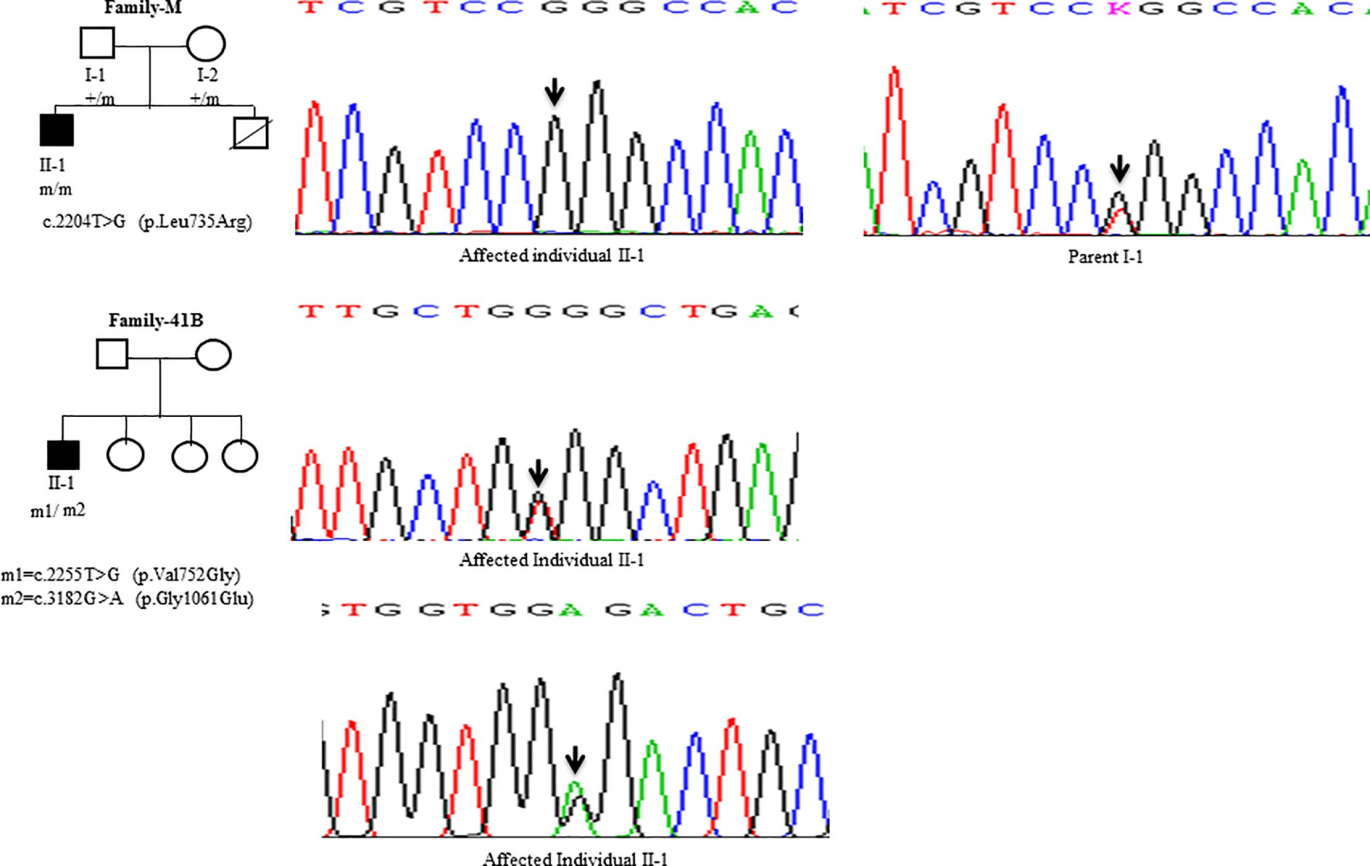
DNA sequence analysis of individuals from family-M and family-41B. (Upper panel) Sequencing chromatograms from the affected individual II-1 and parent I-1 from the family-M. Arrows mark the T>G change in a homozygous state in the affected individual II-1 and in a heterozygous state in the parent I-1. (Middle panel) Sequencing chromatogram from the affected individual II-1 from family-41B. Arrow marks the T>G change in a heterozygous state in the affected individual II-1. (Lower panel) Sequencing chromatogram from the affected individual II-1 from family-41B. Arrow marks the G>A change in a heterozygous state in the affected individual II-1. + denotes the wild type allele. m1 and m2 denote two different mutations.

**Fig 5:**
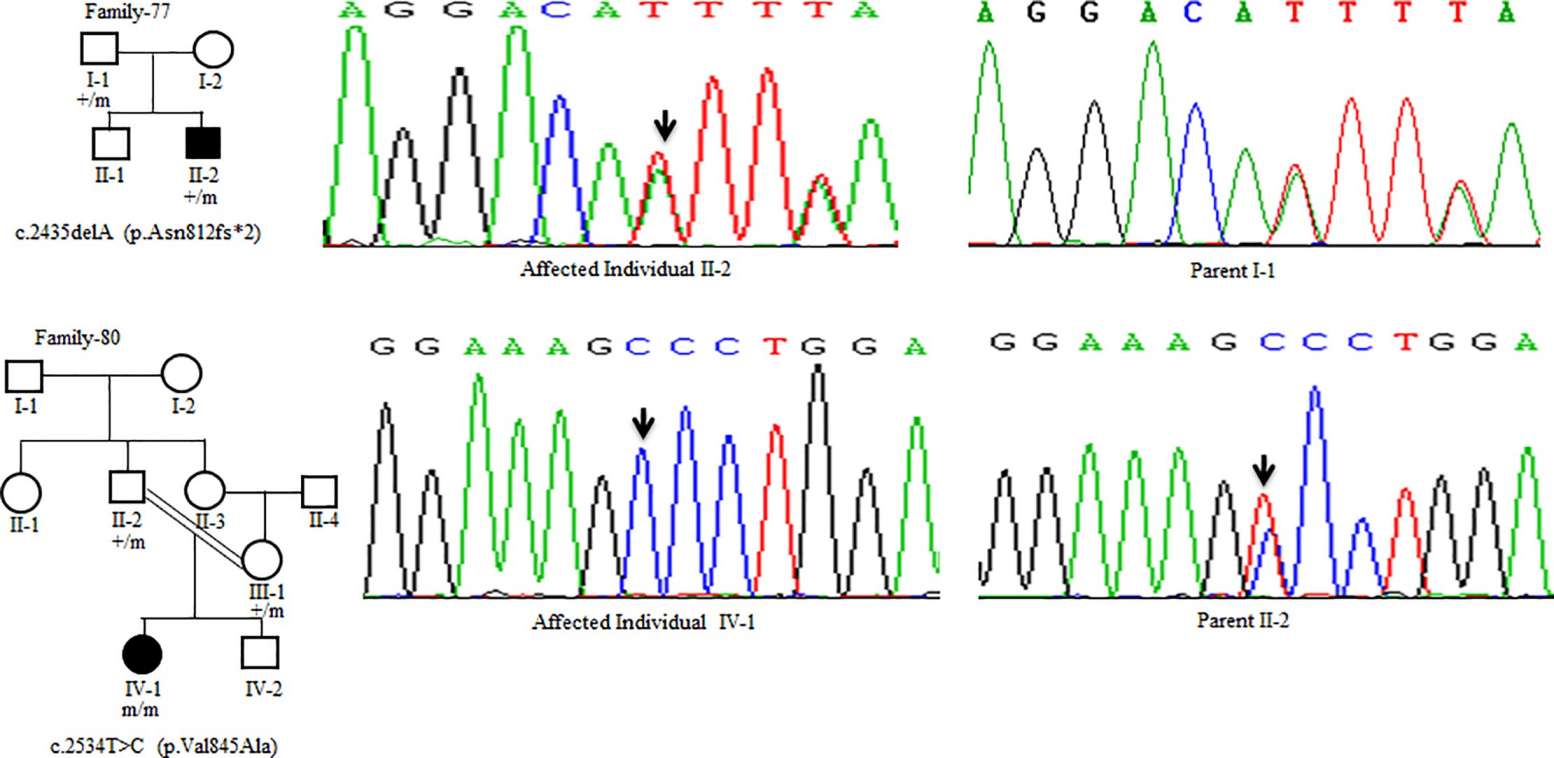
DNA sequence analysis of individuals from family-77 and family-80. (Upper panel) Sequencing chromatograms from the affected individual II-2 and parent I-1 from family-77. Arrows mark the deletion of the A residue in the affected individual II-2 and parent I-1 in a heterozygous state. (Lower panel) Sequencing chromatograms from the affected individual IV-1 and the parent II-2 from family-80. Arrows mark the T>C change in a homozygous state in the affected individual IV-1 and in a heterozygous state in the parent II-2. + and m denote the wild type and mutant alleles, respectively.

**Fig 6:**
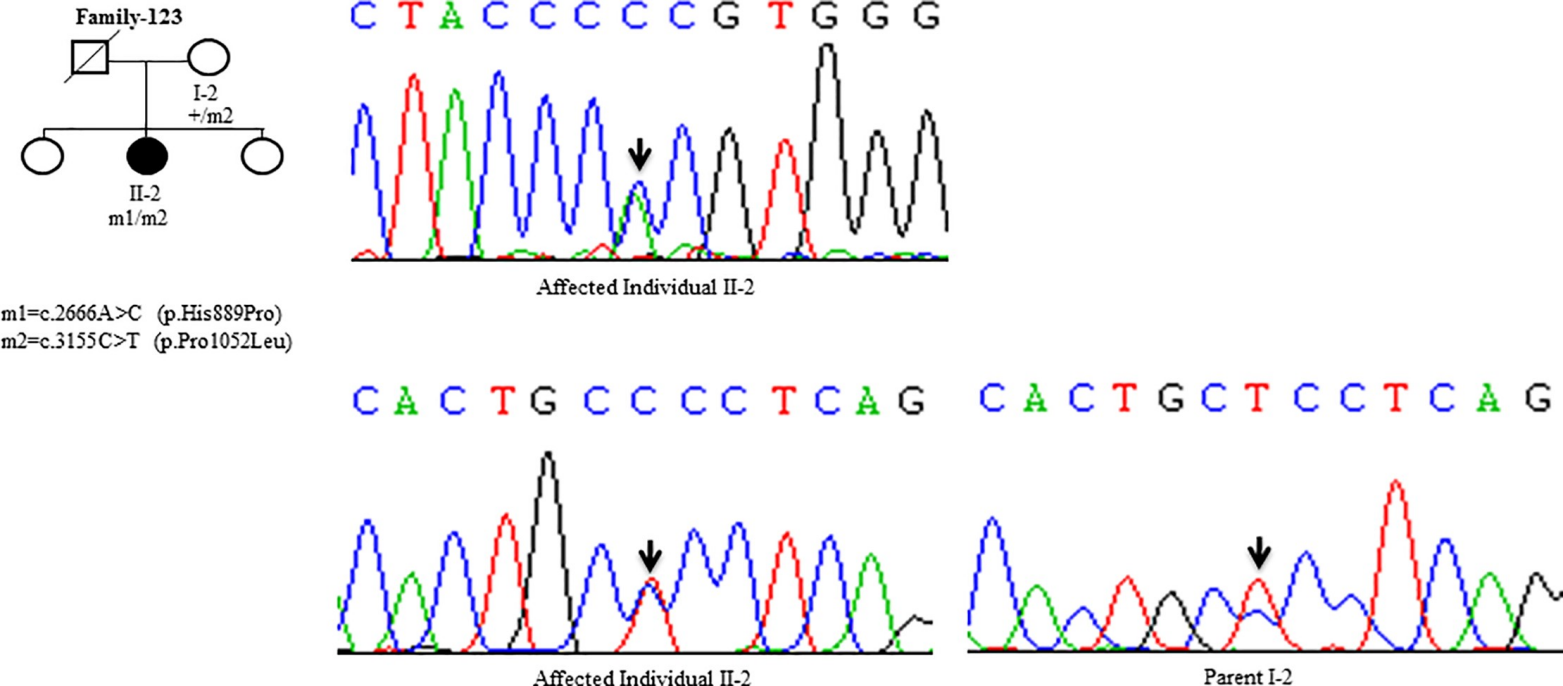
DNA sequence analysis of individuals from family-123. (Upper panel) Sequencing chromatogram from the affected individual II-2 from family-123. Arrow marks the A>C change in a heterozygous state in the affected individual II-2. (Lower panel) Sequencing chromatogram from the affected individual II-2 and the parent I-2 from family-123. Arrows mark the C>T change in a heterozygous state in the affected individual II-2 and parent I-2. + denotes the wild type allele. m1 and m2 denote different mutations.

**Fig 7:**
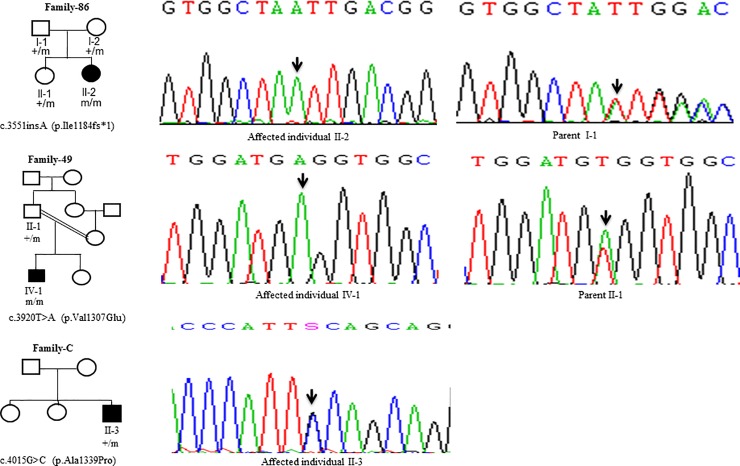
DNA sequence analysis of individuals from family-86, family-49 and family-C. (Upper panel) Sequencing chromatograms from the affected individual II-2 and the parent I-1 from family-86. Arrows mark the insertion of the A residue in a homozygous state in the affected individual II-2 and in a heterozygous state in the parent I-1. (Middle panel) Sequencing chromatograms of the affected individual IV-1 and the parent II-1 from family-49. Arrows mark the T>A change in a homozygous state in the affected individual IV-1 and in a heterozygous state in the parent II-1. (Lower panel) Sequencing chromatogram from the affected individual II-3 from family-C. Arrow marks the G>C change in a heterozygous state in the affected individual II-3. + and m denote the wild type and mutant alleles, respectively.

**Fig 8:**
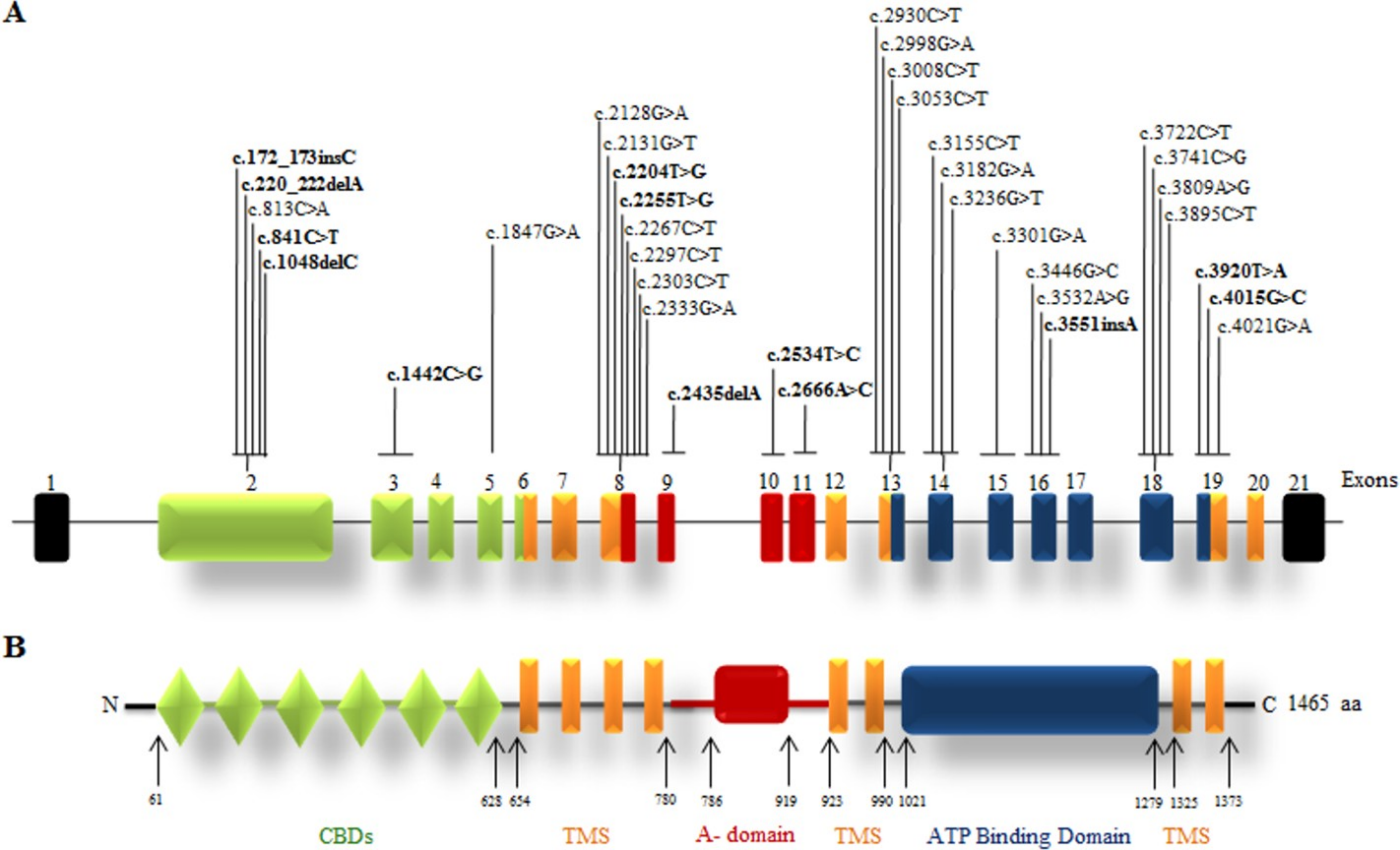
Mutation landscape of the *ATP7B* gene and protein. (A) The intron-exon structure of the gene. The novel mutations are shown in bold. (B) Different domains of the protein. Abbreviations: aa; amino acid; CBDs, copper binding domains; TMS, transmembrane segment and A-domain (actuator domain). The numbers refer to amino acid positions.

**Fig 9:**
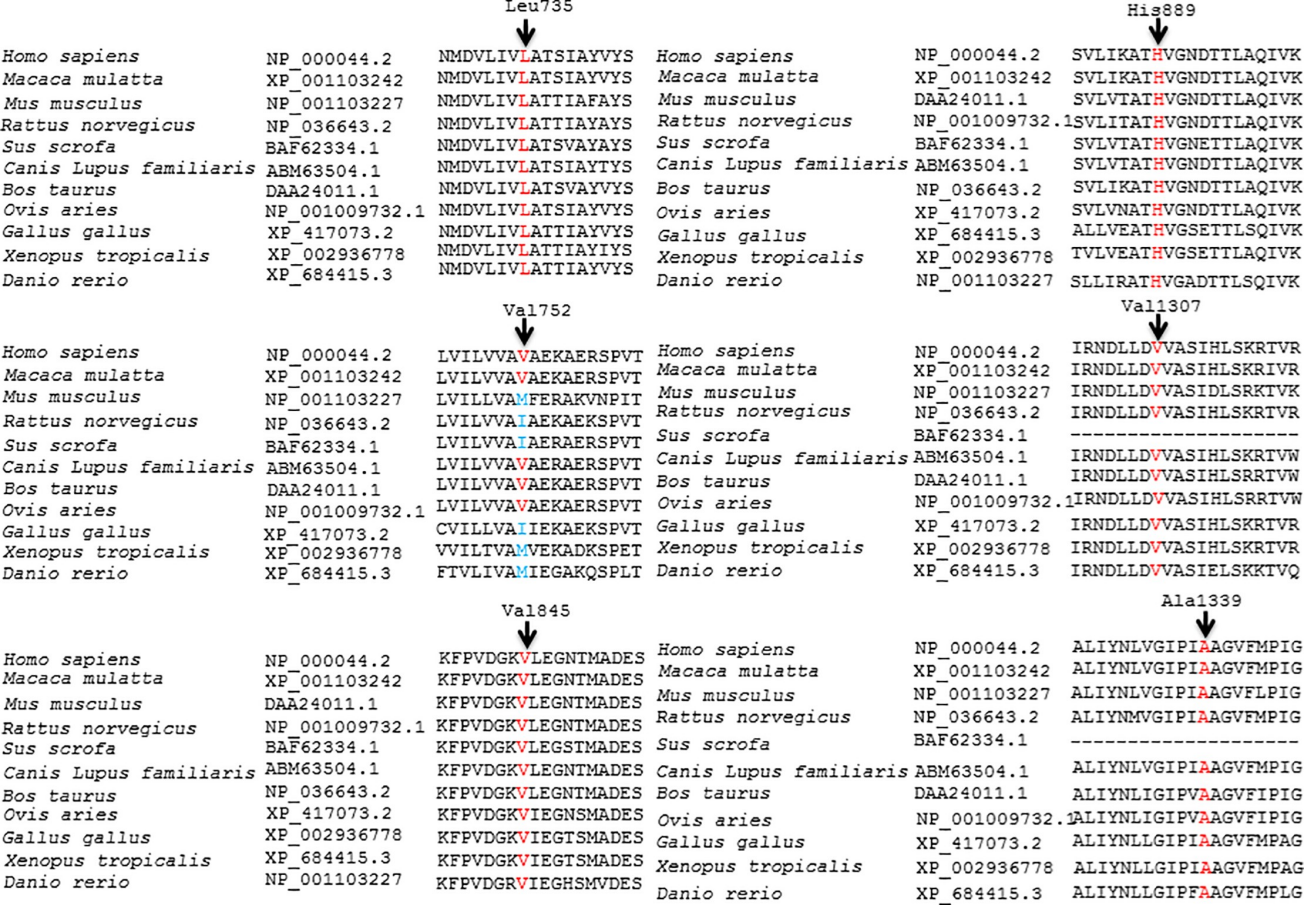
Conservation of the amino acid residues across different species in ATP7B. Arrows mark the conservation of amino acid residues Leu735, Val752, Val845, His889, Val1307, and Ala1339 across different species. GenBank accession numbers of ATP7B are also given.

**Fig 10:**
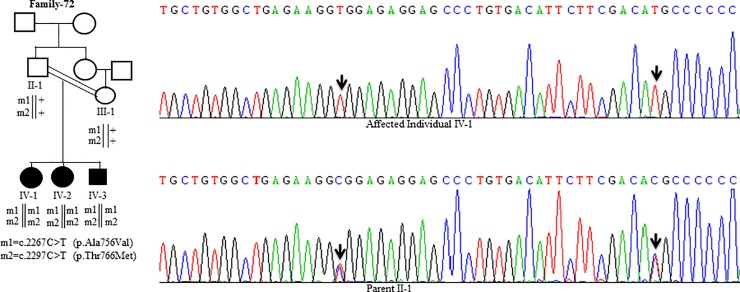
DNA sequence analysis of individuals from family-72. Sequencing chromatograms from the affected individual IV-1 and parent II-1. Arrows mark two different C>T changes in a homozygous state in the affected individual IV-1 and in a heterozygous state in the parent II-1. + denotes the wild-type allele. m1 and m2 denote two different mutant alleles.

Sanger Sequencing of the entire coding region of *ATP7B* in 102 WD families identified a total of 36 different mutations in 76/102 (74.51%) families, with a frequency ranging from 0.4% to 8.4% (Table 1). These mutations were missense, nonsense, insertions and deletions (Table 1). Of these, 13 were novel mutations [p.Ala58fs*19, p.Lys74fs*9, p.Gln281*, p.Pro350fs*12, p.Ser481*, p.Leu735Arg, p.Val752Gly, p.Asn812fs*2, p.Val845Ala, p.His889Pro, p.Ile1184fs*1, p.Val1307Glu and p.Ala1339Pro] (Table 1; Figs 1–7). Further, 28/36 (77.8%) mutations were missense, thus making them the most prevalent mutations identified in the present study. Based on this analysis, a mutational landscape was constructed, which depicts the spread of mutations across different exons (Fig 8A) and domains (Fig 8B) of *ATP7B*. Of 76 families with mutations, 20 families had a single mutation in a heterozygous condition, and thus the second mutation was not identified (Table 1). We used the following criteria to name a novel variant as a mutation. 1) The novel variant was segregating in the family. 2) The novel variant was not observed in 50 normal controls. 3) The novel variant was absent in the 1000 Genomes and ExAC databases. 4) The affected amino acid residue was conserved across species (Fig 9). 5) At least two of the following three mutation prediction programs, PolyPhen-2, Mutation Taster and SIFT, predicted a novel missense variant to be disease causing (Table F in S1 File). Interestingly, the mutation analysis of affected individuals in family-90 (Fig 1) and family-72 (Fig 10) identified two different homozygous mutations in each family, and thus each affected individual from these families harbored 2 mutations in each *ATP7B* allele.

**Table 1:** Mutations detected in the *ATP7B* gene in Wilson disease patients during present study.

Sl.#	Mutation	Exon(E)/ Intron(I)	Type of mutation	Novel/reported	Family (zygosity)	Region of protein	Ethnic origin	Frequency of mutation in affected individuals (%)	Frequency of mutation in other population (%)	Reference
1	c.172_173insC (p.Ala58fs*19)	E2	Insertion	Novel	Family-B (compound heterozygous with c.3741C>G) & Family-90 (homozygous)	CBD1	Indian (South)	1.3	-	This study
2	c.220_222delA (p.Lys74fs*9)	E2	Deletion	Novel	Family-128 (homozygous)	CBD1	Indian (South)	0.8	-	This study
3	c.813C>A (p.Cys271*)	E2	Nonsense	Reported	Family-N, Family-Q, Family-62, Family-75, Family-D, Family-69, Family-93 & Family-64 (homozygous); Family-A, Family-34 & Family-W (heterozygous)	CBD3	German, Turkish, Indian (East), Indian (South), Thai, Indian (West) & Indian (East)	8.4	1.2 (German), 1.9 (Turkish), 16 Indian (East), 10 Indian (South), 2.6 (Thai), 20.2 Indian (West) & 24.2 Indian (East)	http://www.wilsondisease.med.ualberta.ca/
4	c.841C>T (p.Gln281*)	E2	Nonsense	Novel	Family-31 (homozygous)	CBD3	Indian (South)	0.8	-	This study
5	c.1048delC (p.Pro350fs*12)	E2	Deletion	Novel	Family-60 (homozygous)	Between CBD3 & CBD4	Indian (South)	0.8	-	This study
6	c.1442C>G (p.Ser481*)	E3	Nonsense	Novel	Family-506 (heterozygous)	Between CBD4 & CBD5	Indian (South)	0.4	-	This study
7	c.1847G>A (p.Arg616Gln)	E5	Missense	Reported	Family-67 (heterozygous)	CBD6	British, Mediterranean, Indian (South) & Chinese–Han	0.4	1 (British), 6 (Mediterranean), 4 Indian (South) & 1 (Chinese-Han)	http://www.wilsondisease.med.ualberta.ca/
8	c.2128G>A (p.Gly710Ser)	E8	Missense	Reported	Family-56 (heterozygous)	TMS2	European, Middle Eastern, Hunagarian & Indian (East)	0.4	1.78 (European), <1 (Middle Eastern), 1.2 (Hunagarian) & 0.8% Indian (East)	http://www.wilsondisease.med.ualberta.ca/
9	c.2131G>T (p.Gly711Trp)	E8	Missense	Reported	Family-L (compound heterozygous with c.3809A>G); Family-419, Family-S, Family 42, Family-50, Family-53, Family 82 & Family-116 (homozygous); Family-74 (heterozygous)	TMS3	Pakistani	7.0	1.9 (Pakistani)	http://www.wilsondisease.med.ualberta.ca/
10	c.2204T>G (p.Leu735Arg)	E8	Missense	Novel	Family-M (homozygous)	TMS3	Indian (South)	0.8	-	This study
11	c.2255T>G (p.Val752Gly)	E8	Missense	Novel	Family-41B (compound heterozygous with c.3182G>A)	TMS3	Indian (South)	0.4	-	This study
12	c.2267C>T (p.Ala756Val)	E8	Missense	Reported	Family-72 (homozygous)	Between TMS3 & TMS4	Italian	0.8	16.6 (Italian)	http://www.wilsondisease.med.ualberta.ca/
13	c.2297C>T (p.Thr766Met)	E8	Missense	Reported	Family-72 (homozygous)	TMS4	British	0.8	0.2 (British)	http://www.wilsondisease.med.ualberta.ca/
14	c.2303C>T (p.Pro768Leu)	E8	Missense	Reported	Family-55 (compound heterozygous with c.3446G>C)	TMS4	Indian (South) & Spanish	0.4	7.4 Indian (South) & 3.3 Spanish	http://www.wilsondisease.med.ualberta.ca/
15	c.2333G>A (p.Arg778Gln)	E8	Missense	Reported	Family-P & Family-85 (homozygous)	TMS4	Taiwanese, Chinese, Indian (West) & Indian (East)	1.7	4.8 (Taiwanese), 1.5 Chinese,1 Indian (West) & 1 Indian (East)	http://www.wilsondisease.med.ualberta.ca/
16	c.2435delA (p.Asn812fs*2)	E9	Deletion	Novel	Family-77 (heterozygous)	A domain	Indian (South)	0.4	-	This study
17	c.2534T>C (p.Val845Ala)	E10	Missense	Novel	Family-80 (homozygous)	A domain	Indian (South)	0.8	-	This study
18	c.2666A>C (p.His889Pro)	E11	Missense	Novel	Family-123 (compound heterozygous with c.3155C>T)	A domain	Indian (South)	0.4	-	This study
19	c.2930C>T (p.Thr977Met)	E13	Missense	Reported	Family-76 (homozygous)	TMS6	European, British & Indian (East)	0.8	5.3 European, 2.8 British, 0.5 Indian (East) & 1 Indian (East)	http://www.wilsondisease.med.ualberta.ca/
20	c.2998G>A (p.Gly1000Arg)	E13	Missense	Reported	Family-46 & Family-118 (homozygous)	Between TMS6 & ATP binding domain	British & Sardinian	1.7	1.6 (British) & 0.4 (Sardinian)	http://www.wilsondisease.med.ualberta.ca/
21	c.3008C>T (p.Ala1003Val)	E13	Missense	Reported	Family-36, Family-54, Family-83 & Family-100 (homozygous)	Between TMS6 & ATP binding domain	Turkish, Indian (South) & Indian (West)	3.5	1 (Turkish), 9.2 Indian (South) & 11 Indian (West)	http://www.wilsondisease.med.ualberta.ca/
22	c.3053C>T (p.Ala1018Val)	E13	Missense	Reported	Family-63 (compound heterozygous with c.3809A>G); Family-121 (Heterozygous)	Between TM6 & ATP binding domain	Czech, Chinese–Han & Sardinian	0.8	0.2 (Czech), 0.7 (Chinese–Han) & 1.3 (Sardinian)	http://www.wilsondisease.med.ualberta.ca/
23	c.3155C>T (p.Pro1052Leu)	E14	Missense	Reported	Family-123 (compound heterozygous with c.2666A>C)	ATP binding domain	British	0.4	0.9 (British)	http://www.wilsondisease.med.ualberta.ca/
24	c.3182G>A (p.Gly1061Glu)	E14	Missense	Reported	Family-O, Family-V, Family-58 & Family-101 (homozygous); Family-38 & Family-78 (heterozygous); Family-41B (compound heterozygous with c.2255T>G)	ATP binding domain	Pakistani, Turkish, Indian (South), Indian (East), Indian (North) & Indian (West)	4.8	1.9 (Pakistani), 3 (Turkish), 3.7 Indian (South), 11 Indian (East), 3.3 Indian (North) & 3 Indian (West)	http://www.wilsondisease.med.ualberta.ca/
25	c.3236G>T (p.Cys1079Phe)	E14	Missense	Reported	Family-73 (homozygous)	ATP binding domain	Chinese	0.8	0.6 (Chinese)	http://www.wilsondisease.med.ualberta.ca/
26	c.3301G>A (p.Gly1101Arg)	E15	Missense	Reported	Family-32, Family-43, Family-52, Family-122 & Family-40 (homozygous)	ATP binding domain	Indian (West) & Indian (East)	4.4	3 Indian (West) & 23 Indian (East)	http://www.wilsondisease.med.ualberta.ca/
27	c.3446G>C (p.Gly1149Ala)	E16	Missense	Reported	Family-T, Family-35 & Family-47 (homozygous); Family-55 (compound heterozygous with c.2303C>T)	ATP binding domain	Filipino	3.0	0.1 (Filipino)	http://www.wilsondisease.med.ualberta.ca/
28	c.3532A>G (p.Thr1178Ala)	E16	Missense	Reported	Family-59 & Family-91 (homozygous); Family-E, Family-G & Family-44 (heterozygous)	ATP binding domain	Taiwanese, Indian (East), Chinese & Indian (East)	3.0	3.4 (Taiwanese), 0.57 Indian (East), 5.5 (Chinese) & 0.5 Indian (East)	http://www.wilsondisease.med.ualberta.ca/
29	c.3551insA (p.Ile1184fs*1)	E16	Insertion	Novel	Family-86 (homozygous)	ATP binding domain	Indian (South)	0.8	-	This study
30	c.3722C>T (p.Ala1241Val)	E18	Missense	Reported	Family-98 (heterozygous)	ATP binding domain	Indian (South) & Indian (East)	0.4	1.8 Indian (South) & 5 Indian (East)	http://www.wilsondisease.med.ualberta.ca/
31	c.3741C>G (p.His1247Gln)	E18	Missense	Reported	Family-B (compound heterozygous with c.172_173insC); Family-90 (homozygous)	ATP binding domain	Indian (East)	1.3	0.2 Indian (East)	http://www.wilsondisease.med.ualberta.ca/
32	c.3809A>G (p.Asp1270Ser)	E18	Missense	Reported	Family-89 (homozygous); Family-L (compound heterozygous with c.2131G>T) & Family-63 (compound heterozygous with c.3053C>T)	ATP binding domain	Indian (South), Indian (East), Chinese, Indian (North) & Indian (West)	1.7	5.5 Indian (South), 1.7 Indian (East), 0.8 (Chinese), 2.2 Indian (North) & 3 Indian (West)	http://www.wilsondisease.med.ualberta.ca/
33	c.3895C>T (p.Leu1299Phe)	E18	Missense	Reported	Family-U & Family-70 (heterozygous)	Between ATP binding domain & TMS7	Indian (South) & Indian (West)	0.8	1.8 Indian (South) & 3 Indian (West)	http://www.wilsondisease.med.ualberta.ca/
34	c.3920T>A (p.Val1307Glu)	E19	Missense	Novel	Family-49 (homozygous)	Between ATP binding domain & TMS7	Indian (South)	0.8	-	This study
35	c.4015G>C (p.Ala1339Pro)	E19	Missense	Novel	Family-C (heterozygous)	TMS7	Indian (South)	0.4	-	This study
36	c.4021G>A (p.Gly1341Ser)	E19	Missense	Reported	Family-K (homozygous); Family-Y & Family-57 (heterozygous)	TMS7	Indian (South) & Indian (West)	1.7	3.7 Indian (South) & 1 Indian (West)	http://www.wilsondisease.med.ualberta.ca/

No mutations were detected in the coding or untranslated regions of the *ATP7B* gene in 26/102 (25.49%) families. Our inability to detect mutations in these families suggests that the mutations could be present in deep intronic regions or there could be large insertions, deletions and duplications which are not amenable to Sanger sequencing. Several non-disease-causing variants were also observed in different families (Table G in S1 File).

Several studies on the mutation analysis of *ATP7B* in WD patients have been reported from India [8–10, 12–15]. However, only a few large cohort studies have been reported from India. Three such studies by Gupta et al. [8], Mukherjee et al. [10], and Gupta et al. [12], which included 109, 119, and 62 WD families respectively, were reported from the eastern Indian population. Similarly, a study of 52 WD families was reported from the western Indian population [9]. This is the first largest cohort study of 102 WD families from a south Indian population, which has identified 36 different mutations, including 13 novel ones. Moreover, 15/36 known mutations were reported previously from the north, south, east and west Indian populations [9–10, 12, 14–15], whereas 8/36 mutations identified in the present study have never been reported previously from the Indian population (Table 1). The difference in mutations identified from different regions of India might be due to geographic, ethnic, and genetic differences in the Indian population. Therefore, a genetic study performed in a region of India cannot be extrapolated to the other parts of the country.

Genotype-phenotype correlation studies help in understanding the molecular mechanism of a disease, predict its course of progression and provide better monitoring of the individuals at risk. Our genotype-phenotype analysis suggested that clinical features such as dystonia, rigidity, bradykinesia, hepatomegaly and splenomegaly are significantly more associated with patients having truncating than those with missense mutations (Table H in S1 File). For example, 20/45 (44.44%) patients with missense mutations have dystonia, whereas 11/12 (91.66%) patients with truncating mutation have it (Table H in S1 File). Further, tremor is significantly more associated with patients having missense than those with truncating mutations (Table H in S1 File). No significant difference is observed between patients with missense or truncating mutations and other clinical features (Table H in S1 File).

There are a very few reports on the genotype-phenotype correlations in WD patients [16–18]. One of the best-known examples of the genotype-phenotype correlation in WD patients is the high frequency mutation p.H1069Q, which is associated with late-onset neurological symptoms in the European population [17]. Similar to the present study, no significant correlations were seen between the phenotypes like KF ring and ceruloplasmin levels and genotypes in another study [18]. Interestingly, differences in clinical features were seen even in patients with the same mutation, suggesting that other factors like environment, genetic, and epigenetic changes might affect the disease outcome [19]. It is interestingly to note that affected individuals from family-35, family-54 and family-73 have a normal ceruloplasmin level of 16, 18 and 28 mg/dL, respectively (Table I in S1 File). However, this finding is not surprising as around 5% of affected individuals homozygous for *ATP7B* mutations have normal ceruloplasmin levels [20].

Founder mutations help in tracing the evolutionary aspect of a disease, evolution of human population, its migration and growth [21]. Interestingly, in a country like India, it is seen that founder mutations contribute more to the disease burden of recessive disorders than consanguinity [22]. This is mainly because of the traditional practice of same-caste marriages in Indian population which contribute to the presence of founder mutations [23]. In the present study, out of the 36 mutations identified, 14 (38.8%) were recurrent (Table 1). This prompted us to look into the possible founder effect for these 14 recurrent mutations. The haplotype analysis, using microsatellite and SNP (single nucleotide polymorphism) markers, suggested that all the 14 mutations have founder effects (Figures B-O in S1 File). For example, family-B and family-90 with p.Ala58fs*19 share a common disease haplotype of 632.8 Kb (Figure B in S1 File). Length of the disease haplotype ranged from 45.8 kb for p.Asp1270Ser (Figure M in S1 File) to 698.2 kb for p.Cys271*, p.Gly711Trp, p.Ala1003Val, p.Gly1061Glu, p.Gly1101Arg, p.Gly1149Ala, p.Thr1178Ala, p.His1247Gln and p.Leu1299Phe mutations (Figures C-D, G-L and N in S1 File). Further, the mutation analysis identified c.813C>A (p.Cys271*) as the most common mutation with an allele frequency of 8.4% (Table 1). A common haplotype was identified in all the 11 families carrying this mutation (Figure C in S1 File). Our data thus add up to the previous studies from India where c.813C>A (p.Cys271*) mutation was identified as the most prevalent mutation with an allele frequency of 16%, 10%, and 20.2% in eastern, southern, and western Indian populations respectively [9,12,14].

In summary, this is the first study on the genetic analysis of a large cohort of 102 WD families from a south Indian population, predominantly from the state of Karnataka. Of 36 mutations identified in 76/102 families, 13 (36.1%) were novel, and thus the total number of *ATP7B* mutations increases to 758 (Table J in S1 File). We hope that the mutations identified during the present study will facilitate carrier and pre-symptomatic detection, and prenatal genetic diagnosis in affected families.

## Supporting information

S1 File Supporting multiple Figures and Tables. (PDF)

## References

[1] Ala A, Borjigin J, Rochwarger A, Schilsky M. Wilson disease in septuagenarian siblings: raising the bar for diagnosis. Hepatology. 2005;4: 668–70. 10.1002/hep.20601 15723329

[2] Perri RE, Hahn SH, Ferber MJ, Kamath PS. Wilson disease-keeping the bar for diagnosis raised. Hepatology. 2005;42: 974. 10.1002/hep.20893 16175588

[3] Wiernicka A, Dądalski M, Jańczyk W, Kamińska D, Naorniakowska M, Hüsing-Kabar A, et al Early onset of Wilson disease: diagnostic challenges. J. Pediatr. Gastroenterol. Nutr. 2017; 65: 555–560. 10.1097/MPG.0000000000001700 28753182

[4] Bull PC, Thomas GR, Rommens JM, Forbes JR, Cox DW. The Wilson disease gene is a putative copper transporting P-type ATPase similar to the Menkes gene. Nat. Genet. 1993;5: 327–337. 10.1038/ng1293-327 8298639

[5] Tanzi RE, Petrukhin K, Chernov I, Pellequer JL, Wasco W, Ross B et al The Wilson disease gene is a copper transporting ATPase with homology to the Menkes disease gene. Nat. Genet. 1993;5: 344–350. 10.1038/ng1293-344 8298641

[6] Yamaguchi Y, Heiny ME, Gitlin JD. Isolation and characterization of a human liver cDNA as a candidate gene for Wilson disease. Biochem. Biophys. Res. Commun. 1993;197: 271–277. 10.1006/bbrc.1993.2471 8250934

[7] Polishchuk EV, Concilli M, Iacobacci S, Chesi G, Pastore N, Piccolo P et al Wilson disease protein ATP7B utilizes lysosomal exocytosis to maintain copper homeostasis. Dev. Cell. 2014;29: 686–700. 10.1016/j.devcel.2014.04.033 24909901 PMC4070386

[8] Gupta A, Chattopadhyay I, Dey S, Nasipuri P, Das SK, Gangopadhyay PK et al Molecular pathogenesis of Wilson disease among Indians: a perspective on mutation spectrum in *ATP7B* gene, prevalent defects, clinical heterogeneity and implication towards diagnosis. Cell. Mol. Neurobiol. 2007;27: 1023–1033. 10.1007/s10571-007-9192-7 17823867 PMC11517134

[9] Aggarwal A, Chandhok G, Todorov T, Parekh S, Tilve S, Zibert A et al Wilson disease mutation pattern with genotype-phenotype correlations from Western India: confirmation of p.C271* as a common Indian mutation and identification of 14 novel mutations. Ann. Hum. Genet. 2013;77: 299–307. 10.1111/ahg.12024 23551039

[10] Mukherjee S, Dutta S, Majumdar S, Biswas T, Jaiswal P, Sengupta M et al Genetic defects in Indian Wilson disease patients and genotype-phenotype correlation. Parkinsonism. Relat. Disord. 2014;20: 75–81. 10.1016/j.parkreldis.2013.09.021 24094725

[11] Kumar A, Blanton SH, Babu M, Markandaya M, Girimaji SC. Genetic analysis of primary microcephaly in Indian families: novel *ASPM* mutations. Clin. Genet. 2004; 66: 341–348. 10.1111/j.1399-0004.2004.00304.x 15355437

[12] Gupta A, Aikath D, Neogi R, Datta S, Basu K, Maity B et al Molecular pathogenesis of Wilson disease: haplotype analysis, detection of prevalent mutations and genotype–phenotype correlation in Indian patients. Hum. Genet. 2005;118: 49–57. 10.1007/s00439-005-0007-y 16133174

[13] Kumar S, Thapa BR, Kaur G, Prasad R. Identification and molecular characterization of 18 novel mutations in the *ATP7B* gene from Indian Wilson disease patients: genotype. Clin. Genet. 2005;67: 443–445. 10.1111/j.1399-0004.2005.00440.x 15811015

[14] Santhosh S, Shaji RV, Eapen CE, Jayanthi V, Malathi S, Chandy M et al *ATP7B* mutations in families in a predominantly southern Indian cohort of Wilson's disease patients. Indian J. Gastroenterol. 2006;25: 277–282. 17264425

[15] Khan S, Behari M, Vivekanandhan S, Goyal V, Thelma BK. Mutation profile in Wilson’s disease from north Indian patients. Int. J. Hum. Genet. 2012;12: 125–132.

[16] Ferenci P. Phenotype-genotype correlations in patients with Wilson’s disease. Ann. N.Y. Acad. Sci. 2014;1315: 1–5. 10.1111/nyas.12340 24517292

[17] Stapelbroek JM, Bollen CW, van Amstel JK, van Erpecum KJ, van Hattum J, van den Berg LH et al The H1069Q mutation in *ATP7B* is associated with late and neurologic presentation in Wilson disease: results of a meta-analysis. J. Hepatol. 2004;41: 758–763. 10.1016/j.jhep.2004.07.017 15519648

[18] Guggilla SR, Senagari JR, Rao PN, Madireddi S. Spectrum of mutations in the ATP binding domain of *ATP7B* gene of Wilson disease in a regional Indian cohort. Gene. 2015;569: 83–87. 10.1016/j.gene.2015.05.031 25982861

[19] Simon I, Schaefer M, Reichert J, Stremmel W. Analysis of the human Atox 1 homologue in Wilson patients. World. J. Gastroenterol. 2008;14: 2383–2387. 10.3748/wjg.14.2383 18416466 PMC2705094

[20] Steindl P, Ferenci P, Dienes HP, Grimm G, Pabinger I, Madl C et al Wilson’s disease in patients presenting with liver disease: a diagnosis challenge. Gastroenterol. 1997;113: 212–218. 10.1016/s0016-5085(97)70097-0 9207280

[21] Drayna D. Founder mutations. Sci. Am. 2005;293: 78–85. 10.1038/scientificamerican1005-78 16196257

[22] Reich D, Thangaraj K, Patterson N, Price AL, Singh L. Reconstructing Indian population history. Nature. 2009;461: 489–494. 10.1038/nature08365 19779445 PMC2842210

[23] Ankala A, Tamhankar PM, Valencia CA, Rayam KK, Kumar MM, Hegde MR. Clinical applications and implications of common and founder mutations in Indian subpopulations. Hum. Mutat. 2015;36: 1–10. 10.1002/humu.22704 25323826

