# Supplementary material for: Genetic analysis of ATP7B in 102 south Indian families with Wilson disease
Source: PLoS One. 2019 May 6;14(5):e0215779. doi: 10.1371/journal.pone.0215779 (PMC6502322; doi:10.1371/journal.pone.0215779)
Supplement: S1 File — (PDF) [file pone.0215779.s001.pdf]

S1 File: Supporting multiple Figures and Tables

Genetic analysis of *ATP7B* in 102 south Indian families with Wilson disease

Nivedita Singh[1], Pradeep Kallollimath[2], Mohd Hussain Shah[1], Saketh Kapoor[1], Vishwanath Kumble Bhat[1], Lakshminarayanapuram Gopal Viswanathan [2], Madhu Nagappa[2], Parayil S. Bindu[2], Arun B. Taly[2], Sanjib Sinha[2], Arun Kumar[1*]

[1]Department of Molecular Reproduction, Development and Genetics, Indian Institute of Science, Bangalore, India

[2]Department of Neurology, National Institute of Mental Health and Neuro Sciences, Bangalore, India

*Corresponding author

Email: arunk@iisc.ac.in

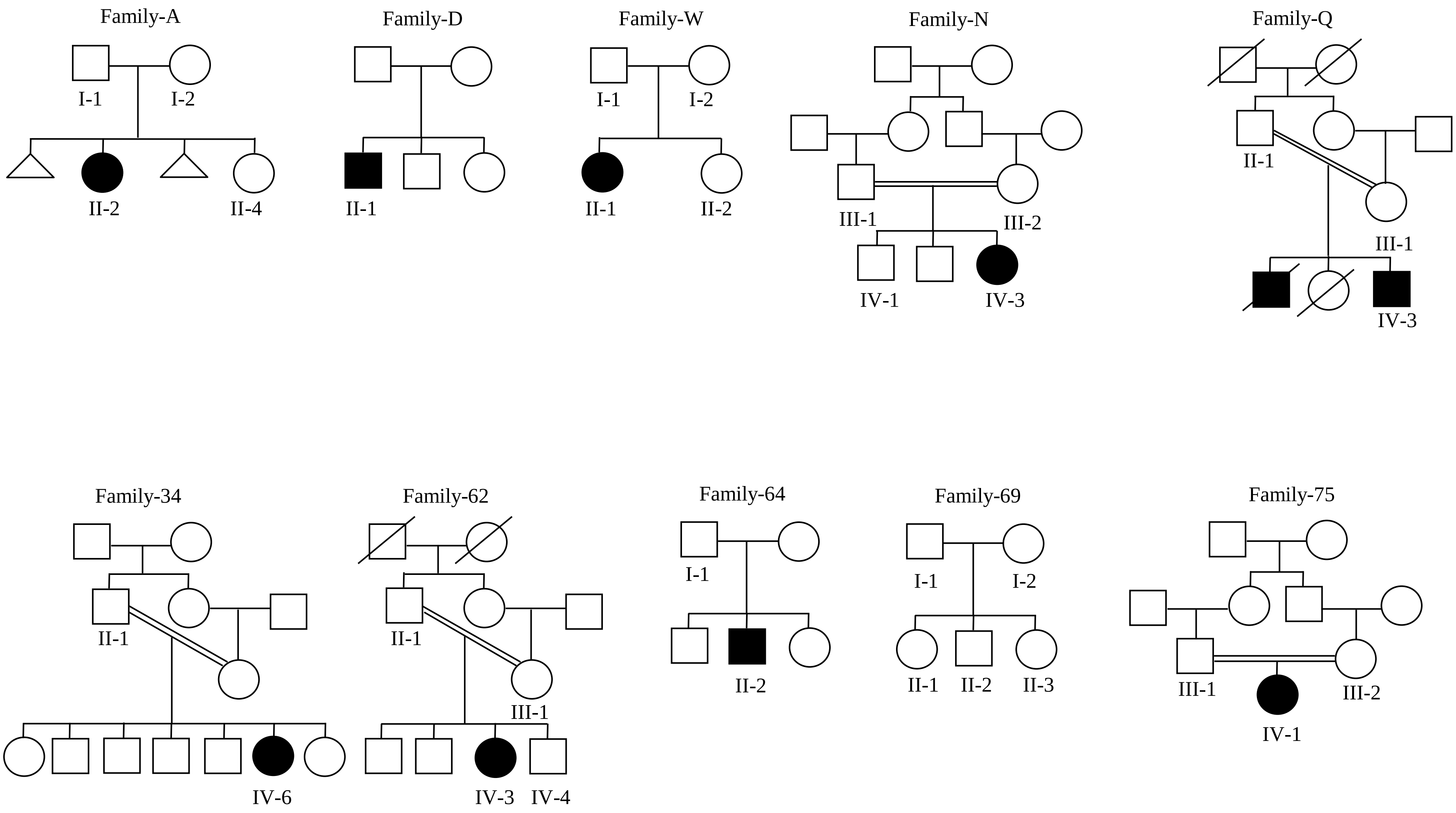

Family-A
I-1
I-2
II-2
II-4
Family-D
II-1
Family-W
I-1
I-2
II-1
II-2
Family-N
III-1
III-2
IV-1
IV-3
Family-Q
II-1
III-1
IV-3
Family-34
II-1
IV-6
Family-62
II-1
III-1
IV-3
IV-4
Family-64
I-1
II-2
Family-69
I-1
I-2
II-1
II-2
II-3
Family-75
III-1
III-2
IV-1

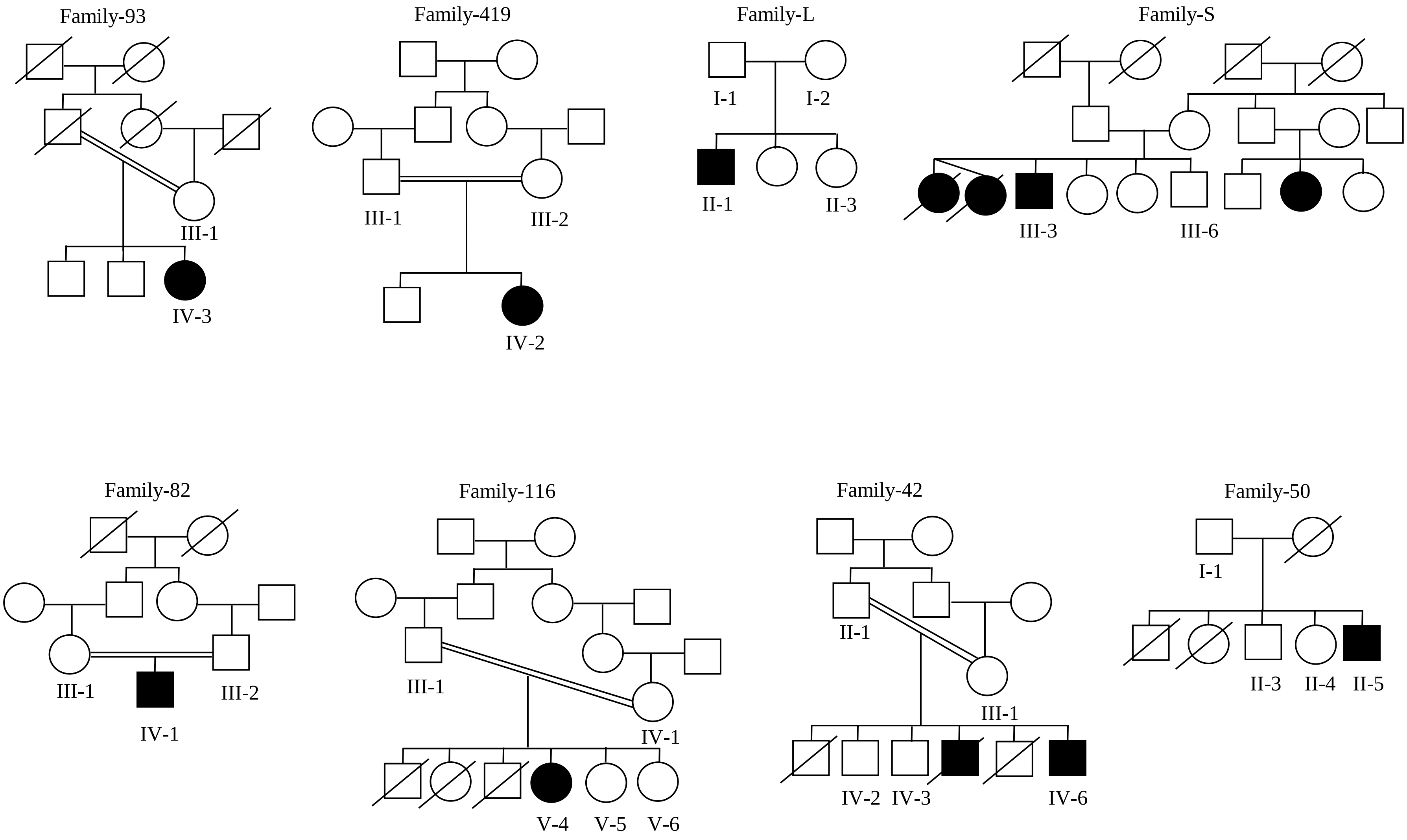
Family-93
III-1
IV-3
Family-419
III-1
III-2
IV-2
Family-L
I-1
I-2
II-1
II-3
Family-S
III-3
III-6
Family-82
III-1
III-2
IV-1
Family-116
III-1
IV-1
V-4
V-5
V-6
Family-42
II-1
III-1
IV-2
IV-3
IV-6
Family-50
I-1
II-3
II-4
II-5

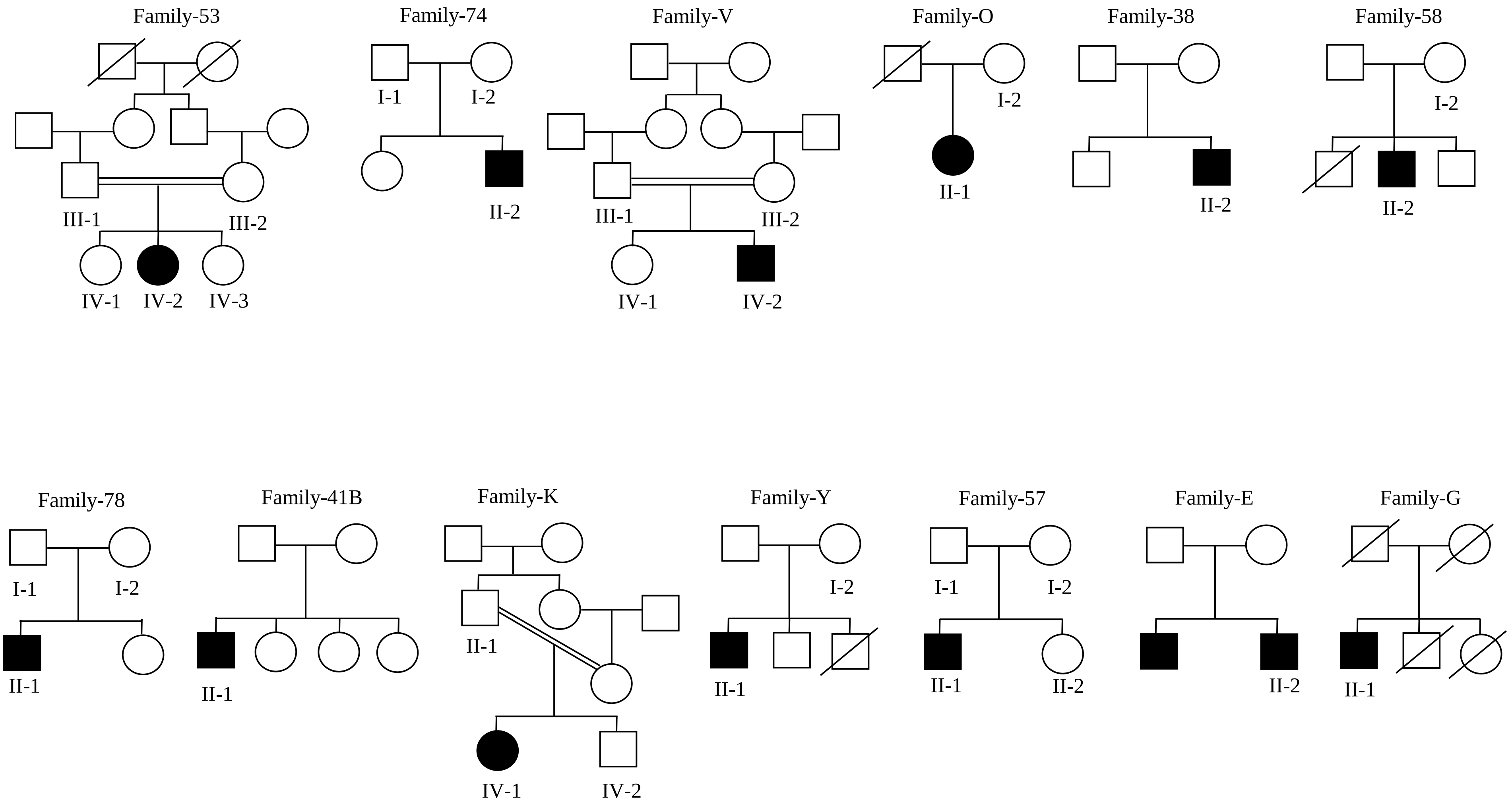
Family-53
III-1
III-2
IV-1
IV-2
IV-3
Family-74
I-1
I-2
II-2
Family-V
III-1
III-2
IV-1
IV-2
Family-O
I-2
II-1
Family-38
II-2
Family-58
I-2
II-2
Family-78
I-1
I-2
II-1
Family-41B
II-1
Family-K
II-1
IV-1
IV-2
Family-Y
I-2
II-1
Family-57
I-1
I-2
II-1
II-2
Family-E
II-2
Family-G
II-1

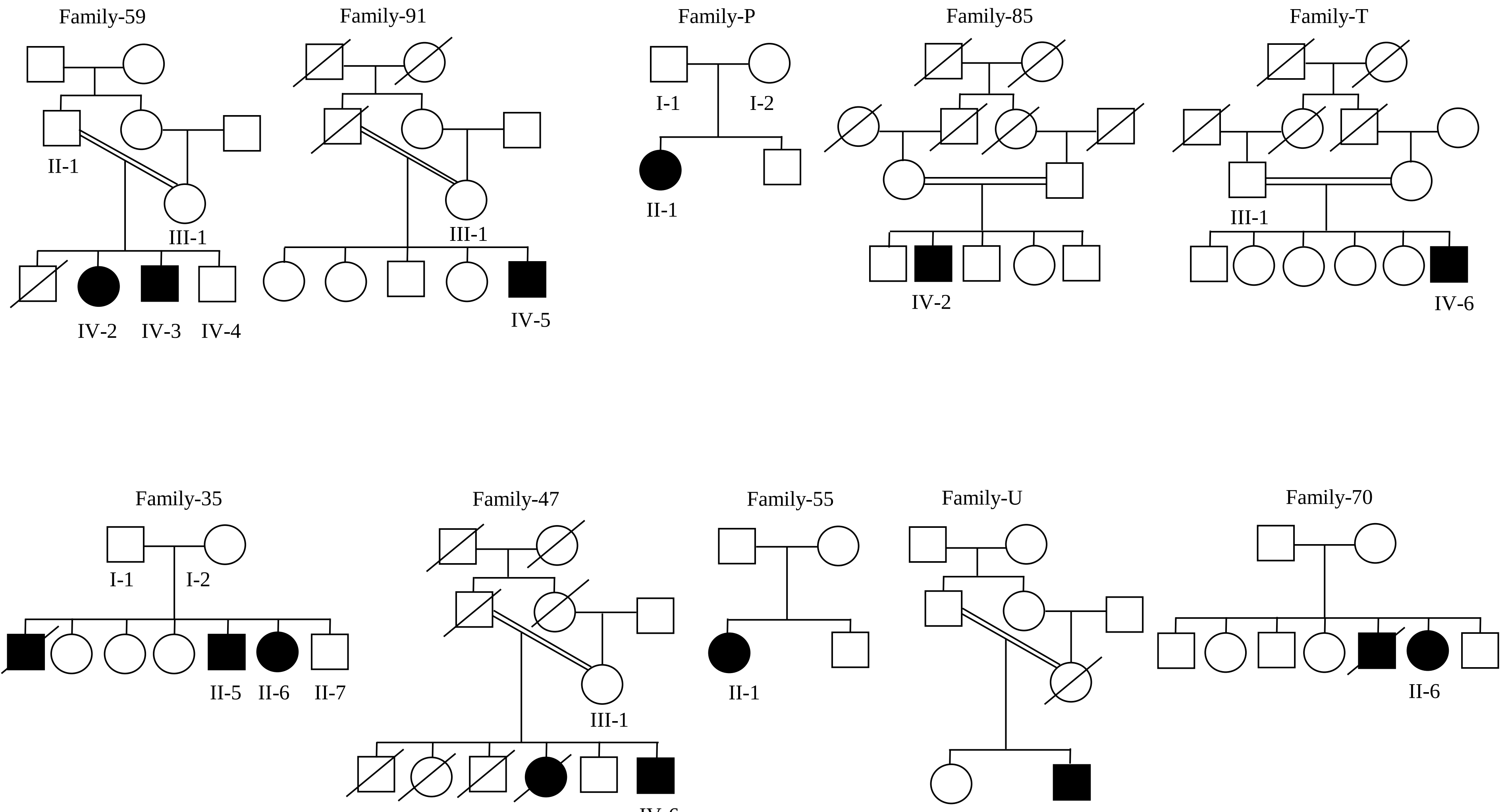
Family-59
II-1
III-1
IV-2
IV-3
IV-4
Family-91
III-1
IV-5
Family-P
I-1
I-2
II-1
Family-85
IV-2
Family-T
III-1
IV-6
Family-35
I-1
I-2
II-5
II-6
II-7
Family-47
III-1
IV-6
Family-55
II-1
Family-U
IV-2
Family-70
II-6

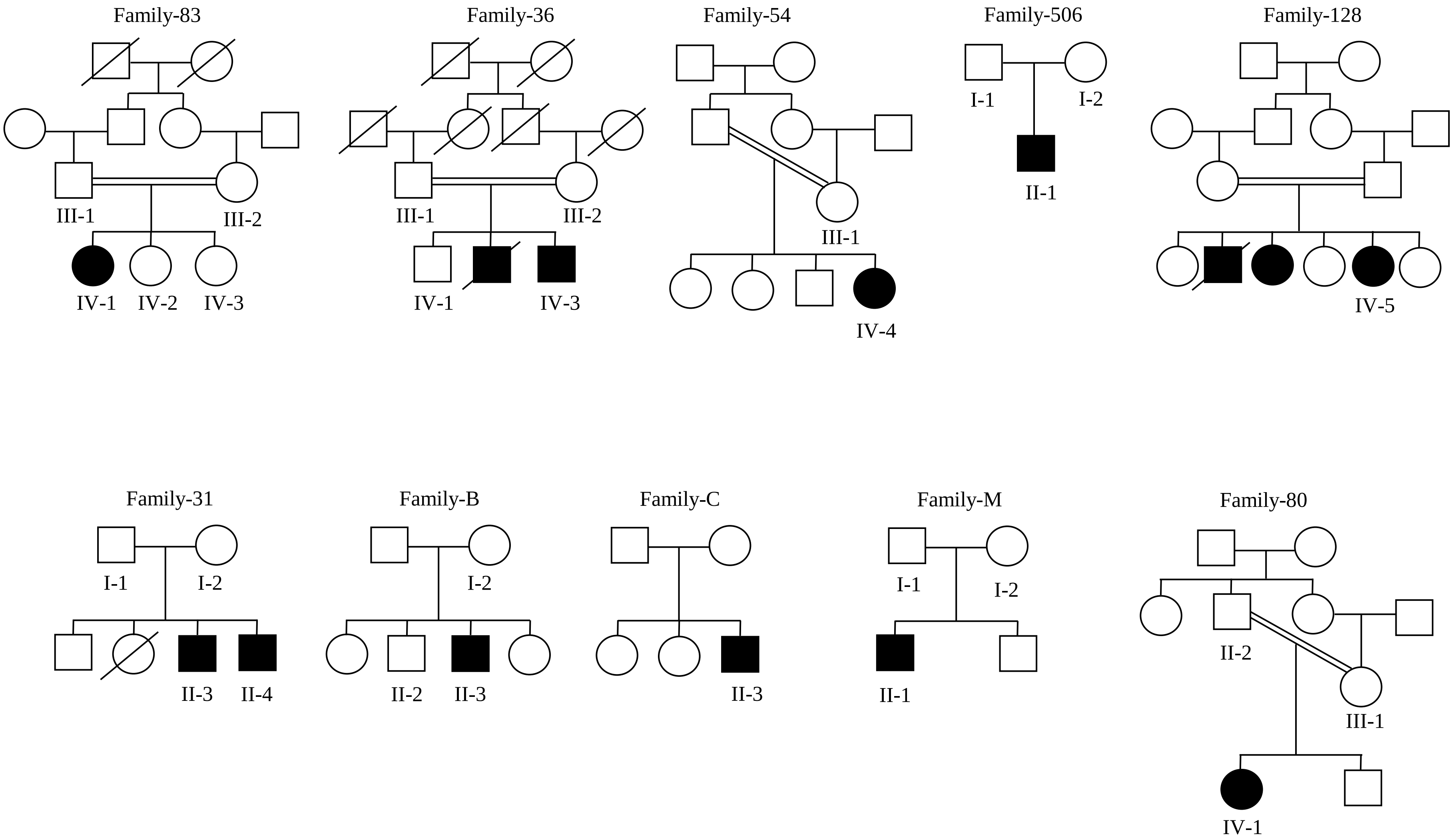
Family-83
III-1
III-2
IV-1
IV-2
IV-3
Family-36
III-1
III-2
IV-1
IV-3
Family-54
III-1
IV-4
Family-506
I-1
I-2
II-1
Family-128
IV-5
Family-31
I-1
I-2
II-3
II-4
Family-B
I-2
II-2
II-3
Family-C
II-3
Family-M
I-1
I-2
II-1
Family-80
II-2
III-1
IV-1

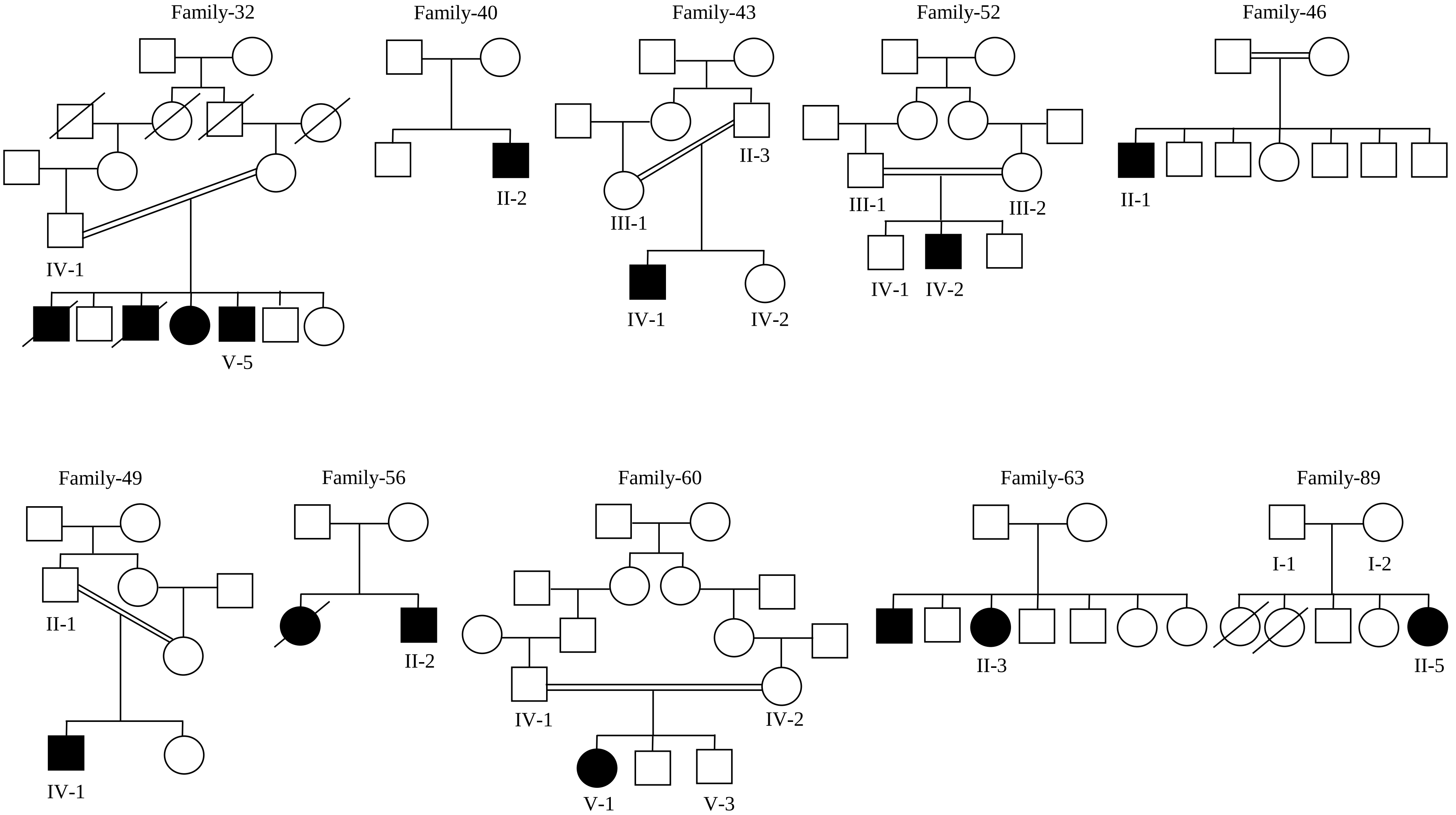
Family-32
IV-1
V-5
Family-40
II-2
Family-43
II-3
III-1
IV-1
IV-2
Family-52
III-1
III-2
IV-1
IV-2
Family-46
II-1
Family-49
II-1
IV-1
Family-56
II-2
Family-60
IV-1
IV-2
V-1
V-3
Family-63
II-3
Family-89
I-1
I-2
II-5

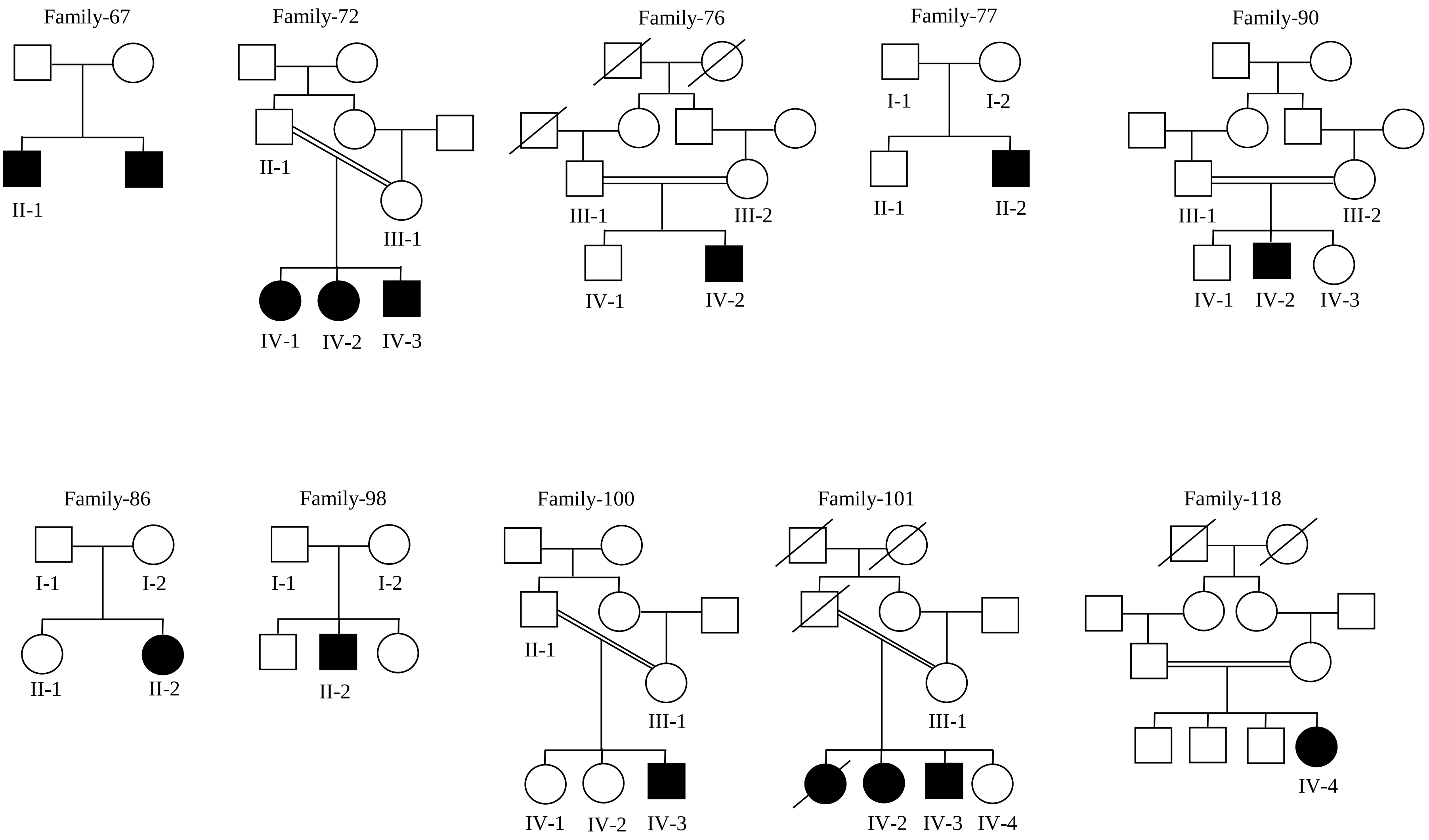

Family-67
II-1
Family-72
II-1
III-1
IV-1
IV-2
IV-3
Family-76
III-1
III-2
IV-1
IV-2
Family-77
I-1
I-2
II-1
II-2
Family-90
III-1
III-2
IV-1
IV-2
IV-3
Family-86
I-1
I-2
II-1
II-2
Family-98
I-1
I-2
II-2
Family-100
II-1
III-1
IV-1
IV-2
IV-3
Family-101
III-1
IV-2
IV-3
IV-4
Family-118
IV-4

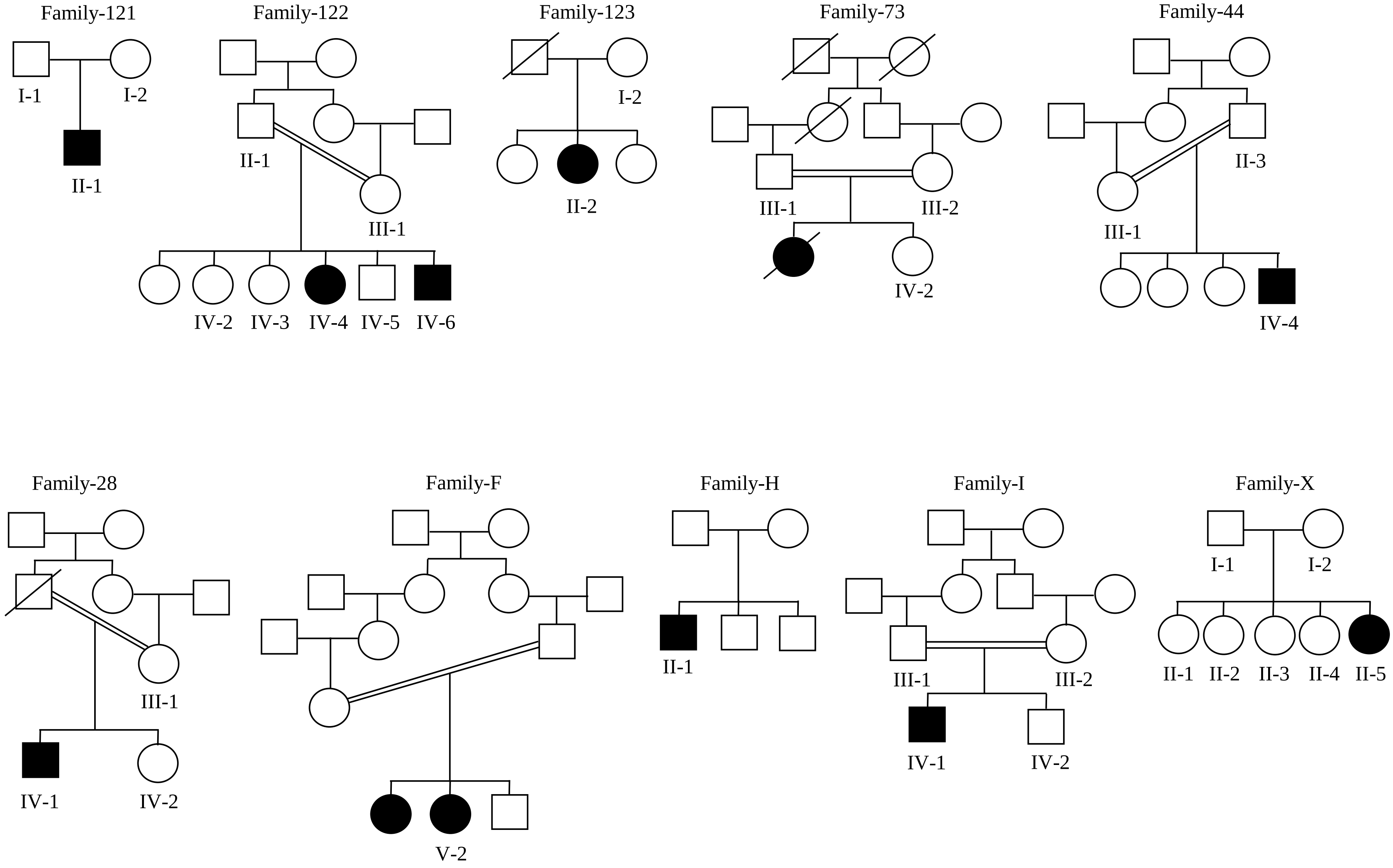
Family-121
I-1
I-2
II-1
Family-122
II-1
III-1
IV-2
IV-3
IV-4
IV-5
IV-6
Family-123
I-2
II-2
Family-73
III-1
III-2
IV-2
Family-44
II-3
III-1
IV-4
Family-28
III-1
IV-1
IV-2
Family-F
V-2
Family-H
II-1
Family-I
III-1
III-2
IV-1
IV-2
Family-X
I-1
I-2
II-1
II-2
II-3
II-4
II-5

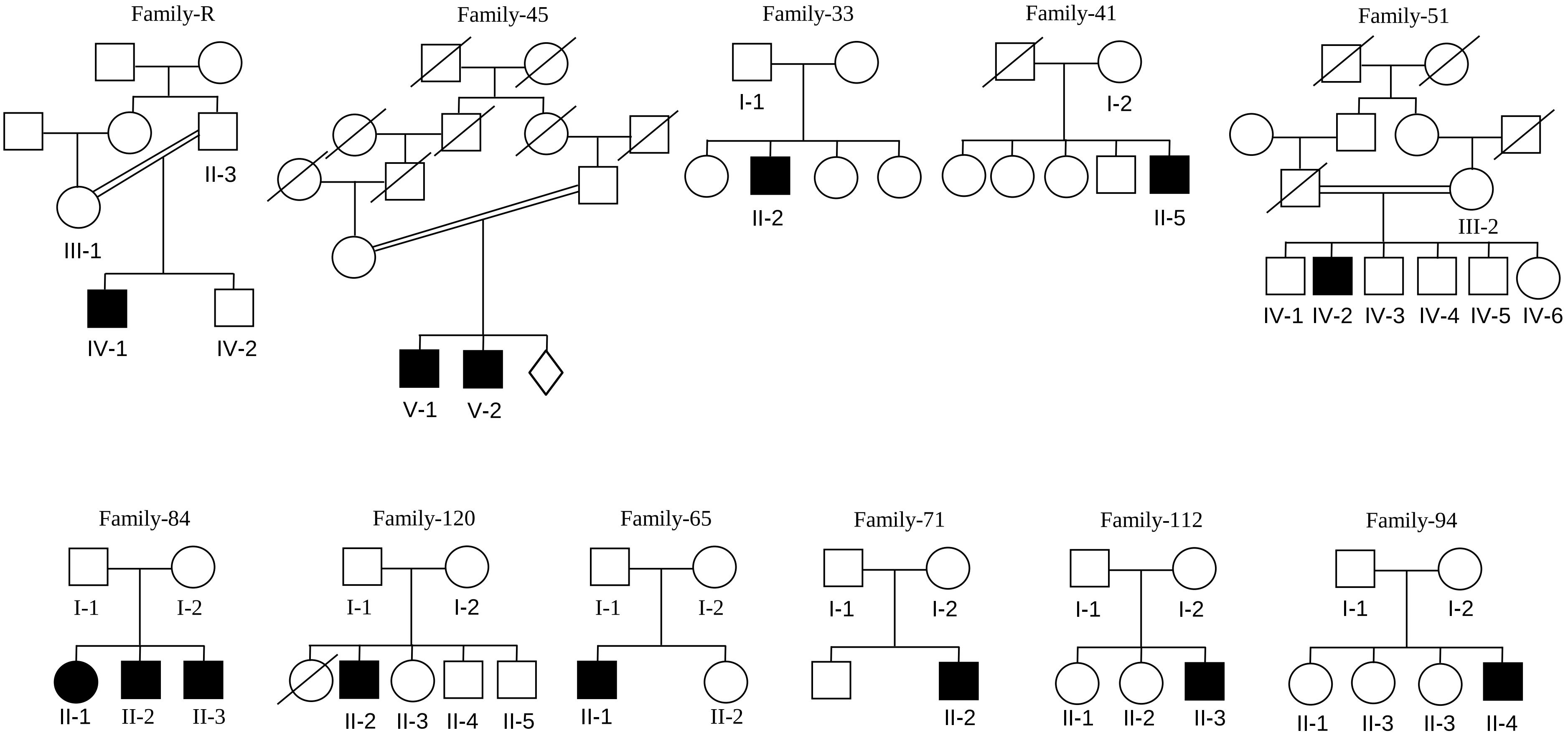
Family-R
II-3
III-1
IV-1
IV-2
Family-45
V-1
V-2
Family-33
I-1
II-2
Family-41
I-2
II-5
Family-51
III-2
IV-1
IV-2
IV-3
IV-4
IV-5
IV-6
Family-84
I-1
I-2
II-1
II-2
II-3
Family-120
I-1
I-2
II-2
II-3
II-4
II-5
Family-65
I-1
I-2
II-1
II-2
Family-71
I-1
I-2
II-2
Family-112
I-1
I-2
II-1
II-2
II-3
Family-94
I-1
I-2
II-1
II-3
II-3
II-4

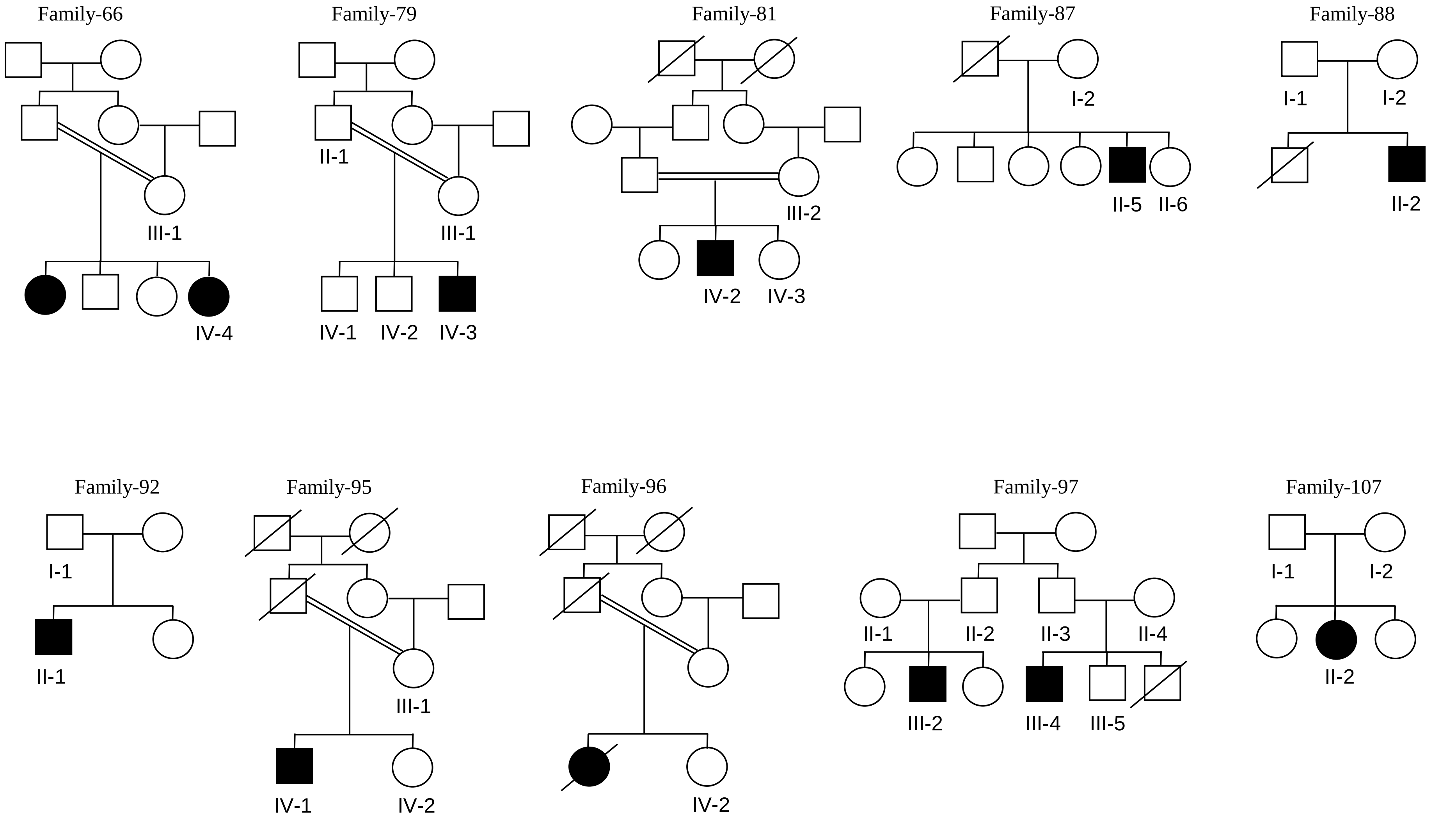

Fig A. Pedigrees of WD families. The individuals numbered in the pedigrees are the ones from whom the genomic DNA samples were isolated.

Chromosomal position (bp)	Marker	Family-B		Family-90	
52477914-52478251	D13S1325	3	4	3	4
52511362	rs9535795	G	G	G	G
52511606	rs2282057	C	C	C	C
52515354	rs1801249	T	T	T	T
52520471	rs1801247	G	G	G	G
52520504	c.2976C>T	C	C	C	C
52523808	rs732774	G	G	G	G
52524499	rs1061472	A	A	A	A
52524509	rs570594838	C	C	C	C
52524560	rs9526811	G	G	G	G
52532510	rs372979339	C	C	C	C
52532606	rs201213995	C	C	C	C
52542796	rs2147363	C	A	A	A
52544805	rs1801244	G	G	G	G
52548140	rs1801243	T	T	T	T
52549095	c.261C>T	C	C	C	C
53549182-52549183	**c.172_173insC (p.Ala58fs*19)**	-	**C**	**C**	**C**
52585548	rs2277448	A	C	C	C
52585615	c.-142T>C	T	T	T	T
53143966 - 53144249	D13S773	1	1	1	1
53175869 - 53176208	D13S1305	2	3	2	3

Fig B. The haplotype analysis to show the founder effect for the c.172_173insC mutation in 2 families. The disease haplotype is colored in grey, and corresponds to 632.8 Kb of DNA.

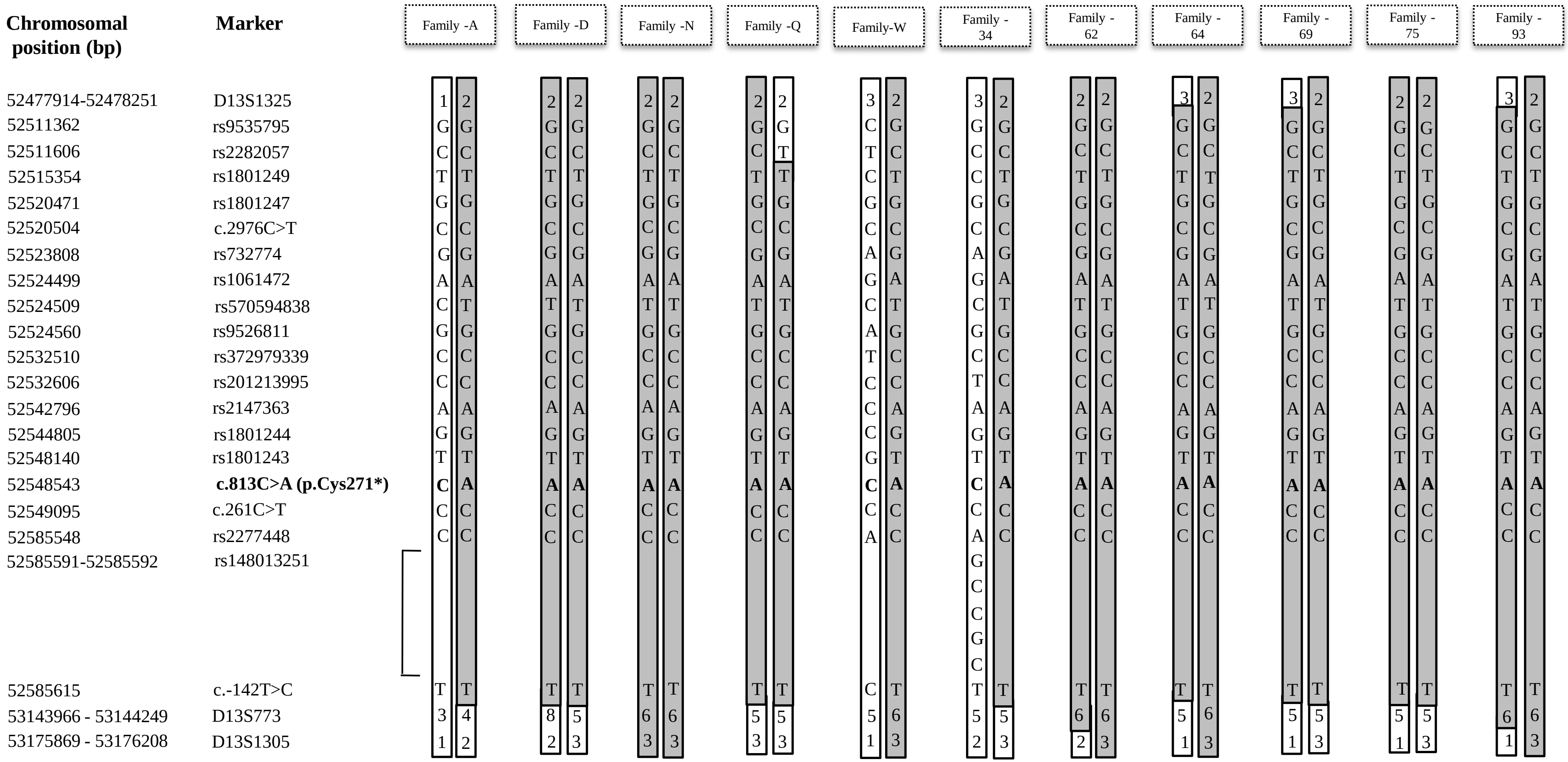

Fig C. The haplotype analysis to show the founder effect for the c.813C>A (p.Cys271*) mutation in 11 families. The disease haplotype is colored in grey, and corresponds to 70.2 Kb-698.2 Kb of DNA. Bracket denotes the insertion of 5'GCCGC3' nucleotides between the SNPs rs2277448 and c.-142T>C.

Chromosomal position (bp)	Marker	Family-L		Family-S		Family -42		Family-50		Family-53		Family-74		Family-82		Family-116		Family-419	
52477914-52478251	D13S1325	4	7	7	7	7	7	7	7	7	7	4	7	7	7	7	7	4	7
52511362	rs9535795	G	C	C	C	C	C	C	C	C	C	G	C	C	C	C	C	G	C
52511606	rs2282057	C	T	T	T	T	T	T	T	T	T	C	T	T	T	T	T	T	T
52515354	rs1801249	T	C	C	C	C	C	C	C	C	C	T	C	C	C	C	C	C	C
52520471	rs1801247	G	A	A	A	A	A	A	A	A	A	G	A	A	A	A	A	A	A
52520504	c.2976C>T	C	C	C	C	C	C	C	C	C	C	C	C	C	C	C	C	C	C
52523808	rs732774	G	A	A	A	A	A	A	A	A	A	G	A	A	A	A	A	A	A
52524499	rs1061472	A	G	G	G	G	G	G	G	G	G	A	G	G	G	G	G	G	G
52524509	rs570594838	C	C	C	C	C	C	C	C	C	C	C	C	C	C	C	C	C	C
52524560	rs9526811	G	G	G	G	G	G	G	G	G	G	G	G	G	G	G	G	G	G
52532510	rs372979339	C	C	C	C	C	C	C	C	C	C	C	C	C	C	C	C	C	C
52532606	rs201213995	C	C	C	C	C	C	C	C	C	C	C	C	C	C	C	C	C	C
52532671	**c.2131G>T (p.Gly711Trp)**	**G**	**T**	**T**	**T**	**T**	**T**	**T**	**T**	**T**	**T**	**G**	**T**	**T**	**T**	**T**	**T**	**T**	**T**
52542796	rs2147363	A	C	C	C	C	C	C	C	C	C	A	C	C	C	C	C	C	C
52544805	rs1801244	G	G	G	G	G	G	G	G	G	G	G	G	G	G	G	G	G	G
52548140	rs1801243	T	T	T	T	T	T	T	T	T	T	T	T	T	T	T	T	T	T
52549095	c.261C>T	C	C	C	C	C	C	C	C	C	C	C	C	C	C	C	C	C	C
52585548	rs2277448	C	A	A	A	A	A	A	A	A	A	C	A	A	A	A	A	A	A
52585615	c.-142T>C	T	T	T	T	T	T	T	T	T	T	T	T	T	T	T	T	T	T
53143966 - 53144249	D13S773	3	6	6	4	6	6	6	4	6	4	3	6	6	4	6	4	3	6
53175869 - 53176208	D13S1305	1	4	4	4	1	2	4	4	4	4	1	4	4	4	4	4	1	4

Fig D. The haplotype analysis to show the founder effect for the c.2131G>T (p.Gly711Trp) mutation in nine families. The disease haplotype is colored in grey, and corresponds to 74.2 Kb-698.2 Kb of DNA.

Chromosomal Position (bp)	Marker	Family-P		Family-85	
52477914-52478251	D13S1325	2	2	2	2
52511362	rs9535795	C	C	C	C
52511606	rs2282057	T	T	T	T
52515354	rs1801249	C	C	C	C
52520471	rs1801247	G	G	G	G
52520504	c.2976C>T	C	C	C	C
52523808	rs732774	A	A	A	A
52524499	rs1061472	G	G	G	G
52524509	rs570594838	C	C	C	C
52524560	rs9526811	A	A	A	A
52532469	**c.2333G>A (p.Arg778Gln)**	**A**	**A**	**A**	**A**
52532510	rs372979339	C	C	C	C
52532606	rs201213995	C	C	C	C
52542796	rs2147363	C	C	C	C
52544805	rs1801244	C	C	C	C
52548140	rs1801243	G	G	G	G
52549095	c.261C>T	C	C	C	C
52585548	rs2277448	A	A	A	A
52585591-52585592	rs148013251	G	G	G	G
		C	C	C	C
		C	C	C	C
		G	G	G	G
		C	C	C	C
52585615	c.-142T>C	T	T	T	T
53143966 - 53144249	D13S773	2	2	2	2
53175869 - 53176208	D13S1305	2	2	3	4

Fig E. The haplotype analysis to show the founder effect for the c.2333G>A (p.Arg778Gln) mutation in family-P and family-85. The disease haplotype is colored in grey, and corresponds to 666.3 Kb of DNA. Bracket denotes the insertion of 5'GCCGC3' nucleotides between the SNPs rs2277448 andc.-142T>C.

Chromosomal Position (bp)	Marker	Family-46		Family-118	
52477914-52478251	D13S1325	2	2	2	2
52511362	rs9535795	G	G	G	G
52511606	rs2282057	C	C	C	C
52515354	rs1801249	T	T	T	T
52520471	rs1801247	G	G	G	G
52520482	**c.2998G>A (p.Gly1000Arg)**	**A**	**A**	**A**	**A**
52520504	c.2976C>T	C	C	C	C
52523808	rs732774	G	G	G	G
52524499	rs1061472	A	A	A	A
52524509	rs570594838	C	C	C	C
52524560	rs9526811	G	G	G	G
52532510	rs372979339	C	C	C	C
52532606	rs201213995	C	C	C	C
52542796	rs2147363	A	A	C	C
52544805	rs1801244	G	G	G	G
52548140	rs1801243	T	T	T	T
52549095	c.261C>T	C	C	C	C
52585548	rs2277448	C	C	C	C
52585615	c.-142T>C	T	T	T	T
53143966 - 53144249	D13S773	3	3	3	3
53175869 - 53176208	D13S1305	2	4	2	4

Fig F. The haplotype analysis to show the founder effect for the c.2998G>A (p.Gly1000Arg) mutation in family-46 and family-118. The disease haplotype is colored in grey, and corresponds to 54.6 Kb of DNA.

Chromosomal Position (bp)	Marker	Family-36		Family-54		Family-83		Family-100	
52477914-52478251	D13S1325	1	1	1	1	1	1	1	1
52511362	rs9535795	G	G	G	G	G	G	G	G
52511606	rs2282057	C	C	C	C	C	C	C	C
52515354	rs1801249	T	T	T	T	T	T	T	T
52520471	rs1801247	G	G	G	G	G	G	G	G
5252042	**c.3008C>T (p.Ala1003Val)**	**T**	**T**	**T**	**T**	**T**	**T**	**T**	**T**
52520504	c.2976C>T	C	C	C	C	C	C	C	C
52523808	rs732774	G	G	G	G	G	G	G	G
52524499	rs1061472	A	A	A	A	A	A	A	A
52524509	rs570594838	C	C	C	C	C	C	C	C
52524560	rs9526811	G	G	G	G	G	G	G	G
52532510	rs372979339	C	C	C	C	C	C	C	C
52532606	rs201213995	C	C	C	C	C	C	C	C
52542796	rs2147363	A	A	A	A	A	A	A	A
52544805	rs1801244	G	G	G	G	G	G	G	G
52548140	rs1801243	T	T	T	T	T	T	T	T
52549095	c.261C>T	C	C	C	T	C	C	C	C
52585548	rs2277448	C	C	C	C	C	C	C	C
52585615	c.-142T>C	T	T	T	T	T	T	T	T
53143966 - 53144249	D13S773	1	1	1	1	1	1	1	1
53175869 - 53176208	D13S1305	1	4	1	4	1	4	1	4

Fig G. The haplotype analysis to show the founder effect for the c.3008C>T (p.Ala1003Val) mutation in four families. The disease haplotype is colored in grey, and corresponds to 70.2 Kb-698.2 Kb of DNA.

Chromosomal position (bp)	Marker	Family-V		Family-O		Family-38		Family-41B		Family-58		Family-78		Family-101	
52477914-52478251	D13S1325	3	2	3	2	3	2	3	2	5	5	3	2	3	2
52511362	rs9535795	G	G	G	G	C	G	G	G	G	G	G	G	G	G
52511606	rs2282057	C	C	C	C	T	C	C	C	C	C	C	C	C	C
52515354	rs1801249	T	T	T	T	C	T	T	T	T	T	C	T	T	T
52518306	**c.3182G>A (p.Gly1061Glu)**	**A**	**A**	**A**	**A**	**G**	**A**	**G**	**A**	**A**	**A**	**G**	**A**	**A**	**A**
52520471	rs1801247	G	G	G	G	G	G	G	G	G	G	G	G	G	G
52520504	c.2976C>T	C	C	C	C	C	C	C	C	C	C	C	C	C	C
52523808	rs732774	G	G	G	G	A	G	G	G	G	G	A	G	G	G
52524499	rs1061472	A	A	A	A	G	A	A	A	A	A	G	A	A	A
52524509	rs570594838	C	C	C	C	C	C	C	C	C	C	C	C	C	C
52524560	rs9526811	G	G	G	G	G	G	G	G	G	G	G	G	G	G
52532510	rs372979339	C	C	C	C	C	C	C	C	C	C	C	C	C	C
52532606	rs201213995	C	C	C	C	C	C	C	C	C	C	C	C	C	C
52542796	rs2147363	C	C	C	C	A	C	C	C	C	C	A	C	C	C
52544805	rs1801244	G	G	G	G	G	G	G	G	G	G	G	G	G	G
52548140	rs1801243	T	T	T	T	T	T	T	T	T	T	T	T	T	T
52549095	c.261C>T	C	C	C	C	C	C	C	C	C	C	C	C	C	C
52585548	rs2277448	A	A	A	A	A	A	C	A	A	A	A	A	A	A
52585615	c.-142T>C	T	T	T	T	T	T	T	T	T	T	T	T	T	T
53143966 - 53144249	D13S773	2	2	2	2	4	2	4	2	2	2	4	2	2	2
53175869 - 53176208	D13S1305	2	1	2	1	3	1	2	1	2	1	3	1	2	1

Fig H. The haplotype analysis to show the founder effect for the c.3182G>A (p.Gly1061Glu) mutation in seven families. The disease haplotype is colored in grey, and corresponds to 632.8 Kb-698.2 Kb of DNA.

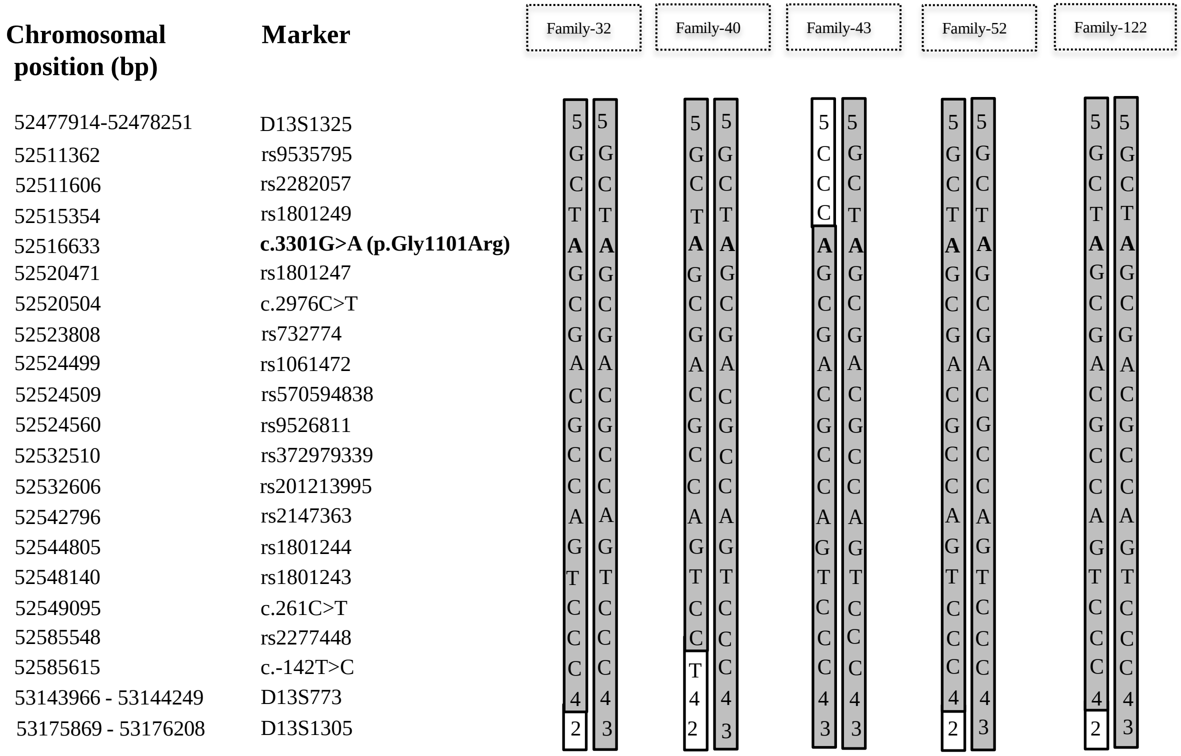

Fig I. The haplotype analysis to show the founder effect for the c.3301G>A (p.Gly1101Arg) mutation in five families. The disease haplotype is colored in grey, and corresponds to 107.6 Kb-698.2 Kb of DNA.

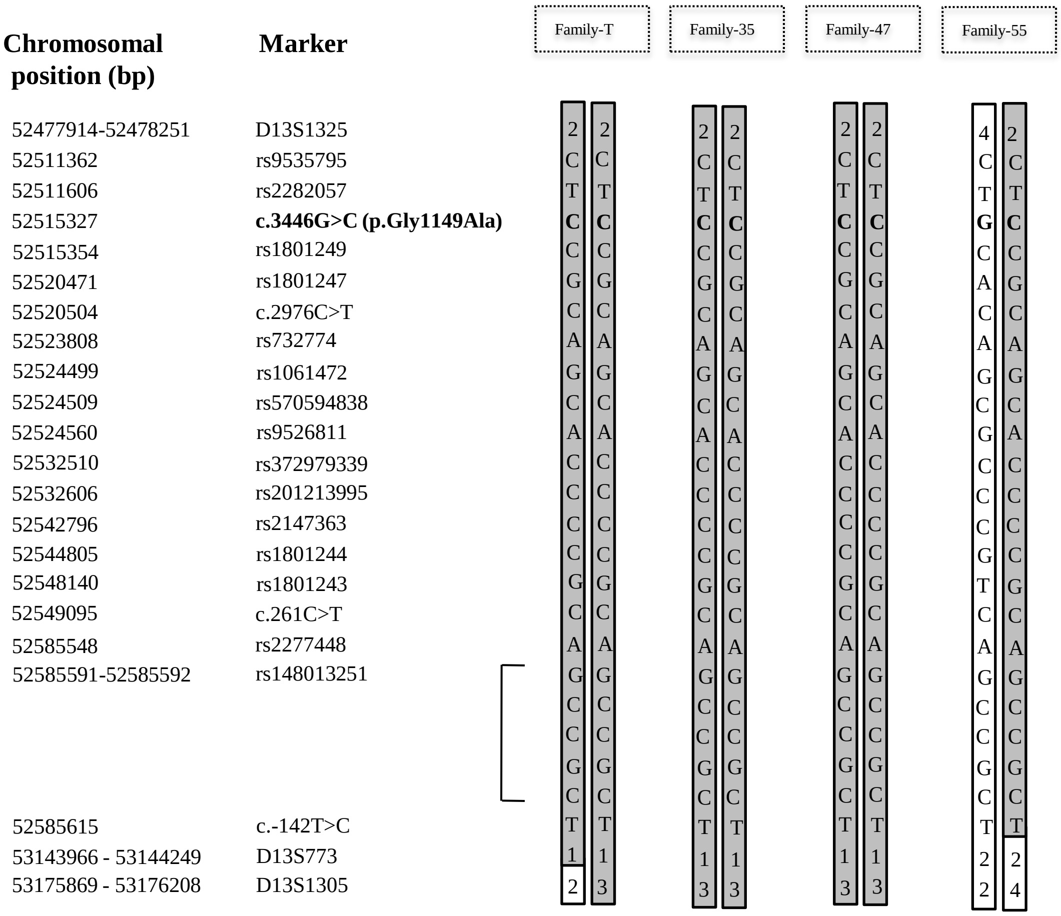

Fig J. The haplotype analysis to show the founder effect for the c.3446G>C (p.Gly1149Ala) mutation in four families. The disease haplotype is colored in grey, and corresponds to 107.7 Kb-698.2 Kb of DNA. Bracket denotes the insertion of 5’GCCGC3’ nucleotides between the SNPs rs2277448 and c.-142T>C.

Chromosomal position (bp)	Marker	Family-E		Family-G		Family-44		Family-59		Family-91	
52477914-52478251	D13S1325	3	2	5	2	3	2	2	2	3	2
52511362	rs9535795	G	G	G	G	G	G	G	G	G	G
52511606	rs2282057	T	C	T	C	T	C	C	C	C	C
52515241	**c.3532A>G (p.Thr1178Ala)**	**A**	**G**	**A**	**G**	**A**	**G**	**G**	**G**	**G**	**G**
52515354	rs1801249	C	T	C	T	C	T	T	T	T	T
52520471	rs1801247	G	G	G	G	G	G	G	G	G	G
52520504	c.2976C>T	C	T	C	T	C	T	T	T	T	T
52523808	rs732774	A	G	A	G	A	G	G	G	G	G
52524499	rs1061472	G	A	G	A	G	A	A	A	A	A
52524509	rs570594838	C	C	C	C	C	C	C	C	C	C
52524560	rs9526811	A	G	A	G	G	G	G	G	G	G
52532510	rs372979339	C	C	C	C	C	C	C	C	C	C
52532606	rs201213995	C	C	C	C	C	C	C	C	C	C
52542796	rs2147363	A	A	C	A	A	A	A	A	A	A
52544805	rs1801244	G	G	C	G	G	G	G	G	G	G
52548140	rs1801243	T	T	G	T	T	T	T	T	T	T
52549095	c.261C>T	C	C	C	C	C	C	C	C	C	C
52585548	rs2277448	A	C	A	C	A	C	C	C	C	C
52585615	c.-142T>C	T	T	T	T	T	T	T	T	T	T
53143966 - 53144249	D13S773	4	3	3	3	3	3	3	3	3	3
53175869 - 53176208	D13S1305	3	3	3	3	3	3	4	3	3	3

Fig K. The haplotype analysis to show the founder effect for the c.3532A>G (p.Thr1178Ala) mutation in five families. The disease haplotype is colored in grey, and corresponds to 664.8 Kb-698.2 Kb of DNA.

Chromosomal position (bp)	Marker	Family-B	Family-B (grey)	Family-90 (grey)	Family-90 (grey)
52477914-52478251	D13S1325	3	4	3	4
52511362	rs9535795	G	G	G	G
52511606	rs2282057	C	C	C	C
52511774	**c.3741C>G (p.His1247Gln)**	**C**	**G**	**G**	**G**
52515354	rs1801249	T	T	T	T
52520471	rs1801247	G	G	G	G
52520504	c.2976C>T	C	C	C	C
52523808	rs732774	G	G	G	G
52524499	rs1061472	A	A	A	A
52524509	rs570594838	C	C	C	C
52524560	rs9526811	G	G	G	G
52532510	rs372979339	C	C	C	C
52532606	rs201213995	C	C	C	C
52542796	rs2147363	C	A	A	A
52544805	rs1801244	G	G	G	G
52548140	rs1801243	T	T	T	T
52549095	c.261C>T	C	C	C	C
52585548	rs2277448	A	C	C	C
52585615	c.-142T>C	T	T	T	T
53143966 - 53144249	D13S773	1	1	1	1
53175869 - 53176208	D13S1305	2	3	2	3

Fig L. The haplotype analysis to show the founder effect for the c.3741C>G (p.His1247Gln) mutation in family-B and family-90. The disease haplotype is colored in grey, and corresponds to 698.2 Kb of DNA.

Chromosomal position (bp)	Marker	Family-L	Family-L	Family-63	Family-63	Family-89	Family-89
52477914-52478251	D13S1325	7	4	4	4	4	4
52511362	rs9535795	G	C	G	C	C	C
52511606	rs2282057	C	T	C	T	T	T
52511706	**c.3809A>G (p.Asp1270Ser)**	**A**	**G**	**A**	**G**	**G**	**G**
52515354	rs1801249	T	C	T	C	C	C
52520471	rs1801247	A	G	G	G	G	G
52520504	c.2976C>T	C	C	C	C	C	C
52523808	rs732774	G	A	A	A	A	A
52524499	rs1061472	A	G	A	A	G	G
52524509	rs570594838	C	C	C	C	C	C
52524560	rs9526811	G	G	G	A	A	A
52532510	rs372979339	C	C	C	C	C	C
52532606	rs201213995	C	C	T	C	C	C
52542796	rs2147363	A	C	A	A	C	A
52544805	rs1801244	G	G	C	G	C	C
52548140	rs1801243	T	T	T	G	G	G
52549095	c.261C>T	C	C	C	C	C	C
52585548	rs2277448	C	A	C	A	A	A
52585591-2585592	rs148013251				G C C G C	G C C G C	G C C G C
52585615	c.-142T>C	T	T	T	T	T	T
53143966 - 53144249	D13S773	3	6	1	1	1	1
53175869 - 53176208	D13S1305	1	4	4	5	2	2

Fig M. The haplotype analysis to show the founder effect for the c.3809A>G (p.Asp1270Ser) mutation in three families. The disease haplotype is colored in grey, and corresponds to 45.8 Kb of DNA. Bracket denotes the insertion of 5'GCCGC3' nucleotides between the SNPs rs2277448 and c.-142T>C.

Chromosomal position (bp)	Marker	Family-U	Family-U (grey)	Family-70	Family-70 (grey)
52477914-52478251	D13S1325	1	1	1	1
52511362	rs9535795	G	G	G	G
52511606	rs2282057	T	C	C	C
52511620	**c.3895C>T (p.Leu1299Phe)**	**C**	**T**	**C**	**T**
52515354	rs1801249	T	T	T	T
52520471	rs1801247	G	G	G	G
52520504	c.2976C>T	C	C	C	C
52523808	rs732774	G	G	G	G
52524499	rs1061472	A	A	A	A
52524509	rs570594838	C	C	T	C
52524560	rs9526811	G	G	G	G
52532510	rs372979339	C	C	C	C
52532606	rs201213995	C	C	C	C
52542796	rs2147363	C	C	A	C
52544805	rs1801244	G	G	G	G
52548140	rs1801243	T	T	T	T
52549095	c.261C>T	C	C	C	C
52585548	rs2277448	C	C	C	C
52585615	c.-142T>C	T	T	T	T
53143966 - 53144249	D13S773	1	1	1	1
53175869 - 53176208	D13S1305	4	1	3	1

Fig N. The haplotype analysis to show the founder effect for the c.3895C>T (p.Leu1299Phe) mutation in family-U and family-70. The disease haplotype is colored in grey, and corresponds to 698.2 Kb of DNA.

Chromosomal position (bp)	Marker	Family-K	Family-K	Family-Y	Family-Y	Family-57	Family-57
52477914-52478251	D13S1325	1	2	2	2	2	2
52511362	rs9535795	C	G	G	G	C	G
52511412	**c.4021G>A (p.Gly1341Ser)**	**A**	**A**	**G**	**A**	**G**	**A**
52511606	rs2282057	T	C	C	C	T	C
52515354	rs1801249	C	T	T	T	C	T
52520471	rs1801247	G	G	G	G	G	G
52520504	c.2976C>T	C	C	C	C	C	C
52523808	rs732774	A	G	G	G	A	G
52524499	rs1061472	G	A	A	A	G	A
52524509	rs570594838	C	C	C	C	C	C
52524560	rs9526811	A	G	G	G	G	G
52532510	rs372979339	C	C	C	C	C	C
52532606	rs201213995	C	C	C	C	C	C
52542796	rs2147363	C	A	A	A	A	A
52544805	rs1801244	C	G	G	G	G	G
52548140	rs1801243	G	T	T	T	T	T
52549095	c.261C>T	C	C	C	C	C	C
52585548	rs2277448	A	A	A	A	A	A
52585591-52585592	rs148013251	G	G	G	G		
		C	C	C	C		
		C	C	C	C		
		G	G	G	G		
		C	C	C	C		
52585615	c.-142T>C	T	T	T	T	T	T
53143966 - 53144249	D13S773	2	4	1	3	5	3
53175869 - 53176208	D13S1305	3	3	2	3	2	3

Fig O. The haplotype analysis to show the founder effect for the c.4021G>A (p.Gly1341Ser) mutation in three families. The disease haplotype is colored in grey, and corresponds to 107.6 Kb of DNA. Bracket denotes the insertion of 5’GCCGC3’ nucleotides between the SNPs rs2277448 and c.-142T>C.

Table A. Clinical features of Wilson disease patients.

Sl.#	Family name	Affected individual	Age(yr)/Sex	Age at onset (yr)	Serum copper (Normal range: 75-160mcg/dL)	Serum ceruloplasmin (Normal range:15-35mg/dL)	24 hr Urinary protein (Normal range: 20-40 mcg/24hrs)	KF ring	Jaundice (history)	Hepatomegaly
1	Family-A	II-2	22/F	16	36	3	210	+	-	+
2	Family-D	II-1	29/M	6	81	1	90	+	+	+
3	Family-W	II-1	16/F	13.5	37	1	69	+	+	-
4	Family-N	IV-3	19/F	14	28	2	90	+	-	-
5	Family-Q	IV-3	14/M	11	10	4	72	+	+	+
6	Family-34	IV-6	30/F	20	80	4.5	354	+	-	NA
7	Family-62	IV-3	11/F	9	16	2	120	+	+	+
8	Family-64	II-2	14/M	14	NA	NA	NA	+	+	+
9	Family-69	II-1	11/F	9	16	5	69	+	+	+
10	Family-75	IV-1	17/F	12	30	2	150	-	-	-
11	Family-93	IV-3	38/F	9	38	2.7	162	+	-	-
12	Family-419	IV-2	18/F	11	21	4	726	+	-	-
13	Family-L	II-1	19/M	11	42	4	520	+	-	+
14	Family-S	III-3	36/M	12	31	4	258	+	+	-

15	Family-82	IV-1	3/M	8	22	4	510	+	+	+
16	Family-116	V-4	8/F	Asymptomatic	14	10	200	+	+	+
17	Family-42	IV-6	21/M	24	14	2	116	+	NA	NA
18	Family-50	II-5	19/M	9	30	3	336	+	+	+
19	Family-53	IV-2	18/F	8	NA	7	560	+	+	NA
20	Family-74	II-2	15/M	11	42	4	95	+	-	-
21	Family-V	IV-2	17/M	13	22	4	519	+	-	-
22	Family-O	II-1	14/F	11	14	4	613	+	-	-
23	Family-38	II-2	12/M	12	74	8	45	+	NA	NA
24	Family-58	II-2	30/M	12	27	4	451	+	NA	NA
25	Family-78	II-1	28/M	13	13	0.6	120	+	NA	NA
26	Family-41B	II-1	25/M	23	40	3	897	+	NA	NA
27	Family-K	IV-1	21/F	9	31	10	600	+	-	-
28	Family-Y	II-1	33/M	8	20	4	810	+	+	-
29	Family-57	II-1	16/M	14	NA	NA	NA	+	NA	NA
30	Family-E	II-2	28/M	23	56	5	586	+	-	-
31	Family-G	II-1	33/M	22	28	4	224	+	-	-
32	Family-59	IV-2	9/F	7	62	7	513	+	NA	NA

33	Family-59	IV-3	2/M	NA	NA	NA	NA	NA	NA	NA
34	Family-91	IV-5	35/M	29	55	4	27	+	NA	NA
35	Family-P	II-1	18/F	15	51	5	352	+	+	-
36	Family-85	IV-2	35/M	19	16	8	578	+	-	-
37	Family-T	IV-6	23/M	11	18	2	343	+	-	-
38	Family-35	II-5	19/M	16	50	16	240	+	NA	-
39	Family-35	II-6	14/F	6	14	1	36	+	NA	-
40	Family-47	IV-6	8/M	8	31	5	312	+	NA	NA
41	Family-55	II-1	13/F	11	60	8.6	6.2	+	NA	NA
42	Family-U	IV-2	36/M	28	43	4	NA	+	+	-
43	Family-70	II-6	35/F	31	20	2	200	+	-	-
44	Family-83	IV-1	23/F	17	24	6	195	+	+	-
45	Family-36	IV-3	30/M	16	11	4	299	+	-	-
46	Family-54	IV-4	28/F	24	50	18	62	+	-	-
47	Family-506	II-1	14/M	10	20	4	192	+	-	-
48	Family-128	IV-5	31/F	14	NA	6	52	+	-	+
49	Family-31	II-3	14/M	14	28	5	99	+	+	-
50	Family-31	II-4	12/M	12	36	5	NA	+	-	-

51	Family-B	II-3	23/M	15	50	2	NA	+	-	-
52	Family-C	II-3	32/M	10	51	3	515	+	-	-
53	Family-M	II-1	12/M	7	30	4	66	+	+	-
54	Family-80	IV-1	19/F	11	NA	NA	NA	+	-	-
55	Family-32	V-5	12/M	10	16	4	444	+	-	-
56	Family-40	II-2	16/M	9	13	5	247	+	+	-
57	Family-43	IV-1	23/M	10	64	4	543	+	NA	NA
58	Family-52	IV-2	11/M	9	25	5	102	+	NA	+
59	Family-46	II-1	46/M	39	42	9	884	+	NA	NA
60	Family-49	IV-1	19/M	17	18	2	300	+	+	-
61	Family-56	II-2	33/M	10	52	9	630	+	-	NA
62	Family-60	V-1	11/F	10	28	6	72	+	-	-
63	Family-63	II-3	31/F	19	24	3	220	+	-	-
64	Family-89	II-5	24/F	9	40	3	915	+	-	-
65	Family-67	II-1	29/M	21	56	6	860	+	NA	NA
66	Family-72	IV-1	21/F	20	NA	NA	NA	+	-	NA
67	Family-72	IV-2	20/F	18	26	2	385	+	-	-
68	Family-72	IV-3	25/M	24	40	4	190	+	-	-
69	Family-76	IV-2	24/M	11	27	5	175	+	+	-

70	Family-77	II-2	14/M	10	NA	NA	NA	+	NA	NA
71	Family-90	IV-1	14/M	12.9	30	5	130	+	+	NA
72	Family-86	II-2	9/F	9	38	6	898	+	-	-
73	Family-98	II-2	14/M	10	67	3.5	209	+	NA	-
74	Family-100	IV-3	15/M	16	46	7	240	+	NA	NA
75	Family-101	IV-2	NA /M	12	42	5	445	+	NA	NA
76	Family-101	IV-3	NA /M	12	30	5	380	+	+	+
77	Family-118	IV-4	55/F	30	86	8	2280	+	NA	NA
78	Family-121	II-1	14/F	12	30	5	296	+	-	-
79	Family-122	IV-2	11/F	9	92	6	11	+	NA	NA
80	Family-122	IV-4	8/F	8	44	4	647	+	NA	NA
81	Family-123	II-2	15/F	15	28	6	318	+	-	-
82	Family-73	IV-2	22/F	12.6	76	26	279	+	-	-
83	Family-44	IV-4	NA/M	NA	NA	NA	NA	NA	NA	NA
84	Family-28	IV-I	41/M	10	27	3	175	+	+	-
85	Family-F	V-2	31/F	18	24	1	515	+	-	-
86	Family-H	II-1	27/M	20	40	2	689	+	-	-
87	Family-I	IV-I	38/M	17	27	3	170	+	-	-
88	Family-X	II-5	15/F	11	75	2	91	+	+	-

89	Family-R	IV-I	27/M	19	20	4	188	+	-	-
90	Family-45	V-1	17/M	asymptomatic	3	8	1040	-	+	-
91	Family-45	V-2	14/M	14	124	9	1215	+	-	-
92	Family-33	II-2	30/M	23	76	20	504	-	+	-
93	Family-41	II-5	31/M	12	42	6	135	+	-	-
94	Family-51	IV-2	25/M	20	5	13	347	+	-	-
95	Family-84	II-1	15/F	7	113	28	514	+	+	-
96	Family-84	II-2	18/M	16	NA	NA	NA	+	-	-
97	Family-84	II-3	17/M	17	NA	22	639	+	-	-
98	Family-120	II-2	24/M	12	37	6	291	+	-	-
99	Family-65	II-1	13/M	12	1	19	217	+	-	-
100	Family-71	II-2	11/M	11	57	13	349	-	-	-
101	Family-112	II-3	15/M	12	36	5	507	+	-	-
102	Family-94	II-4	32/M	15	12	2	380	+	-	-
103	Family-66	IV-4	25/F	24	68	3	638	+	-	-
104	Family-79	IV-3	27/M	17	103	18	150	+	-	-
105	Family-81	IV-2	20/M	13	32	4	NA	+	-	-
106	Family-87	II-6	18/F	15	44	3	NA	+	-	NA
107	Family-88	II-2	19/M	14	6	2	110	+	-	-
108	Family-92	II-1	32/M	11	37	5	312	+	-	-

109	Family-95	IV-1	25/M	24	34	5	145	+	-	-
110	Family-96	IV-2	26/F	25		0	370	+	-	
111	Family-97	III-2	25/M	12	76	14	332	+	-	-
112	Family-97	III-4	35/M	30	54	4	348	+	-	-
113	Family-107	II-2	22/F	13	76	26	279	+	-	-

Sl.#	Family name	Affected individual	Splenomegaly	Dysphagia	Dysarthria	Tremor	Dystonia	Writing difficulty	Chorea	Athetosis
1	Family-A	II-2	-	+	+	+	+	+	-	-
2	Family-D	II-1	+	-	+	-	+	+	-	-
3	Family-W	II-1	+	+	+	-	+	+	-	-
4	Family-N	IV-3	+	-	+	-	+	+	-	-
5	Family-Q	IV-3	+	-	+	-	+	+	-	-
6	Family-34	IV-6	+	+	+	+	+	+	NA	NA
7	Family-62	IV-3	+	+	+	+	NA	+	-	-
8	Family-64	II-2	+	+	+	-	+	+	-	-
9	Family-69	II-1	+	+	+	-	+	+	-	-
10	Family-75	IV-1	+	+	+	-	+	+	-	-
11	Family-93	IV-3	-	+	+	+	+	+	+	+
12	Family-419	IV-2	-	-	+	+	-	-	-	-

13	Family-L	II-1	+	-	+	+	+	+	+	-
14	Family-S	III-3	+	-	-	+	+	+	-	-
15	Family-82	IV-1	-	-	-	-	-	-	-	-
16	Family-116	V-4	+	-	-	-	-	-	-	-
17	Family-42	IV-6	NA	-	-	-	-	-	-	-
18	Family-50	II-5	+	+	+	-	+	+	-	-
19	Family-53	IV-2	NA	+	+	-	-	+	+	-
20	Family-74	II-2	+	-	+	-	+	+	-	-
21	Family-V	IV-2	-	+	+	-	-	+	-	-
22	Family-O	II-1	-	+	+	-	+	+	+	-
23	Family-38	II-2	NA	+	+	+	+	+	-	-
24	Family-58	II-2	NA	+	+	+	+	+	-	-
25	Family-78	II-1	NA	-	+	+	-	+	+	+
26	Family-41B	II-1	NA	-	+	+	-	-	-	-
27	Family-K	IV-1	-	-	+	+	+	+	-	-
28	Family-Y	II-1	+	+	+	-	+	+	-	-
29	Family-57	II-1	NA	-	+	+	+	+	-	-
30	Family-E	II-2	-	-	+	+	+	+	-	-
31	Family-G	II-1	-	-	+	-	-	-	-	-
32	Family-59	IV-2	NA	-	-	-	-	-	-	-

33	Family-59	IV-3	NA	NA	NA	NA	NA	NA	NA	NA
34	Family-91	IV-5	NA	-	+	+	-	-	-	-
35	Family-P	II-1	-	-	+	-	-	+	-	-
36	Family-85	IV-2	-	-	+	+	-	-	-	-
37	Family-T	IV-6	-	+	+	-	+	+	-	-
38	Family-35	II-5	-	+	+	+	+	+	-	-
39	Family-35	II-6	-	+	+	+	+	+	-	-
40	Family-47	IV-6	NA	+	+	-	-	+	-	-
41	Family-55	II-1	NA	+	-	+	+	+	-	-
42	Family-U	IV-2	-	+	+	+	-	+	-	-
43	Family-70	II-6	-	+	+	-	-	+	-	-
44	Family-83	IV-1	-	-	-	+	-	-	-	-
45	Family-36	IV-3	-	-	-	-	-	+	-	-
46	Family-54	IV-4	-	+	+	-	+	+	-	-
47	Family-506	II-1	-	+	+	-	+	+	+	+
48	Family-128	IV-5	+	-	+	+	-	+	-	-
49	Family-31	II-3	-	+	+	-	+	+	-	-
50	Family-31	II-4	-	+	+	-	+	+	-	-
51	Family-B	II-3	+	-	+	-	+	-	-	-
52	Family-C	II-3	+	-	+	-	+	+	-	-

53	Family-M	II-1	-	+	+	-	+	+	-	-
54	Family-80	IV-1	-	-	+	-	+	+	-	-
55	Family-32	V-5	-	-	+	-	-	-	-	-
56	Family-40	II-2	-	NA	NA	+	+	+	+	+
57	Family-43	IV-1	NA	+	+	-	+	-	+	-
58	Family-52	IV-2	-	+	+	+	+	+	-	-
59	Family-46	II-1	NA	-	-	+	-	+	-	-
60	Family-49	IV-1	-	+	+	+	+	+	+	-
61	Family-56	II-2	-	-	+	-	-	-	-	-
62	Family-60	V-1	-	-	+	-	+	+	+	-
63	Family-63	II-3	-	-	-	-	-	-	-	-
64	Family-89	II-5	-	+	+	-	-	+	-	-
65	Family-67	II-1	NA	-	-	-	-	+	-	-
66	Family-72	IV-1	NA	NA	NA	NA	NA	NA	NA	NA
67	Family-72	IV-2	-	-	+	-	+	+	-	-
68	Family-72	IV-3	-	+	+	-	-	+	+	+
69	Family-76	IV-2	-	-	+	+	-	+	-	-
70	Family-77	II-2	NA	NA	NA	NA	NA	NA	NA	NA
71	Family-90	IV-1	NA	+	+	+	+	+	-	-
72	Family-86	II-2	NA	+	+	-	+	+	-	-

73	Family-98	II-2	+	-	+	-	+	+	+	-
74	Family-100	IV-3	NA	-	-	+	-	-	+	-
75	Family-101	IV-2	NA	-	+	+	+	+	-	-
76	Family-101	IV-3	-	-	+	+	+	+	-	-
77	Family-118	IV-4	NA	-	+	+	-	+	-	-
78	Family-121	II-1	NA	+	+	+	+	+	-	-
79	Family-122	IV-2	NA	+	+	+	+	+	-	-
80	Family-122	IV-4	NA	-	-	-	-	-	-	-
81	Family-123	II-2	-	+	+	+	+	+	-	-
82	Family-73	IV-2	-	-	+	+	-	+	-	-
83	Family-44	IV-4	NA	NA	NA	NA	NA	NA	NA	NA
84	Family-28	IV-1	-	+	+	-	+	+	-	-
85	Family-F	V-2	-	-	-	+	+	+	-	-
86	Family-H	II-1	-	-	-	+	-	-	-	-
87	Family-I	IV-I	-	-	+	+	-	+	-	-
88	Family-X	II-5	+	-	+	-	+	+	-	-
89	Family-R	IV-I	-	+	+	-	+	+	-	-
90	Family-45	V-1	-	-	-	-	-	-	-	-
91	Family-45	V-2	+	-	+	+	+	+	-	-
92	Family-33	II-2	-	+	+	+	+	+	-	-

93	Family-41	II-5	-	+	+	+	+	+	-	-
94	Family-51	IV-2	-	+	+	-	-	+	-	-
95	Family-84	II-1	+	-	-	-	-	-	-	-
96	Family-84	II-2	-	-	-	-	-	-	-	-
97	Family-84	II-3	-	-	-	-	-	-	-	-
98	Family-120	II-2	+	+	+	+	+	+	-	-
99	Family-65	II-1	-	-	+	-	+	+	-	-
100	Family-71	II-2	-	-	-	-	-	-	-	-
101	Family-112	II-3	+	-	+	+	+	+	-	-
102	Family-94	II-4	-	-	+	-	-	-	-	-
103	Family-66	IV-4	-	-	+	+	+	-	-	-
104	Family-79	IV-3	-	+	+	+	-	+	-	-
105	Family-81	IV-2	-	-	+	+	+	+	-	-
106	Family-87	II-6	-	-	+	+	+	+	-	-
107	Family-88	II-2	-	-	+	-	-	+	-	-
108	Family-92	II-1	-	-	+	+	+	+	-	-
109	Family-95	IV-1	+	+	+	+	-	+	-	-
110	Family-96	IV-2	-	-	-	-	-	+	-	-
111	Family-97	III-2	+	-	+	+	+	+	-	-
112	Family-97	III-4	-	-	-	+	-	-	-	-

113	Family-107	II-2	-	-	+	+	-	+	-	-

Sl.#	Family name	Affected individual	Parkinsonism	Rigidity	Bradykinesia	Cerebral atrophy	Cerebellar atrophy	Brain stem atrophy	Gaint panda sign
1	Family-A	II-2	+	NA	+	-	-	-	-
2	Family-D	II-1	+	+	+	-	-	-	-
3	Family-W	II-1	+	+	+	-	-	-	-
4	Family-N	IV-3	+	+	+	-	-	-	-
5	Family-Q	IV-3	+	+	+	-	-	-	-
6	Family-34	IV-6	NA	NA	NA	-	+	-	-
7	Family-62	IV-3	-	+	+	-	-	-	-
8	Family-64	II-2	NA	+	+	-	-	-	-
9	Family-69	II-1	NA	+	+	-	-	-	-
10	Family-75	IV-1	NA	+	+	-	-	-	-
11	Family-93	IV-3	NA	-	+	-	-	+	-
12	Family-419	IV-2	-	-	-	+	-	-	-
13	Family-L	II-1	+	+	-	-	-	-	+
14	Family-S	III-3	+	+	+	-	-	-	-
15	Family-82	IV-1	-	-	-	+	+	+	-

16	Family-116	V-4	-	-	-	-	-	-	-
17	Family-42	IV-6	-	-	-	-	-	-	-
18	Family-50	II-5	NA	+	+	-	-	-	-
19	Family-53	IV-2	NA	+	+	-	-	-	-
20	Family-74	II-2	NA	+	+	-	-	-	-
21	Family-V	IV-2	+	+	+	-	-	-	+
22	Family-O	II-1	+	+	+	-	-	-	-
23	Family-38	II-2	+	-	+	-	-	-	-
24	Family-58	II-2	+	+	+	-	-	-	-
25	Family-78	II-1	-	-	-	-	-	-	-
26	Family-41B	II-1	-	+	+	-	-	-	-
27	Family-K	IV-1	-	-	+	-	-	-	-
28	Family-Y	II-1	+	+	+	-	-	-	-
29	Family-57	II-1	+	+	+	-	-	-	-
30	Family-E	II-2	+	+	+	+	+	-	+
31	Family-G	II-1	-	-	-	-	-	-	-
32	Family-59	IV-2	-	-	-	-	-	-	-
33	Family-59	IV-3	NA	NA	NA	-	-	-	-
34	Family-91	IV-5	-	-	-	-	-	-	+

35	Family-P	II-1	-	-	+	-	-	-	+
36	Family-85	IV-2	-	-	-	-	-	-	+
37	Family-T	IV-6	+	+	+	-	-	-	+
38	Family-35	II-5	-	-	-	-	-	-	-
39	Family-35	II-6	-	-	-	-	-	-	-
40	Family-47	IV-6	-	-	-	-	-	-	-
41	Family-55	II-1	-	-	-	-	-	-	-
42	Family-U	IV-2	+	+	+	+	+	-	-
43	Family-70	II-6	+	+	+	-	-	-	-
44	Family-83	IV-1	-	-	-	-	-	-	-
45	Family-36	IV-3	+	+	+	-	-	-	-
46	Family-54	IV-4	-	-	-	-	-	-	-
47	Family-506	II-1	+	+	+	+	-	-	-
48	Family-128	IV-5	-	-	-	-	-	+	-
49	Family-31	II-3	+	+	+	-	-	-	+
50	Family-31	II-4	+	+	-	-	-	-	-
51	Family-B	II-3	+	+	+	NA	NA	NA	NA
52	Family-C	II-3	+	+	+	-	-	-	-
53	Family-M	II-1	+	+	+	-	-	-	-
54	Family-80	IV-1	+	+	+	-	-	-	-

55	Family-32	V-5	-	-	-	-	-	-	-
56	Family-40	II-2	+	+	+	-	-	-	-
57	Family-43	IV-1	-	-	-	-	-	-	-
58	Family-52	IV-2	+	+	+	-	-	-	-
59	Family-46	II-1	-	-	-	+	-	+	-
60	Family -49	IV-1	+	+	+	-	-	-	-
61	Family-56	II-2	-	-	-	-	-	-	-
62	Family-60	V-1	-	-	+	-	-	-	-
63	Family-63	II-3	-	+	-	-	-	-	-
64	Family-89	II-5	-	-	+	-	-	-	-
65	Family-67	II-1	-	-	-	NA	NA	NA	NA
66	Family-72	IV-1	NA	NA	NA	NA	-	-	-
67	Family-72	IV-2	+	+	+	-	-	-	-
68	Family-72	IV-3	NA	-	+	-	-	-	-
69	Family-76	IV-2	-	-	-	-	-	-	-
70	Family-77	II-2	NA	NA	NA	-	-	-	-
71	Family-90	IV-1	+	+	+	-	-	+	-

72	Family-86	II-2	+	+	+	-	-	-	-
73	Family-98	II-2	+	-	+	NA	-	-	-
74	Family-100	IV-3	-	-	-	NA	-	-	-
75	Family-101	IV-2	-	+	+	NA	-	-	-
76	Family-101	IV-3	-	-	-	NA	-	-	-
77	Family-118	IV-4	-	-	-	NA	-	-	-
78	Family-121	II-1	-	-	-	NA	-	-	-
79	Family-122	IV-2	-	+	+	NA	-	-	-
80	Family-122	IV-4	-	-	-	NA	-	-	+
81	Family-123	II-2	-	-	-	NA	-	-	-
82	Family-73	IV-2	-	-	-	NA	-	-	-
83	Family-44	IV-4	NA	NA	NA	NA	NA	NA	NA
84	Family-28	IV-I	+	+	+	-	-	-	-
85	Family-F	V-2	+	+	+	-	-	-	+
86	Family-H	II-1	-	-	-	NA	NA	NA	NA
87	Family-I	IV-I	+	-	+	-	-	-	+
88	Family-X	II-5	-	+	+	+	+	-	+
89	Family-R	IV-I	-	+	+	-	-	-	-
90	Family-45	V-1	-	-	-	-	-	-	-

91	Family-45	V-2	-	+	+	-	-	-	-
92	Family-33	II-2	-	-	-	-	-	-	+
93	Family-41	II-5	+	+	+	-	+	+	-
94	Family-51	IV-2	-	+	+	-	-	-	-
95	Family-84	II-1	-	-	-	-	-	-	-
96	Family-84	II-2	-	-	-	-	-	-	-
97	Family-84	II-3	-	-	-	-	-	-	-
98	Family-120	II-2	-	+	+	+	+	+	-
99	Family-65	II-1	-	-	-	-	-	-	-
100	Family-71	II-2	-	-	-	-	-	+	-
101	Family-112	II-3	+	+	+	-	-	-	+
102	Family-94	II-4	+	+	+	-	+	+	-
103	Family-66	IV-4	-	-	-	-	-	-	-
104	Family-79	IV-3	-	-	-	-	-	-	-
105	Family-81	IV-2	-	-	-	NA	NA	NA	NA
106	Family-87	II-6	-	+	+	-	+	+	+
107	Family-88	II-2	-	-	+	+	+	+	-
108	Family-92	II-1	+	+	+	+	+	+	-
109	Family-95	IV-1	+	+	+	+	-	-	+

110	Family-96	IV-2	-	-	-	+	-	-	-
111	Family-97	III-2	+	+	+	+	+	+	-
112	Family-97	III-4	-	-	-	+	+	+	-
113	Family-107	II-2	-	-	-	+	-	-	+

Abbreviations: +, present; -, absent; and, NA, not analysed.

Table B. PCR primers used for the mutation analysis of the *ATP7B* gene.

Sl.#	Exon	Primer sequence (5'→3')	T_m (°C)	Amplicon size (bp)
1	1	F: GACGGCGGCGCGCAACTTTGAATC<br>R: TCTGGCTCGGCCTTCCCTGCGCA	64	396
2	2	AF: AGTGGCATTGTTTTCCATTTTCTCAG<br>AR: CTTTCCTTCTGCAATGCTGGCCTC	64	435
3	2	BF: AGCATGAAGGTTTCCCTGGAACAAG<br>BR: CTTCCTTGGTGCCCCAAGGTCTC	64	500
4	2	CF: TGATATTGAGCGGTTACAAAGCACTA<br>CR: AGAACTGGAAGACCTGTGATCTGTC	64	364
5	2	DF: GCTATCGAGGCACTTCCACCTGG<br>DR: GGGAGCAGGGCTCACCTATACCA	64	440
6	3	F: GGTGGGAGCCGGGACAATGAACC<br>R: GTTATCAGGGCTACTGATAAACACAG	64	469
7	4	F: CTCATGTGCAGCCTCATTTTCAAAC<br>R: CCAAGATGGGGAAATTTACTAATCAC	62	410
8	5	F: CTTGGCTGCCTGTTACCTAGACTC<br>R: CCATTCACTGATATCCTCCCTCAGA	64	458
9	6	F: CAGAAAACCCACAAAGTCTACTGAGG<br>R: AAAGGCAGCTAATCCAGGAGGAAGG	64	335
10	7	F: CAAAGGCAGGTCTTAAACTGTGTCC;<br>R: GCTAAAGCACTATGTTTGCGCTTAGC	64	367
11	8	F: GTACTGTCACGACTGTGCACAAAGC<br>R: CCTATTTCTTTAAGTCTGTCTCTATGC	62	476
12	9	F: CATGTGTGGTGGATAGCAAGTAACG<br>R: CTTGCTTTCGTAGCTGGATTGAGAG	62	414
13	10	F: CAAATGAATACTGTTGCTCAGTATAAGC<br>R: TGGCATGGCTTCTCCTAGACGTAGG	60	372
14	11	F: TTCATGTTCCCTCAGGAGGATATCA<br>R: TCAGCCAGCTGCTGAATGGGTGC	64	484
15	12	F: AAGAGGCTCAGATGTCAAAGGTAATGA<br>R: AGAAACCTGCAGAAGGAGAGTGACTG	64	554

16	13	F: AGTGCCCCCCTGAAATGTCCTTATG<br><br>R: GTTCCATTATATGACTGGTGGCTACTC	64	471
17	14	F: GTTGAACCCTGAGATTGAACGACAG<br><br>R: ACTGCCCGTACTCCCCAAGACCG	64	655
18	15	F: TCTCAGTTCCCGCTTTCCGCTGCTC<br><br>R: CAGAGGCAATCACTGCTGGGCGTG	64	383
19	16	F: GGATGCTGTCACAAGAGGTGCTTACA<br><br>R: TGGAAAACAGGCCTGAAATTAAGAGAG	64	423
20	17	F: ATTCCTTGGGGAGCCACTGCGAAGA<br><br>R: ACAGCTCAGTGCTGGGCCAACTGG	64	460
21	18	F: CAAACCTGCAGGGTGTGGTTGACCA<br><br>R: CACCACATCCAGCAAATCATTCTGATG	64	446
22	19	F: GACGTCGTCCTTATCAGAGTGAGCG<br><br>R: CCTTTCTAAAACGCCTCTAGCCAGC	64	336
23	20	F: GTTCTGGGAACATCAGGGCGAGTG<br><br>R: TGAATGGGAAATGAGAGGCAAGTTCCA	64	445
24	21	F: AAAGATGGATGAGAGGCCTTCACCAG<br><br>R: GTCCCCAAAGCTGGAGGCTAGCTCA	64	471

Abbreviations: F, forward primer; R, reverse primer; bp, base pairs; and T_m, annealing temperature.

Table C. UTR-specific PCR primers used for the mutation analysis of the *ATP7B* gene.

Sl.#	5'/3'UTRs	Primer sequence (5'→3')	T_m (°C)	Amplicon size (bp)
1	5'UTR	F:atagattggaaaatgtctcgtggctc; R: gccacgccgaacactagtctacg	56	592
2	5'UTR	F:ccgacccccgaactcaggaaacgct; R: agctgcgcagagccgggccgcag	58	572
3	5'UTR	F:gcagccaggcgcagagtccgagg; R: gcaaacaggggtccgggaaccgcc	62	592
4	3'UTR	F:tggactctagtccttggctggactg; R: tgtgtgagccaggaagcaggttcaag	62	446
5	3'UTR	F:ctctcagagcagcagaacgggttcag; R: tgacggacagcggaaatgtgctgcg	60	629
6	3'UTR	F: aaaatccaagcgcgctgttgattgtg; R: agccctcgtgaagacaagacggc	60	413

Abbreviations: F, forward primer; R, reverse primer; bp, base pairs; and T_m, annealing temperature.

Table D. Details of primers used in allele-specific PCR.

Sl.#	Mutation	Primer sequence (5'→3')	T_m (°C)	Amplicon size (bp)
1	c.172_173insC (p.A58fs*19)	F: GGCCCTTCTTCTCAGGTGGCCC<br>R: CTTTCCTTCTGCAATGCTGGCCTC	60	235
2	c.220_222delA (p.K74fs*9)	F: CATGACTTGCCAGTCATGTGTGAG<br>R: CTTTCCTTCTGCAATGCTGGCCTC	60	190
3	c.841C>T (p.Q281*)	F: GTCTTGAATATTGAAGAAAATATTGGCT<br>R: AGAACTGGAAGACCTGTGATCTGTC	54	214
4	c.1048delC (p.P350fs*12)	F: CTCATTCCCCTGGCTCCCCACG<br>R: GGGAGCAGGGCTCACCTATACCA	52	352
5	c.1442C>G (p.S481*)	F: ACATCTTGGCAAAGTCCCCACAATG<br>R: GTTATCAGGGCTACTGATAAACACAG	52	229
6	c.2255T>G (p.V752G)	F: TCTGGTCATCCTGGTGGTTGCTGG<br>R: CCTATTTCTTTAAGTCTGTCTCTATGC	58	246
7	c.2435delA (p.N812fs*2)	F: CGTTGTGACCCTTGGTGAGGACAT<br>R: CTTGCTTTCGTAGCTGGATTGAGAG	54	190
8	c.2534T>C (p.V845A)	F: GGAAAGTTTCCAGTGGATGGGAAAGC<br>R: TGGCATGGCTTCTCCTAGACGTAGG	58	191
9	c.2666A>C (p.H889P)	F: GCTCTGTGCTCATTAAAGCTACCCC<br>R: TCAGCCAGCTGCTGAATGGGTGC	52	322
10	c.3551insA (p.I1184fs*1)	F: ACAGACAGCCATCCTGGTGGCTAAT<br>R: TGGAAAACAGGCCTGAAATTAAGAGAG	50	166
11	c.3920T>A (p.V1307E)	F: CCTTATCAGAAATGATTTGCTGGATGA<br>R: CCTTTCTAAAACGCCTCTAGCCAGC	52	246

Abbreviations: F, forward primer; R, reverse primer; bp, base pairs; and T_m, annealing temperature.

Table E. Microsatellite markers flanking the *ATP7B* gene.

Sl.#	Microsatellite marker[a]	Physical distance (Mb) of the markers from the *ATP7B* gene[b]
1	D13S1325	0.028
2	D13S773	0.558
3	D13S1305	0.590

a, b The physical distance between the markers and *ATP7B* are according to UCSC Genome Bioinformatics site (http://genome.ucsc.edu/).

Table F. *In silico* analysis to determine if the novel missense variants identified in the present study are mutations.

Sl.#	Mutation	Family	PolyPhen-2 score	Mutation Taster score	SIFT score
1	c.2204T>G (p.Leu735Arg)	Family-M	Probably damaging with a score of 1	Disease causing with a p-value of 2.78	Damaging mutation with a p-value of 0
2	c.2255T>G (p.Val752Gly)	Family-41B	Benign with a score of 0.449	Disease causing with a p-value of 0.99	Damaging mutation with a p-value of 0
3	c.2534T>C (p.Val845Ala)	Family-80	Probably damaging with a score of 0.83	Disease causing with a p-value of 0.99	Damaging mutation with a p-value of 0
4	c.2666A>C (p.His889Pro)	Family-123	Probably damaging with a score of 1	Disease causing with a p-value of 2.10	Damaging mutation with a p-value of 0
5	c.3920T>A (p.Val1307Glu)	Family-49	Probably damaging with a score of 1	Disease causing with a p-value of 3.30	Damaging mutation with a p-value of 0
6	c.4015G>C (p.Ala1339Pro)	Family-C	Probably damaging with a score of 1	Disease causing with a p-value of 0.74	Damaging mutation with a p-value of 0

Table G. Non-disease causing variants identified in the *ATP7B* gene in Wilson disease families.

Sl.#	Variants	5’UTR/Exon(E)/Intron(I)	Family (zygosity)	dbSNP entry
1	c.-190C>G	5’UTR	Family-92 & Family-128 (Homozygous); Family-60, Family-506, Family-31, Family-72, Family-67, Family-77, Family-76 & Family-73 (Heterozygous)	Novel
2	c.-168T>G	5’UTR	Family-92 (Homozygous)	Novel
3	c.-128A>C	5’UTR	Family-72 & Family-84 (Heterozygous)	rs73184332
4	c.-142T>C		Family-32, Family-43, Family-52 & Family-122 (Homozygous); Family-W & Family-40 (Heterozygous)	Novel
5	c.-117_-118insCGCCG	5’UTR	Family-H, Family-K, Family-P, Family-Y, Family-85, Family-55, Family-T, Family-89, Family-73, Family-47, Family-49 & Family-35 (Homozyous); Family-72, Family-63, Family-34, Family-84 & Family-98 (Heterozygous)	rs148013251
6	c.-75C>A	5’UTR	Family-57, Family-Y, Family-47, Family-55, Family-50, Family-58, Family-78, Family-V, Family-82, Family-116, Family-419, Family-85, Family-28, Family-C, Family-F, Family-H, Family-I, Family-K, Family-O, Family-P, Family-R, Family-S, Family-T, Family-42, Family-45, Family-53, Family-60, Family-66, Family-73, Family-79, Family-95, Family-74, Family-87, Family-88, Family-98, Family-89, Family-94, Family-101, Family-47, Family-49, Family-35, Family-38 & Family-41 (Homozygous); Family-506, Family-34, Family-44, Family-W, Family-B, Family-E, Family-419, Family-G, Family-L, Family-72, Family-92, Family-63, Family-84, Family-74, Family-97 & Family-41B (Heterozygous)	rs2277448
7	c.261C>T (p.Ile87Ile)	E1	Family-54 (Heterozygous)	Novel
8	c.1216T>G (p.Ser406Ala)	E2	Family-28, Family-85, Family-H, Family-P, Family-T, Family-73, Family-95, Family-89, Family-47, Family-49, Family-47, Family-35 & Family-41 (Homozygous); Family 506, Family-W, Family-55, Family-G, Family-K, Family-	rs1801243

			72, Family-84 & Family-63 (Heterozyous)	
9	c.1366G>C (p.Val456Leu)	E3	Family-28, Family-P, Family-T, Family-95, Family-85, Family-89, Family-47, Family-49, Family-35 & Family-41 (Homozygous); Family-W, Family-506, Family-G, Family-H, Family-55, Family-63, Family-K, Family-72 & Family-84 (Heterozygous)	rs1801244
10	IVS4-53A>C	I4	Family-U, Family-T, Family-35, Family-47, Family-55, Family-82, Family-116, Family-V, Family-419, Family-41B, Family-58, Family-P, Family-85, Family-118, Family-S, Family-42, Family-50, Family-53, Family-O, Family-101, Family-73, Family-79 & Family-98 (Homozygous); Family-K, Family-70, Family-B, Family-G, Family-W, Family-L, Family-38, Family-84, Family-86, Family-87, Family-88, Family-74, Family-78, Family-89 & Family-97 (Heterozygous)	rs2147363
11	c.2196C>T (p.Leu732Leu)	E8	Family 73, Family-72, Family, 86 & Family, 97(Homozygous); Family-28, Family-34, Family-L, Family-63, Family-H & Family-I (Heterozygous)	rs201213995
12	c.2292C>T (p.Phe764Phe)	E8	Family-28, Family-H, Family-I, Family-95, Family-51 & Family-41 (Homozygous); Family-W (Heterozygous)	rs372979339
13	IVS10-25G>A	I10	Family-28, Family-H, Family-P, Family-85, Family-M, Family-T, Family-95, Family-47, Family-89, Family-94 & Family-35 (Homozygous); Family-W, Family-506, Family-E, Family-G, Family-K, Family-72, Family-84, Family-63, Family-55, Family-88, Family-98 & Family-97 (Heterozygous)	rs9526811
14	c.2484C>T (p.Gly828Gly)	E10	Family-D, Family-F, Family-N, Family-H, Family-A, Family-Q, Family-60, Family-62, Family-64, Family-69, Family-75 & Family-93 (Homozygous); Family-W, Family-70, Family-A & Family-34 (Heterozygous)	rs570594838
15	c.2495A>G (p.Lys832Arg)	E10	Family-28, Family-128, Family-C, Family-I, Family-116, Family-419, Family-I, Family-M, Family-P, Family-R, Family-S, Family-T, Family-42, Family-45, Family-53, Family-66, Family-73, Family-92, Family-95, Family-88, Family-98, Family74, Family-89, Family-94, Family-47, Family-49, Family-50, Family-55, Family-35, Family-41, Family-80, Family-81, Family-82 & Family-85 (Homozygous); Family-W, Family-506, Family-E, Family-	rs1061472

			G, Family-44, Family-K, Family-L, Family-K, Family-74, Family-M, Family-72, Family-78, Family-84, Family-56, Family-57, Family-97, Family-34 & Family-38 (Heterozygous)	
16	c.2855G>A (p.Arg952Lys)	E12	Family-28, Family-128, Family-C, Family-F, Family-H, Family-P, Family-R, Family-53, Family-92, Family-95, Family-56, Family-63, , Family-89, Family-94, Family-S, Family-42, Family-50, Family-T, Family-116, Family-419, Family-35, Family-55, Family-94, Family-97, Family-47, Family-49, Family-50, Family-35, Family-41, Family-80, Family-81, Family-82 & Family-85 (Homozygous); Family-W, Family-34, Family-L, Family-74, Family-506, Family-78, Family-E, Family-44, Family-K, Family-419, Family-G, Family-72, Family-84, Family-57, Family-87, Family-88, Family-98 & Family-38 (Heterozygous)	rs732774
17	c.2973G>A (Thr991Thr)	E13	Family-123 (Homozygous); Family-77 & Family-84 (Heterozygous)	rs1801246
18	c.2976C>T (p.Pro992Pro)	E13	Family-59, Family-91, Family-419 & Family-79 (Homozygous); Family-E, Family-44, Family-G & Family-56 (Heterozygous)	Novel
19	c.3009G>A (p.Ala1003Ala)	E13	Family-128, Family-82, Family-116, Family-419, Family-S, Family-42, Family-53 & Family-50 (Homozygous); Family-74, Family-L, Family-55, Family-56 & Family-97 (Heterozygous)	rs1801247
20	c.3237T>C (p.Cys1079Cys)	E14	Family-73 (Homozygous)	Novel
21	c.3419T>C (p.Val1140Ala)	E16	Family-28, Family-128, Family-C, Family-F, Family-H, Family-I, Family-M, Family-P, Family-S Family-116, Family-419, Family-T, Family-42, Family-53, Family-66, Family-55, Family-73, Family-74, Family-89, Family-94, Family-97, Family-47, Family-49, Family-50, Family-419, Family-P, Family-M, Family-34, Family-35, Family-80, Family-81, Family-82 & Family-85 (Homozygous); Family-506, Family-E, Family-W, Family-34, Family-78, Family-43, Family-G, Family-K, Family-L, Family-44, Family-72, Family-84, Family-92, Family-56, Family-57, Family-63, Family-74, Family-88 & Family-38 (Heterozygous)	rs1801249

22	IVS17-96A>G	I17	WD-72 (Heterozygous)	Novel
23	IVS18+6C>T	I18	Family-28, Family-128, Family-C, Family-F, Family-H, Family-I, Family-M, Family-50, Family-419, Family-82, Family-116, Family-85, Family-P, Family-R, Family-S, Family-T, Family-35, Family-55, Family-47, Family-42, Family-45, Family-66, Family-73, Family-95, Family-74, Family-89, Family-53, Family-94, Family-M, Family-49 & Family-60 (Homozygous); Family-506, Family-W, Family-U, Family-K, Family-38, Family-Q, Family-G, Family-E, Family-L, Family-63, Family-44, Family-72, Family-84, Family-57, Family-74, Family-88 & Family-98 (Heterozygous)	rs2282057
24	IVS19+50G>C	I19	Family-35, Family-47, Family-55, Family-P, Family-85, Family-S, Family-28, Family-128, Family-89, Family-H, Family-I, Family-M, Family-T, Family-42, Family-45, Family-50, Family-53, Family-66, Family-73, Family-95, Family-82 & Family-116 (Homozygous); Family-L, Family-W, Family-38, Family-43, Family-506, Family-K, Family-57, Family-72, Family-74, Family-84, Family-92, Family-56, Family-63, Family-87, Family-88, Family-98, Family-94, Family-40, Family-419 & Family-35 (Heterozygous)	rs9535795

Table H. Genotype-phenotype correlation in Wilson disease families.

Sl.#	Phenotypes	Genotypes		Significance of correlation
		**Missense mutations (Homozygous/Compound heterozygous)**	**Truncating (nonsense/insertion /deletion) mutations**	
1	Age of onset (years)	Average : 13.64 ± 0.15<br>n = 43	Average :11 ± 0.71<br>n = 13	*p-value* = 0.1996
2	Serum Copper<br>Normal range (75-160 mcg/dL)	Average : 35.47 ± 3.45<br>n = 38	Average : 35.87 ± 4.34<br>n =16	*p-value* = 0.9053
3	Serum Ceruloplasmin<br>Normal range (15-35	Average : 21.65 ± 3.77<br>n = 40	Average : 17.16 ± 6.47<br>n = 14	*p-value* = 0.8693
4	24 hr Urinary Copper<br>Normal range (upto 210 mcg/24 hr)	Average : 415.60 ± 57<br>n = 43	Average : 170.36 ± 73.49<br>n = 11	*p-value* = 0.5187
5	Kayser–Fleischer ring	45/46 (97.82%)	12/13 (92.30%)	*p-value* = 0.3241
6	Dysphagia	19/42 (45%)	9/13 (69.23%)	*p-value* = 0.1354
7	Dysarthria	31/44 (70.45%)	12/13 (92.30%)	*p-value* = 0.1116
8	**Tremor**	**24/43 (55.81%)**	**3/13 (23.07%)**	***p-value* = 0.0390**
9	**Dystonia**	**20/45 (44.44%)**	**11/12 (91.66%)**	***p-value* = 0.0030**
10	Writing difficulty	29/43 (67.44%)	12/13 (92.30%)	*p-value* = 0.0785
11	Chorea	7/47 (14.89%)	2/13 (15.38%)	*p-value* = 0.9657
12	Athetosis	1/41 (2.43%)	1/13 (7.69%)	*p-value* = 0.4031
13	Parkinsonism	19/41 (46.34%)	6/9 (66.66%)	*p-value* = 0.2788
14	**Rigidity**	**20/45 (4.44%)**	**10/13 (76.92%)**	***p-value* = 0.0396**

15	**Bradykinesia**	**7/45 (15.55%)**	**11/13 (84.61%)**	***p-value* < 0.0001**
16	Cerebral Atrophy	6/48 (12.5%)	1/13 (7.69%)	*p-value* = 0.6211
17	Cerebellar Atrophy	1/46 (2.17%)	1/12 (8.33%)	*p-value* = 0.3061
18	Brain Stem Atrophy	2/46 (4.34%)	2/12 (16.66%)	*p-value* = 0.1384
19	Giant Panda Sign	7/51 (13.72%)	1/14 (7.14%)	*p-value* = 0.5142
20	**Hepatomegaly**	**5/29 (17.24%)**	**6/12 (50%)**	***p-value* = 0.0315**
21	**Splenomegaly**	**4/33 (12.12%)**	**8/12 (66.66%)**	***p-value* = 0.0001**
22	Jaundice	10/28 (35.71%)	6/13 (46.15%)	*p-value* = 0.5356

*, p<0.05; **, p<0.01; and ***, p<0.00

Table I. Wilson disease affected individuals with ceruloplasmin levels and associated homozygous/compound heterozygous mutations.

Sl.#	Family name	Affected individual	Mutation	Serum ceruloplasmin (Normal range:15-35mg/dL)
1	Family-D	II-1	c.813C>A (p.Cys271*)	1
2	Family-N	IV-3	c.813C>A (p.Cys271*)	2
3	Family-Q	IV-3	c.813C>A (p.Cys271*)	4
4	Family-62	IV-3	c.813C>A (p.Cys271*)	2
5	Family-64	II-2	c.813C>A (p.Cys271*)	NA
6	Family-69	II-1	c.813C>A (p.Cys271*)	5
7	Family-75	IV-1	c.813C>A (p.Cys271*)	2
8	Family-93	IV-3	c.813C>A (p.Cys271*)	2.7
9	Family-419	IV-2	c.2131G>T (p.Gly711Trp))	4
10	Family-L	II-1	c.2131G>T (p.Gly711Trp) /c.3809A>G (p.Asp1270Ser)	4
11	Family-S	III-3	c.2131G>T (p.Gly711Trp)	4

12	Family-82	IV-1	c.2131G>T (p.Gly711Trp)	4
13	Family-116	V-4	c.2131G>T (p.Gly711Trp)	10
14	Family-42	IV-6	c.2131G>T (p.Gly711Trp)	2
15	Family-50	II-5	c.2131G>T (p.Gly711Trp)	3
16	Family-53	IV-2	c.2131G>T (p.Gly711Trp)	7
17	Family-V	IV-2	c.3182G>A (p.Gly1061Glu)	4
18	Family-O	II-1	c.3182G>A (p.Gly1061Glu)	4
19	Family-58	II-2	c.3182G>A (p.Gly1061Glu)	4
20	Family-41B	II-1	c.2255T>G (p.Val752Gly)/ c.3182G>A (p.Gly1061Glu)	3
21	Family-K	IV-1	c.4021G>A (p.Gly1341Ser)	10
22	Family-59	IV-2	c.3532A>G (p.Thr1178Ala)	7

23	Family-59	IV-3	c.3532A>G (p.Thr1178Ala)	NA
24	Family-91	IV-5	c.3532A>G (p.Thr1178Ala)	4
25	Family-P	II-1	c.2333G>A (p.Arg778Gln)	5
26	Family-85	IV-2	c.2333G>A (p.Arg778Gln)	8
27	Family-T	IV-6	c.3446G>C (p.Gly1149Ala)	2
28	Family-35	II-5	c.3446G>C (p.Gly1149Ala)	16
29	Family-35	II-6	c.3446G>C (p.Gly1149Ala)	1
30	Family-47	IV-6	c.3446G>C (p.Gly1149Ala)	5
31	Family-55	II-1	c.2303C>T (p.Pro768Leu)/ c.3446G>C (p.Gly1149Ala)	8.6
32	Family-83	IV-1	c.3008C>T (p.Ala1003Val)	6
33	Family-36	IV-3	c.3008C>T (p.Ala1003Val)	4
34	Family-54	IV-4	c.3008C>T (p.Ala1003Val)	18
35	Family-128	IV-5	c.220_222delA (p.Lys74fs*9)	6
36	Family-31	II-3	c.841C>T (p.Gln281*)	5

37	Family-B	II-3	c.172_173insC (p.Ala58fs*19)/ c.3741C>G (p.His1247Gln)	2
38	Family-M	II-1	c.2204T>G (p.Leu735Arg)	4
39	Family-80	IV-1	c.2435delA (p.Asn812fs*2)	NA
40	Family-32	V-5	c.3301G>A (p.Gly1101Arg)	4
41	Family-40	II-2	c.3301G>A (p.Gly1101Arg)	5
42	Family-43	IV-1	c.3301G>A (p.Gly1101Arg)	4
43	Family-52	IV-2	c.3301G>A (p.Gly1101Arg)	5
44	Family-46	II-1	c.2998G>A (p.Gly1000Arg)	9
45	Family-49	IV-1	c.3920T>A (p.Val1307Glu)	2
46	Family-60	V-1	c.1048delC (p.Pro350fs*12)	6
47	Family-63	II-3	c.3053C>T (p.Ala1018Val)/ c.3809A>G (p.Asp1270Ser)	3
48	Family-89	II-5	c.3809A>G (p.Asp1270Ser)	3
49	Family-72	IV-1	c.2267C>T (p.Ala756Val) & c.2297C>T (p.Thr766Met)	NA
50	Family-72	IV-2	c.2267C>T (p. Ala756Val) & c.2297C>T (p.Thr766Met)	2
51	Family-72	IV-3	c.2267C>T (p.Ala756Val) & c.2297C>T (p.Thr766Met)	4
52	Family-76	IV-2	c.2930C>T (p.Thr977Met)	5

53	Family-90	IV-1	c.172_173insC (p.Ala58fs*19) & c.3741C>G (p.His1247Gln)	5
54	Family-86	II-2	c.3551insA (p.Ile1184fs*1)	6
55	Family-98	II-2	c.3722C>T (p.Ala1241Val)	3.5
56	Family-100	IV-3	c.3008C>T (p.Ala1003Val)	7
57	Family-101	IV-3	c.3182G>A (p.Gly1061Glu)	5
58	Family-118	IV-4	c.2998G>A (p.Gly1000Arg)	8
59	Family-122	IV-2	c.3301G>A (p.Gly1101Arg)	6
60	Family-123	II-2	c.2666A>C (p.His889Pro)/ c.3155C>T (p.Pro1052Leu)	6
61	Family-73	IV-2	c.3236G>T (p.Cys1101Arg)	26

Table J. Known mutations in *ATP7B*

Sl. #	Mutation	Exon/intron	Nature of mutation	Area of protein	Ethnic origin of family	Reference
1	c.-441_-427del	Promoter	Deletion	Before CBD1	Saridian	1
2	c.-133A>C	Promoter	Substitution	Before CBD1	Taiwanese	2
3	c.-215A>T	Promoter	Substitution	Before CBD1	Taiwanese	2
4	c.-129_-125del	5' UTR	Deletion	Before CBD1	Japanese, North American	3, 4
5	c.-36C>T	5'UTR	Substitution	Before CBD1	Chinese	5
6	c.-75A>C	5'UTR	Substitution	Before CBD1	Japanese	3
7	c.19_20del (p.Gln7Aspfs*15)	1	Deletion	Before CBD1	Czechs/Slovaks	6
8	c.51+1G>A	1	Splice site	Before CBD1	Chinese	7
9	c.51+4A>T	1	Splice site	Before CBD1	Brazil	8
10	c.121A>G (p.Asn41Asp)	2	Missense	Before CBD1	Chinese	9
11	c.122A>G (p.Asn41Ser)	2	Missense	Before CBD1	Brazil	8
12	c.130T>A (p.Tyr44Asn)	2	Missense	Before CBD1	Chinese	10
13	c.174dupC (p.Thr59Hisfs*19)	2	Duplication	CBD1	Indian (East)	11
14	c.174delC (p.Thr59Profs*9)	2	Deletion	CBD1	Indian (West)	12
15	c.199A>G (p.Met67Val)	2	Missense	CBD1	Indian (East and West)	13
16	c.213_214del (p.Val73Glufs*4)	2	Deletion	CBD1	Sardinian, Bulgarian	14
17	c.216T>A (p.Cys72*)	2	Nonsense	CBD1	Italian	15
18	c.254G>T (p.Gly85Val)	2	Missense	CBD1	Turkey, British, Chinese-Han	1, 16, 17
19	c.267dupG (p.Lys90Glufs*73)	2	Duplication	CBD1	Turkish	18
20	c.268_271del p.Lys90fs*10	2	Deletion	CBD1	Chinese	7
21	c.283C>T (p.Gln95*)	2	Nonsense	CBD1	Italian	19
22	c.287G>A/A>G (p.Gly96Asp/p.Asp96Gly)	2	Missense	CBD1	Chinese-Han	20
23	c.314C>A (p.Ser105*)	2	Nonsense	CBD1	German, Chinese (Hong Kong), Chinese-Han Chinese	21, 22, 23, 24
24	c.328C>T (p.Gln110*)	2	Nonsense	CBD1	Turkish	18
25	c.330delA (p.Gln110Hisfs*43)	2	Deletion	CBD1	Egyptian	25
26	c.331C>T (p.Gln111*)	2	Nonsense	CBD1	Italian, Spanish	1, 26
27	c.343C>T (p.Gln115*)	2	Nonsense	CBD1	Yugoslavia	27
28	c.365_366delinsTTCGAAGC (p.Glu122fs)	2	Deletion/Insertion	CBD1	Indian (West)	28
29	c.367delG (p.A123Pfs*30)		Deletion	CBD1	Chinese	7
30	c.397delT (p.Trp133Glyfs*20)	2	Deletion	CBD1	Greek (Crete)	29
31	c.406A>G (Arg136Gly)	2	Missense	Between CBD1 & CBD2	Indian (Eastern and western)	13
32	c.433G>T (Val145Phe)	2	Missense	CBD2	Chinese	24
33	c.442C >T (p.Arg148Trp)	2	Missense	CBD2	Indian (West)	28
34	c.448_452del (p.Glu150Hisfs*11)	2	Deletion	CBD2	Indian (East), Indian (West)	11, 12
35	c.453delC (p.Met152*)	2	Nonsense	CBD2	Japanese, central Japan	31, 32

36	c.463C>T (p.Gln155*)	2	Nonsense	CBD2	Italian	15
37	c.470G>T (p.Cys157Phe)	2	Missense	CBD2	Chinese	32
38	c.507delA (p.Gly170Glufs*2)	2	Deletion	CBD2	Egyptian	25
39	c.509G>T (p.Gly170Val)	2	Missense	CBD2	Italy	33
40	c.522_523insC	2	Insertion	CBD2	Thai	34
41	c.523insA	2	Insertion	CBD2	Chinese, Taiwan, American	35, 36
42	c.524_525del (p.Val176Serfs*28)	2	Deletion	CBD2	German	37
43	c.525dupA (p.Val176Serfs*28)	2	Duplication	CBD2	Chinese, Chinese-Han, Chinese (Hong Kong), Thai, Taiwanese	5, 7, 22, 34, 35
44	c.525_526del (p.Lys175Asn*28)	2	Deletion	CBD2	British	16
45	c.561T>A (p.Tyr187*)	2	Nonsense	CBD2	Indian (East), Indian (West)	11, 12
46	c.561_563del (p.Tyr187*)	2	Nonsense	CBD2	British	38
47	c.562C>T (p.Gln188*)	2	Nonsense	CBD2	Serbian	15
48	c.650T>G (p.Leu217*)	2	Nonsense	CBD3	Scottish	39
49	c.654_655del (p.Leu219Glyfs*4)	2	Deletion	CBD3	French	40
50	p.685insA	2	Insertion	CBD3	Taiwanese	2
51	c.678delG (p.Leu227fs)	2	Deletion	CBD3	Indian (West)	28
52	c.695delC (p.P232Qfs*30)	2	Deletion	CBD3	Chinese	7
53	c.748G>A (p.Gly250Arg)	2	Missense	CBD3	Chinese	9
54	c.778delC (p.Gln260Asn*2)	2	Deletion	CBD3	German	21
55	c.778dupC (p.Gln260Profs*10)	2	Duplication	CBD3	German	21
56	c.779_780insC (p.Gln260Hisfs*10)	2	Insertion	CBD3	Greek (Romanian)	41
57	c.802_808del (p.Cys268Leufs*4)	2	Deletion	CBD3	German	42
58	c.813C>A (p.Cys271*)	2	Nonsense	CBD3	Indian (East), Tukish, Indian (West), Thai, German, Indian (South)	11, 16 , 28, 34, 43, 44
59	c.813delC (p.Cys271Trpfs*3)	2	Deletion	CBD3	Chinese	32
60	c.815dupT (p.Asn274Glufs*2)	2	Duplication	CBD3	Pakistani	45
61	c.845delT (p.Leu282Profs*2)	2	Deletion	CBD3	Greek, Middle Eastern, Jewish-Iran, Jewish-Iraq, Bulgarian,	47, 4, 46, 48
62	c.846delC	2	Deletion	CBD3	Iranian, Greek	49, 50
63	c.847delC (p.Leu283*)	2	Nonsense	CBD3	Iranian, Greek	49, 50
64	c.856C>T (p.Gln286*)	2	Nonsense	CBD3	Italy, Greek (Crete), N.European, Greek Middle Eastern, Shephardic Jewish	1, 29, 39, 47, 51
65	c.892delC (p.Gln298Lysfs*2)	2	Deletion	CBD3	Indian (East), Indian (West)	11,12
66	c.898_902delAAGTA (p.Lys300*)	2	Deletion	CBD3	Chinese	9
67	c.915T>A (p.Cys305*)	2	Nonsense	CBD3	Italy	1, 47
68	c.918_931del (p.Ser307Alafs*13)	2	Deletion	CBD3	German	39

69	c.994 G>T (p.Glu332*)	2	Nonsense	CBD3	Chinese	7, 32
70	c.985G > C (p.Asp329His)	2	Missense	CBD3	Thai	34
71	c.997G>A (p.Gly333Arg)	2	Missense	Between CBD3 & CBD4	Indian (South)	44
72	c.1057delC	2	Deletion	Between CBD3 & CBD4	Chinese	52
73	c.1063C>T (p.Gln355*)	2	Nonsense	Between CBD3 & CBD4	Polish	53
74	c.1073delG (p.Cys358Serfs*5)	2	Deletion	CBD4	British, German	39
75	c.1136delG (p.Gly379Alafs*2)	2	Deletion	CBD4	Italy, Greek	1, 47
76	c.1142T>G (p.Ile381Ser)	2	Missense	CBD4	Polish	54
77	c.1143_1149del (p.Ser382Trpfs*24)	2	Deletion	CBD4	British	16
78	c.1162C>T (p.Q388*)	2	Nonsense	CBD4	Chinese	10, 24
79	c.1186G>T (p.Glu396*)	2	Nonsense	CBD4	Egyptian	25
80	c.1234G>T (p.Glu412*)	2	Nonsense	CBD4	Korean	55
81	c.1285+2T>A	2	Splice site	CBD4	German	21
82	c.1285+5G>T	2	Splice site	CBD4	Sardinian	47
83	EX2 DEL	2	Deletion	CBD1-CBD4	Chinese	9
84	c.1286-2A>G	3	Missense	Between CBD4 & CBD5	Italian	15
85	c.1292_1293del (p.Cys431Phefs*3)	3	Deletion	Between CBD4 & CBD5	Japanese	3
86	c.1340_1343del (p.Gln447Leufs*50)	3	Deletion	Between CBD4 & CBD5	Czech, German	4, 56, 57
87	c.1366 G>C	3	Missense	Between CBD4 & CBD5	Chinese	58
88	c.1369C>T (p.Gln457*)	3	Nonsense	Between CBD4 & CBD5	Turkish	16
89	c.1384_1400del (p.His462Cysfs*30)	3	Deletion	Between CBD4 & CBD5	Chinese	59
90	c.1403_1416del (p.A468Gfs*33)	3	Deletion	Between CBD4 & CBD5	Chinese	7
912	c.1436delC (p.Pro479Hisfs*19)	3	Deletion	Between CBD4 & CBD5	Brazilian	8
92	c.1448_1455del (p.Arg483Serfs*20)	3	Deletion	CBD5	Chinese	32
93	c.1449_1456del (p.Arg483Serfs*20)	3	Deletion	CBD5	Chinese	7
94	c.1456G>T (p.Ala486Ser)	3	Missense	CBD5	Greek	41
95	c.1470C>A (p.Cys490*)	3	Nonsense	CBD5	Thai, Taiwanese	34, 35
96	c.1475T>C (p.Leu492Ser)	3	Missense	CBD5	Italian, Greek, American	1, 47, 60, 61
97	c.1492A>T (p.Thr498Ser)	3	Missense	CBD5	Chinese	24
98	c.1512dupT (p.Asn505*)	3	Duplication	CBD5	Sardinian, Mediterranean	18, 62
99	c.1514_1515insT (p.Ile506Hisfs*28)	3	Insertion	CBD5	Sardinian	14
100	c.1516_1517del (p.Ile506Argfs*27)	3	Deletion	CBD5	Chinese-Han	17
101	c.1518_1521del (p.Glu507Glyfs*16)	3	Deletion	CBD5	British, Polish	16, 53
102	c.1518_1522del (p.Ile506Metfs*26)	3	Deletion	CBD5	Czechs/Slovaks	6
103	c.1531C>T (p.Gln511*)	3	Nonsense	CBD5	Chinese, Chinese-Han	23, 63
104	c.1543+1G>T	3	Missense	CBD5	Italian, Brazilian, Chinese, Greek, Korean	1, 8, 23, 47, 55
105	c.1543+1G>A	3	Splice site	CBD5	Italian	54
106	c.1544-2A>G	4	Splice site	CBD5	Italian	15
107	c.1544-2A>C	4	Splice site	CBD5	Italy	33

108	c.1544G>T (p.Gly515Val)	4	Missense	CBD5	Chinese	63
109	c.1544G >A (p.Gly515Asp)	4	Missense	CBD5	Indian (West)	28
110	c.1545delT (p.G515Gfs*9)	4	Nonsense	CBD5	Chinese	7
111	c.1552_1553delTC (p.Ser518Argfs*15)	4	Deletion	CBD5	Chinese	7
112	c.1555G>A (p.Val519Met)	4	Missense	CBD5	American	64
113	c.1568T>A (p.Leu523*)	4	Nonsense	CBD5	British	38
114	c.1594T>C (p.Tyr532His)	4	Missense	CBD5	Scottish	38
115	c.1607T>C (p.Val536Ala)	4	Missense	CBD5	North European	39
116	c.C>T1616 (p.Pro539Leu)	4	Missense	CBD5	Austria	65
117	c.1621G>A (p.Glu541Lys)	4	Missense	CBD5	Greek	66
118	c.1630C>T (p.Gln544*)	4	Nonsense	CBD5	Finnish, American	36, 67
119	c.1639delC (p.Gln547Argfs*22)	4	Deletion	CBD5	Palestinian	46
120	c.1646T>C (p.Leu549Pro)	4	Missense	CBD5	Egyptian	25
121	c.1646_1652del (p.Gly550Argfs*17)	4	Deletion	CBD5	Chinese	68
122	c.1672_1673del (p.Asp558Leufs*9)	4	Deletion	CBD5	Italian	1, 47
123	c.1705_1707+8del	4	Deletion	CBD6	British	16
124	c.1707+2dupT	4	Splice site	CBD6	Turkish, Greek, Italian	1, 16, 47, 69
125	c.1707+5G>A	4	Splice site	CBD6	Egyptian	25
126	c.1707+11dupGT	4	Splice site	CBD6	Indian (North)	71
127	c.1708-?_1946+?del		Ex5-6del	CBD6	Danish	72
128	c.1708-1G>C	5	Splice site	CBD6	Chinese, Indian (East), British, Chinese, Spanish, Thai, Taiwanese, Greek, Cypriot, Indian, Chinese-Han, Bulgarian	5, 9, 11, 16, 24, 26, 34, 35, 41, 48, 49, 50, 70
129	c.1708-2A>G	5	Splice site	CBD6	Turkish (Bulgaria)	73
130	c.1708-5T>G	5	Splice site	CBD6	Chinese-Han, Chinese (Hong Kong), Japanese	5, 22, 74
131	c.1711-1G>C	5	Splice Site	CBD6	Indian	49, 50
132	c.1716delG (p.Met573fs)	5	Deletion	CBD6	Indian (West)	28
133	c.1721_1722insTGAC (p.His580Serfs*105)	5	Insertion	CBD6	Chinese	68
134	c.1743_1744dup (p.Ile582Thrfs*2)	5	Duplication	CBD6	Italian	15
135	c.1744_1745del (p.Ile582Argfs*25)	5	Deletion	CBD6	British, N. European, British, Italian, French	16, 18, 40, 49, 50, 62
136	c.1745_1746 delTA	5	Deletion	CBD6	Chinese	9
137	c.1747_1748insT (p.Asn581Serfs*232)	5	Insertion	CBD6	Indian	49, 50
138	c.1748_1749del (p.Glu583Valfs*24)	5	Deletion	CBD6	Italian	15
139	c.1771G>A (p.Gly591Ser)	5	Missense	CBD6	Indian (East)	75, 76
140	c.1772G>A (p.Gly591Asp)	5	Missense	CBD6	Mediterranean, Egyptian, European, Italian	18, 25, 38, 60, 61, 62

141	c.1782delT (p.Tyr594*)	5	Nonsense	CBD6	Italian, Mediterranean, , Greek	14, 18, 41, 62
142	c.1783G>A (p.Ala595Thr)	5	Missense	CBD6	Indian (Eastern and western)	13
143	c.1739delA (p.His580Profs)	5	Deletion	CBD6	Italy	33
144	c.1803delC (p.Ser602Alafs*46)	5	Deletion	CBD6	Chinese-Han	17
145	c.1810G>C (p.Ala604Pro)	5	Missense	CBD6	British	38, 60
146	c.T>G1817 (p.Val606Gly)	5	Missense	CBD6	Chinese	10
147	c.1820dupA (p.Phe608Valfs*2)	5	Duplication	CBD6	Chinese	68
148	c.1823_1825del (p.Phe607_Asp608delinsTyr)	5	Deletion	CBD6	Italian	1, 47
149	c.1831G>A (p.Glu611Lys)	5	Missense	CBD6	Chinese	32
150	c.1846C (p.Arg616Trp)	5	Missense	CBD6	German	56, 57, 60
151	c.1847G>A (p.Arg616Gln)	5	Missense	CBD6	British, Mediterranean, Bulgarian, Polish, Indian (South), Chinese-Han, Bulgarian	16, 23, 27, 44, 53, 70
152	c. 1747delAT	5	Deletion	CBD6	Britian	49, 50
153	c.1849dupG (p.Asp617Glyfs*7)	5	Duplication	CBD6	Indian (North)	71
154	c.1849delG (p.Asp617Ilefs)	5	Deletion	CBD6	Italy	33
155	c.1869+1_4delGT AA	6	Deletion	CBD6	Indian (West)	28
156	c.1870-2A>G	6	Spice site	CBD6	Bulgarian	73
157	c.1870-2delA	6	Spice site	CBD6	Korean	77
158	c.1875-1876insAATT (p.Gly626fs)	6	Insertion	CBD6	Chinese	78
159	c.1877G>C (p.Gly626Ala)	6	Missense	CBD6	Sardinian, Italian, Turkish, Jewish, N. European, Middle Eastern, Mediterranean, Bulgarian	4, 14, 18, 51, 62, 70
160	c.1883_1884del (p.His628Argfs*126)	6	Deletion	CBD6	Turkish	14
161	c.1915C>T (p.His639Tyr)	6	Missense	Between CBD6 & TMS1	Polish	53
162	c.1922T>C (p.Leu641Ser)	6	Missense	Between CBD6 & TMS1	Scottish, Czechs/Slovaks	6, 38
163	c.1924G>C (p.Asp642His)	6	Missense	Between CBD6 & TMS1	Italian, Egyptian	1, 25, 47
164	c.1927ins10	6	Insertion	Between CBD6 & TMS1	German	79
165	c.1934T>G (p.Met645Arg)	6	Missense	Between CBD6 & TMS1	Jewish, Ashkenazi Jewish, Italian, Spanish- Grand Canaria, Spanish, Spanish (Columbian), American	1, 4, 8, 15, 26, 36, 46, 47, 80
166	c.1946+2T>G	6	Splice site	Between CBD6 & TMS1	Indian (west)	28
167	c.1946+5dupG	6	Splice site	Between CBD6 & TMS1	British	16
168	c.1946+9A>G	6	Splice site	Between CBD6 & TMS1	Chinese-Han	23
169	IVS6+1G>A	6	Splice site	Between CBD6 & TMS1	Chinese	81
170	c.1947-4C>T	7	Splice site	Between CBD6 & TMS1	Korean	77
171	c.1947-2A>G	7	Splice site	Between CBD6 & TMS1	Chilean	38
172	c.1947-1G>C	7	Splice site	Between CBD6 & TMS1	French	40
173	c.1947delG	7	Deletion	Between CBD6 &	Central Japan	32

				TMS1		
174	c.1950G>A (p.Trp650*)	7	Nonsense	Between CBD6 & TMS1	Chinese-Han	5
175	c.1958C>A (p.Ser653Tyr)	7	Missense	Between CBD6 & TMS1	Polish	53
176	c.1963delC (p.Leu655Cysfs*13)	7	Deletion	TMS1	Indian (South)	44
177	c.1968C>A (p.Cys656*)	7	Nonsense	TMS1	Korean, Chinese	59, 82
178	c.1969A>C (p.Ser657Arg)	7	Missense	TMS1	North European	39
179	c.1994T>G (p.Met665Arg)	7	Missense	TMS1	Chinese	9
180	c.1995G>A (p.Met665Ile)	7	Missense	TMS1	Albanian	1, 47
181	c.2004_2006del (p.Met668del)	7	Deletion	TMS1	Japanese	3
182	c.2012_2013 insATAT	7	Insertion	TMS1	Chinese	9
183	c.2007_2013del (p.Tyr670*)	7	Deletion	TMS1	Icelandic, British	16, 49, 50
184	c.2008_2013del (p.Tyr670_Met671del)	7	Deletion	TMS1	German	42
185	c.2027A>T (p.Asn676Ile)	7	Missense	Between TMS1 & TMS2	Hungarian	83
186	c.2035delC (p.His679Thrfs*17)	7	Deletion	Between TMS1 & TMS2	Sardinian	15
187	c.2043delC (p.S681Sfs*15)	7	Deletion	Between TMS1 & TMS2	Chinese	7
188	c.2049_2053delCCTGGinsTTTC (p.Val683_Leu684delinsVal)	7	Deletion-Insertion	Between TMS1 & TMS2	Egyptian	84
189	c.2069C>T (p.Pro690Leu)	7	Missense	TMS2	Spanish	26
190	c.2071G>A (p.Gly691Arg)	7	Missense	TMS2	Turkish, German, Indian (West)	1, 28, 47
191	p.Leu692Pro	7	Missense	TMS2	Chinese	85
192	2075T>C (p.Leu692Pro)	7	Missense	TMS2	Chinese	86
193	c.2077T>C (p.Ser693Pro)	7	Missense	TMS2	Chinese-Han	23
194	c.2078C>G (p.Ser693Cys)	7	Missense	TMS2	Chinese-Han	23
195	c.2078C>A (p.Ser693Tyr)	7	Missense	TMS2	Hungarian	83
196	c.2097_2099del (p.Phe700del)	7	Deletion	TMS2	Chinese-Han	17
197	c.2101_2102del (p.Ile701Leufs*53)	7	Deletion	TMS2	Greek	41
198	c.2108G>A (p.Cys703Tyr)	7	Missense	TMS2	British, Egyptian	25, 38
199	c.2115delGT (p.Phe705Pro-fs)	7	Deletion	TMS2	Indian	71
200	c.2116_2117del (p.Val706Profs*48)	7	Deletion	TMS2	Indian (North)	71
201	c.2120A>G (p.Gln707Arg)	7	Missense	TMS2	Chinese	78
202	c.2121+3A>T	7	Splice Site	TMS2	Chinese	78
203	c.2121+3A>G	7	Splice site	TMS2	Polish, Italian	53, 87
204	c.2121+127C>A	7	Splice site	TMS2	Indian	75, 76
205	c.2122-8T>G	8	Splice site	TMS2	Italian, Sardinian	69, 88
206	c.2123T>C (p.Leu708Pro)	8	Missense	TMS2	American, Spanish-Grand Canaria, Brazilian	4, 8, 80
207	c.2128G>C (p.Gly710Arg)	8	Missense	TMS2	Italian	19
208	c.2128G>A (p.Gly710Ser)	8	Missense	TMS2	N. European, Middle Eastern, Italian, Middle Eastern, German, Hungarian, Indian (East), Chinese	4, 9, 11, 51, 56, 57, 60, 89,
209	c.2129G>C (p.Gly710Ala)	8	Missense	TMS2	German	43

210	p.Gly710Cys	8	Missense	TMS2	Chinese	30
211	c.2129G>T (p.Gly710Val)	8	Missense	TMS2	Italian	38
212	c.2131G>A (p.Gly711Arg)	8	Missense	TMS2	Italian	18, 62
213	c.2131G>T (p.Gly711Trp)	8	Missense	TMS2	Pakistani	16
214	c.2132G>A (p.Gly711Glu)	8	Missense	TMS2	Italian	19
215	c.2138A>G (p.Tyr713Cys)	8	Missense	TMS2	Italian	19
216	c.2143T>C (p.Tyr715His)	8	Missense	TMS2	Hungarian	83
217	c.2145C>A (p.Tyr715*)	8	Missense	TMS2	Indian (South)	44
218	c.2145C>T (p.Tyr715Tyr)	8	Silent	TMS2	Indian (West)	28
219	c.2157C>A (p.Y719*)	8	Nonsense	TMS2	Chinese	7, 30
220	c.2160delA (p.Lys720Asnfs*3)	8	Deletion	Between TMS2 & TMS3	Italian	49, 50
221	c.2161T>C (p.Ser721Pro)	8	Missense	Between TMS2 & TMS3	Spanish	69
222	c.2165dupT (p.Arg723Glufs*32)	8	Duplication	Between TMS2 & TMS3	Japanese, Chinese	3, 68
223	c.2180C>T (p.Ala727Val)	8	Missense	Between TMS2 & TMS3	Chinese	
224	c.2180C>A (p.Ala727Asp)	8	Missense	Between TMS2 & TMS3	Unknown	54
225	p.Asn728Ser	8	Missense	Between TMS2 & TMS3	Chinese	85
226	c.2185A>G (p.Met729Val)	8	Missense	Between TMS2 & TMS3	Korean	55
227	c.2189A>G (p.Asp730Gly)	8	Missense	Between TMS2 & TMS3	Indian (West)	28
228	c.2192T>A (p.Val731Glu)	8	Missense	TMS3	Chinese	68
229	c.2195T>A (p.Leu732His)	8	Missense	TMS3	Chinese	10
230	c.2195T>C (p.Leu732Pro)	8	Missense	TMS3	Chinese	10
231	c.2203_2231del (p.Leu735Serfs*10)	8	Deletion	TMS3	Japanese	3
232	c.2210C>G (p.Thr737Arg)	8	Missense	TMS3	Czech/Slovak	6, 90
233	c.2222A>G (p.Tyr741Cys)	8	Missense	TMS3	German	43
234	c.2223T>A (p.Tyr741*)	8	Nonsense	TMS3	Italian	18, 62
235	c.2227delT (p.Tyr743fs)	8	Deletion	TMS3	Indian (West)	28
236	c.2224insA (p.Val742Aspfs*13)	8	Insertion	TMS3	Indian (North)	71
237	c.2230T>C (p.Ser744Pro)	8	Missense	TMS3	Arabic (Kuwait), Saudi Arabian	16, 91
238	c.2231T>C (p.Ser744Pro)	8	Missense	TMS3	Egyptian	84
239	c.2231C>T (p.Ser744Phe)	8	Missense	TMS3	Chinese	9
240	c.2239A>T (p.Ile747Phe)	8	Missense	TMS3	Sardinian	1, 47
241	c.2258insC (p.Ala753*)	8	Insertion	TMS3	Indian	71
242	c.2258dupC (p.Glu754*)	8	Nonsense	TMS3	Indian (North)	71
243	c.2267C>G (p.Ala756Gly)	8	Missense	Between TMS3 & TMS4	Chinese	38
244	c.2267C>T (p.Ala756Val)	8	Missense	Between TMS3 & TMS4	Italian	92
245	c.2273G>T (p.Arg758Met)	8	Missense	Between TMS3 & TMS4	Chinese	93
246	c.2279C>T (p.Pro760Leu)	8	Missense	Between TMS3 & TMS4	German, European (German, Polish)	56, 57, 60, 94
247	c.2280_2301del (p.Val761Profs*39)	8	Deletion	Between TMS3 & TMS4	British	16

248	c.2288T>A (p.Phe763Tyr)	8	Missense	TMS4	Finnish	40
249	p.Phe764Leu	8	Missense	TMS4	Chinese	30
250	c.2292_2312del (p.Asp765_Phe771del)	8	Deletion	TMS4	Indian	95
251	c.2293G>A (p.Asp765Asn)	8	Missense	TMS4	Italian, Yemenite-Jewish, Moroccan, British, German, Polish, Egyptian	14, 16, 25, 46, 53, 56, 57, 60, 96, 97, 98
252	c.2293G>C (p.Asp765His)	8	Missense	TMS4	Italy	33
253	c.2294A>G (p.Asp765Gly)	8	Missense	TMS4	Chinese-Han, Chinese	17, 24
254	c.2295A>C (p.Met769Leu)	8	Missense	TMS4	Hungarian	83
255	c.2296A>C (p.Thr766Pro)	8	Missense	TMS4	Chinese	68
256	c.2296dupA (p.Thr766Asnfs*29)	8	Duplication	TMS4	Brazilian	8
257	c.2297C>T (p.Thr766Met)	8	Missense	TMS4	Chinese, British	38, 68
258	c.2297C>G (p.Thr766Arg)	8	Missense	TMS4	British	99
259	c.2298_2299insC (p.Pro767Profs)	8	Insertion	TMS4	Austrian	65
260	c.2299C >T	8	Unknown	TMS4	Thai	34
261	c.2299insG (p.Pro767Argfs*28)	8	Insertion	TMS4	Mediterranean, Greek	1, 47
262	2299insC	8	Insertion	TMS4	Japanese (central Japan)	3, 32
263	c.2302insT	8	Insertion	TMS4	American	36
264	c.2302insC	8	Insertion	TMS4	Italian, British	49, 50
265	c.2303C>A (p.Pro768His)	8	Missense	TMS4	Korean	82
266	c.2303C>T (p.Pro768Leu)	8	Missense	TMS4	Indian (South), Spanish	44, 100
267	c.2304dupC (p.Met769Hisfs*26)	8	Duplication	TMS4	Sardinian, Italian, Turkish, Albanian, Italian, British, N. European, Japanese, Russian, American, Taiwanese, French, British, Mediterranean, Sardinian, Japanese, Taiwanese, Greek, Cypriot, German, Korean, Chinese-Han, Brazilian, Polish, Indian (East), Spanish, Bulgarian, Chinese, Egyptian, Sardinian, Czech, Yugoslavian, Indian (West)	3, 4, 8, 11, 14, 16, 17, 18, 25, 26, 27, 28, 35, 41, 48, 49, 50, 53, 56, 57, 62, 63, 70, 82, 88, 90, 97, 101, 102
268	c.2304delC (p.Met769Cysfs*38)	8	Deletion	TMS4	Korean, Chinese (Hong Kong)	77, 103
269	c.2304_2305 insC	8	Insertion	TMS4	Chinese	9
270	c.2305A>G (p.Met769Val)	8	Missense	TMS4	British, Spanish-Grand Canaria, German, Chinese-Han, Italian	5, 15, 16, 49, 50, 56, 57, 60, 80, 98
271	c.2306T>G (p.Met769Arg)	8	Missense	TMS4	British	16
272	c.2307G>T (p.Met769Ile)	8	Missense	TMS4	Japanese	104
273	c.2308C>T (p.Leu770Phe)	8	Missense	TMS4	Chinese	9
274	c.2310C>G (p.Leu770Leu)	8	Silent	TMS4	Chinese	9

275	c.2311C > G (Leu770Leu)	8	Silent	TMS4	Iranian	105
276	c.2326C>G (p.Leu776Val)	8	Missense	TMS4	British	49, 50, 60, 96
277	c.2327T>C (p.Leu776Pro)	8	Missense	TMS4	Polish	53
278	c.2332C>G (p.Arg778Gly)	8	Missense	TMS4	Turkish, Greek, Mediterranean, Bulgarian, Polish, Hungarian, Bulgarian, Czech	1, 14, 18, 47, 53, 62, 70, 83, 90
279	c.2332C>T (p.Arg778Trp)	8	Missense	TMS4	American, Italian, Sardinian, British, Sardinian, Spanish-Grand Canaria, Japanese, Bulgarian, Italian	1, 4, 16, 15, 18, 47, 62, 70, 80, 96, 104
280	c.2333G>A (p.Arg778Gln)	8	Missense	TMS4	Taiwanese, Chinese-Han, Chinese, Italian, Korean, Indian (West), Chinese	5, 9, 15, 28, 55, 60, 68, 96, 106
281	c.2333G>T (p.Arg778Leu)	8	Missense	TMS4	Japanese, Chinese, Chinese-Han, Chinese - Han, Hui,Thai, Taiwanese, Korean	3, 5, 9, 17, 24, 34, 49, 50, 60, 77, 96,106, 107
282	c.2335T>G (p.Trp779Gly)	8	Missense	TMS4	Iranian	108, 109
283	c.2335T>G (p.Trp779Gly)	8	Missense	TMS4	Austrian	65
284	c.2336G>A (p.Trp779*)	8	Nonsense	TMS4	N. European, Middle Eastern, Anglo Saxon, German, Polish, Eritrean, Czech	1, 4, 15, 47, 49, 53, 56, 57, 60, 90
285	c.2336G>A (p.Trp779*)	8	Nonsense	TMS4	North European	54
286	c.2341G>A (p.Glu781Lys)	8	Missense	TMS4	Chinese	7
287	c.2355+4A>G	8	Splice site	TMS4	N. European (French Canadian)	39
288	IVS9-1(G>A)	9	Splice site	TMS4	Indian	71
289	c.2356-1g>c	9	Splice site	Between TMS4 & A-domain	Chinese	7
290	c.2356-2A>G	9	Spice site	Between TMS4 & A-domain	Chinese	63
291	c.2363delC (p.Thr788Thr-fs)	9	Deletion	Between TMS4 & A-domain	Indian	71
292	c.2363C >T (p.Thr788Ile)	9	Missense	Between TMS4 & A-domain	Turkish	110
293	c.2363C>T (p.Thr788Ile)	9	Missense	Between TMS4 & A-domain	Turkish	105, 111
294	c.2364delC (p.Ser789Glnfs*18)	9	Deletion	Between TMS4 & A-domain	Indian (North)	71
295	c.2383C>T (p.Leu795Phe)	9	Missense	Between TMS4 & A-domain	Italian, Indian (West), Indian (South)	4, 28, 44
296	c.2384T>G (p.Leu795Arg)	9	Missense	Between TMS4 & A-domain	British	16
297	c.2391_2393del (p.Leu798_Tyr801delinsGlnAlaThr)	9	Deletion	Between TMS4 & A-domain	Spanish	26
298	c.2407G>A (p.Ala803Thr)	9	Missense	Between TMS4 & A-domain	Japanese	112
299	c.2424insA (p.Gly809Argfs*2)	9	Insertion	Between TMS4 & A-domain	Chinese-Han	23
300	p.Asp811Gly	9	Missense	Between TMS4 & A-domain	Chinese	30
301	c.2438_2440delinsAT (p.Leu813Tyrfs*60)	9	Deletion/Insertion	Between TMS4 & A-domain	Brazilian	8
302	c.2447+1G>T	9	Splice site	Between TMS4 & A-domain	Mediterranean	27
303	c.2447+5G>T	9	Splice site	Between TMS4 & A-domain	Chinese (Hong Kong), Italian, Chinese-Han	22, 23, 87
304	c.2447+2T>A	9	Splice site	Between TMS4 & A-domain	Dutch/German	113
305	c.2448G>T (p.Arg816Ser)	9	Missense	Between TMS4 & A-domain	Austrian	65
306	c.2448-2A>G	10	Missense	Between TMS4 & A-domain	Czech/Slovak	6
307	c.2448-1G>A	10	Missense	Between TMS4 &	Indian (North)	71

				A-domain		
308	c.2450delA (p.Glu817fs)	10	Deletion	Between TMS4 & A-domain	Egyptian	84
309	c.2463dupC (p.Met822Hisfs*32)	10	Duplication	Between TMS4 & A-domain	Korean	82
310	c.2463delC (p.Met822Trpfs*51)	10	Deletion	Between TMS4 & A-domain	Mediterranean, Italian, Mediterranean, American, Mediterranean	14, 15, 18, 36, 62
311	c.2464dupA (p.Met822Asnfs*32)	10	Duplication	Between TMS4 & A-domain	Chinese-Han	17
312	c.2480G>C (p.Arg827Pro)	10	Missence	Between TMS4 & A-domain	Italian	15
313	(p.Lys832Arg)	10	Missence	Between TMS4 & A-domain	Taiwanese	2
314	c.2497dupG (p.Val833Glyfs*21)	10	Duplication	Between TMS4 & A-domain	India (East)	11, 12
315	c.2506G>A (p.Gly836Arg)	10	Missense	A-domain	Chinese	9
316	c.2507G>A (p.Gly836Glu)	10	Missense	A-domain	Moroccan	33
317	c.2509G>T(p.Gly837*)	10	Nonsense	A-domain	Chinese	24
318	c.2510delG (p.Gly837Glufs*35)	10	Deletion	A-domain	Chinese	32
319	c.2513delA (p.Lys838Serfs*35)	10	Deletion	A-domain	Japanese, Korean	82, 101
320	c.2519C>T (p.Pro840Leu)	10	Missense	A-domain	Italian, Turkish, Hungarian, Chinese	1, 18, 24, 47, 62, 83
321	c.2527G>A (p.Gly843Arg)	10	Missense	A-domain	Moroccan	40
322	c.2524G>A (p.Asp842Asn)	10	Missense	A-domain	Chinese (Hong Kong), Chinese-Han, Indian (West)	22, 23, 28,
323	c.2532delA (p.Val845Serfs*28)	10	Deletion	A-domain	Italy, Turkey, Albania, German, Greek, Italian, Egyptian, Mediterranean, Greek, Cypriot, Polish, Bulgarian, Bosnian, Solvenian, Czech	1, 14, 15, 18, 25, 43, 47, 48, 53, 62, 70, 90, 92, 105
324	c.2549C>T (p.Thr850Ile)	10	Missense	A-domain	Chinese	9, 52
325	c.2561A>G (p.Glu854Gly)	10	Missense	A-domain	Chinese	9
326	c.2564C>A (p.Ser855Tyr)	10	Missense	A-domain	Indian (East and West), Chinese	13, 78
327	c.2570T>C (p.Ile857Thr)	10	Missense	A-domain	Italian, Turkish, French, Hungarian	14, 40, 83 83
328	c.2572A>G (p.Thr858Ala)	10	Missense	A-domain	Italian	15
329	c.2575+1G>C	10	Splice site	A-domain	British, North European	49, 50
330	c.2575+2T>C	10	Splice site	A-domain	German	56
331	c.2551delA (p.Met851Trpfs*22)	10	Deletion	A-domain	Saudi Arabian	54
332	c.2576+1G>C	10	Splice site	A-domain	British	49, 50
333	IVS11+2T>A	11	Splice site	A-domain	American	36
334	IVS11-2A>G	11	Splice site	A-domain	American	36
335	c.2581G>A (p.Ala861Thr)	11	Missense	A-domain	Chinese	38
336	c.2582_2583insG (p.Met862Hisfs*5)	11	Insertion	A-domain	Indian (North)	71
337	c.2549C>T	11	Missense	A-domain	Thai	34
338	c.2593_2594 insGTCA	11	Insertion	A-domain	Chinese	9<br>81
339	c.2191T->G (p.V864G)	11	Missense	A-domain	Sardinia	33
340	c.2605G>A (p.Gly869Arg)	11	Missense	Between A-	Chinese	9

				domain &TMS5		
341	c.2606G>T (p.Gly869Val)	11	Missense	Between A-domain & TMS5	Italian, Sardinian	18, 62, 19
342	c.2604delC (p.Gly869Glufs*4)	11	Deletion	Between A-domain & TMS5	Chinese (Hong Kong), Chinese-Han	22, 23
343	c.2605G>A (p.Gly869Arg)	11	Missense	Between A-domain & TMS5	Anglo Saxon, Spanish-Grand Canaria, Spanish, Sardinian, Chinese	4, 15, 24, 26, 80
344	c.2620 G>C (p.Ala874Pro)	11	Missense	Between A-domain & TMS5	Chinese	9, 24, 32, 78
345	c.2621C>T (p.Ala874Val)	11	Missense	Between A-domain & TMS5	Korean, Japanese, Turkish, Taiwanese, Chinese-Han, Bulgarian, Italian, Chinese	5, 9, 15, 18, 62, 70, 77, 102, 114
346	(p.Arg875Gly)	11	Missense	Between A-domain & TMS5	Taiwanese	2
347	c.2623G>A/A>G (p.Gly875Arg/p.Gly875Arg)	11	Missense	Between A-domain & TMS5	Indian (South)	115
348	c.2630_2656del (p.Ile877_Ile885del)	11	Deletion	Between A-domain & TMS5	Korean	55
349	c.2627C>T (p.Ser876Phe)	11	Missense	Between A-domain & TMS5	Unknown	116
350	c.2634T>A/G (p.Asn878Lys)	11	Missense	Between A-domain & TMS5	Egyptian	25
351	c.2648_2649del (p.Val883Alafs*3)	11	Deletion	Between A-domain & TMS5	Mediterranean, Japanese	27, 40
352	c.2650_2652del (p.Ile884del)	11	Deletion	Between A-domain & TMS5	Japanese	104
353	c.2604delC (p.Pro868fs)	11	Deletion	Between A-domain & TMS5	Chinese	117
354	c.2659delG (p.Ala887Leufs*14)	11	Deletion	Between A-domain & TMS5	Japanese, Chinese	24, 114
355	c.2660dupC (p.Thr888Tyrfs*6)	11	Duplication	Between A-domain & TMS5	Japanese	104
356	c.2662A>C (p.Thr888Pro)	11	Missense	Between A-domain & TMS5	Chinese-Han Chinese	9, 23, 24
357	c.2668G>A (p.Val890Met)	11	Missense	Between A-domain & TMS5	Greek, Chinese-Han	17, 41
358	c.2672G>A (p.Gly891Asp)	11	Missense	Between A-domain & TMS5	Korean	55
359	c.2672G>T (p.Gly891Val)	11	Missense	Between A-domain & TMS5	Italian	19
360	c.2693A>G (p.Gln898Arg)	11	Missense	Between A-domain & TMS5	British	48
361	c.2695A>T (p.Ile899Phe)	11	Missense	Between A-domain & TMS5	Italian	15
362	c.2697_2723del27 p.Ile899_Gln907del	11	Deletion	Between A-domain & TMS5	Korean, Indian (West)	28, 118
363	c.2695_2721del (p.Ile899_Gln907del)	11	Deletion	Between A-domain & TMS5	Korean, Chinese	82,116
364	c.2728A>T (p.Lys910*)	11	Nonsense	Between A-domain & TMS5	Indian (North)	71
365	c.2728A>T (p.Lys910ter)	11	Nonsense	Between A-domain & TMS5	Indian	71
366	c.2730+1G>A	11	Splice site	Between A-domain & TMS5	Chinese	17
367	IVS12+1G>A	12	Splice site	Between A-domain & TMS5	American	36
368	c.2731-2A>G	12	Splice site	Between A-domain & TMS5	British, Puerto Rican	4
369	c.2739T>A (p.Ile913Ile)	12	Silent	Between A-domain & TMS5	Taiwanese	119
370	c.2736_2746del11 (p.Ile913fs)	12	Deletion	between A-domain & TMS5	Indian (West)	28
371	c.2743C>T (p.Gln914*)	12	Nonsense	Between A-domain & TMS5	Chinese-Han	5
372	c.2752G>A (p.Asp918Asn)	12	Missense	Between A-domain & TMS5	Italian	1, 47
373	c.2753A>C (p.Asp918Ala)	12	Missense	Between A-domain & TMS5	French	40
374	c.2754C>G (p.Asp918Glu)	12	Missense	Between A-domain & TMS5	Japanese, Chinese-Han, Czech/Slovak,	5, 55, 90, 114

					Korean	
375	c.2755C>G (p.Arg919Gly)	12	Missense	Between A-domain & TMS5	Taiwanese, Chinese	2, 9
376	c.2755C>T (p.Arg919Trp)	12	Missense	Between A-domain & TMS5	Italian, Turkish, Japanese, Sardinian	1, 15, 47, 104
377	c.2761A>C (p.Ser921Arg)	12	Missense	Between A-domain & TMS5	Chinese	78
378	c.2762G>A (p.Ser921Asn)	12	Missense	Between A-domain & TMS5	Sardinian, Brazilian	1, 8, 47
370	c.2763T>A (p.Ser921Arg)	12	Missense	Between A-domain & TMS5	Chinese	10
380	c.2764_2772del (p.Gly922_Phe924del)	12	Deletion	TMS5	Japanese	104
381	c.2769T>A (p.Tyr923*)	12	Nonsense	TMS5	Chinese	54
382	c.2785 A>G (p.Ile929Val)	12	Missense	TMS5	Chinese	32
383	c.2790_2792delCAT	12	Deletion	TMS5	Chinese	81
384	c.2794_2795insGT (p.Ser932Cysfs*4)	12	Insertion	TMS5	Chinese	7
385	c.2795C>A (p.Ser932*)	12	Nonsense	TMS5	Brazlian, Spanish	8, 26
386	c.2797A>C (p.Thr933Pro)	12	Missense	TMS5	Turkish	1, 47
387	c.2804C>T (p.Thr935Met)	12	Missense	TMS5	Chinese-Han, Italian, Greek, Taiwanese, Chinese, Chinese	2, 5, 9, 15, 24, 66
388	c.2806_2808del (p.Leu936del)	12	Deletion	TMS5	Chinese	63
389	c.2807T>A (p.Leu936*)	12	Nonsense	TMS5	Greek, Saudi, Greek, Greek, Cypriot, Bulgarian	1, 47, 48, 49, 50, 70
390	c.2810delT (p.Val937Glyfs*5)	12	Deletion	TMS5	Chinese-Han, Chinese	5, 38
391	p.Leu937*	12	Nonsense	TMS5	Saudi, Greek	49, 50
392	c.2815_2816insA (p.Trp939*)	12	Nonsense	TMS5	Indian (North), Chinese	63, 71
393	c.2816G>A (p.Trp939*)	12	Nonsense	TMS5	Switzerland	33
394	c.2817G>T/C (p.Trp939Cys)	12	Missense	TMS5	Hungarian	83
395	c.2827G>A (p.Gly943Ser)	12	Missense	TMS5	Italian, Bangladeshi, Greek, Cypriot, Spanish, Korean, Sardinian	14, 26, 48, 49, 50, 55, 60, 88, 96, 98
396	c.2827G>T (p.Gly943Cys)	12	Missense	TMS5	Bangladeshi	38
397	c.2828G>A (p.Gly943Asp)	12	Missense	TMS5	Italian, Taiwanese, Chinese-Han, Chinese, Chinese (Hong Kong), Thai, Chinese	5, 9, 14, 34, 35, 59, 103
398	p.Gly944Ser	12	Missense	TMS5	Bangladeshi	49, 50
399	c.2846T>G (p.Val949Gly)	12	Missense	Between TMS5 & TMS6	Brazlian	8
400	c.2847_2849del (p.Val950del)	12	Deletion	Between TMS5 & TMS6	Saudi Arabian	54
401	c.2853_2856del (p.Gln951Hisfs*15)	12	Deletion	Between TMS5 & TMS6	Chinese	7
402	p.Arg952Lys	12	Missense	Between TMS5 & TMS6	Chinese	120
403	c.2862_2865del (p.Phe954Leufs*12)	12	Deletion	Between TMS5 & TMS6	Spanish	26
404	c.2865+1G>A	12	Splice site	Between TMS5 & TMS6	Czech/Slovak, Indian (West)	28, 90
405	c.2866-2A>G	13	Splice site	Between TMS5 & TMS6	Omani, Indian (West)	28, 121
406	c.2866-7T>C	13	Splice site	Between TMS5 & TMS6	Chinese-Han	17
407	c.2866-6T>G	13	Splice site	Between TMS5 & TMS6	Italian	69
408	c.2871delC (p.Asn958Thrfs*9)	13	Deletion	Between TMS5 & TMS6	Japanese	3
409	c.2887delC (p.Gln963Argfs*4)	13	Deletion	Between TMS5 &	Chinese-Han	17

				TMS6		
410	c.2887C>T (p.Gln963*)	13	Nonsense	Between TMS5 & TMS6	Chinese-Han	17
411	c.2899A>T (p.Ile967Phe)	13	Missense	Between TMS5 & TMS6	N European, Middle Eastern	51
412	c.2905C>T (p.Arg969Trp)	13	Missense	TMS6	Italian	15
413	c.2906G>A (p.Arg969Gln)	13	Missense	TMS6	Italian,Turkish, Libyan-Jewish, Tunisian-Jewish, Greek, German, Italian, Mediterranean, European, Bulgarian, Indian (South), Hungarian, Indian, Greek (Kalymnos)	1, 14, 18, 42, 44, 46, 47, 57, 60, 62, 70, 83, 88, 89, 122
414	c.2921C>T (p.Ala971Val)	13	Missense	TMS6	British	39
415	c.2921C>T (p.Thr974Met)	13	Missense	TMS6	Macedonian	39
416	c.2924C>A (p.Ser975Tyr)	13	Missense	TMS6	Chinese-Han, Chinese	9, 17
417	c.2930C>T (p.Thr977Met)	13	Missense	TMS6	N.European, Middle Eastern, Mediterranean, British, Swedish, Hungarian, Polish, Spanish, Indian (East), Italian, Egyptian, Czech, Indian (West)	1, 15, 16, 25, 26, 28, 47, 49, 53, 60, 75, 76, 89, 90, 96, 123
418	c.2930C > G	13	Missense	TMS6	Thai	34
419	c.2932G>A (p.Val978Met)	13	Missense	TMS6	Indian (East and West)	13
420	c.2936T>A (p.Leu979Gln)	13	Missense	TMS6	French	40
421	c.2938T>C (p.Cys980Leu)	13	Missense	TMS6	Chinese	10
422	c.2939G>A (p.Cys980Tyr)	13	Missense	TMS6	Chinese, Chinese-Han	23, 63
423	c.2945C>T (p.Ala982Val)	13	Missense	TMS6	Italian	15
424	p.Ala982Thr	13	Missense	TMS6	Chinese	124
425	c.2954G>A (p.Cys985Tyr)	13	Missense	TMS6	German	42
426	p.Ser986Phe	13	Missense	TMS6	Taiwanese	2
427	c.2960T>C (p.Leu987Pro)	13	Missense	TMS6	Chinese	10
428	c.2962G>C (p.Gly988Arg)	13	Missense	TMS6	Polish	53
429	c.2968G > C (p.Ala990Pro)	13	Missense	TMS6	Indian (West)	28
430	c.2970G>C (p.Ala990Ala)	13	Silent	Between TMS6 & ATP Binding Domain	Spanish	100
431	c.2972C>T (p.Thr991Met)	13	Missense	Between TMS6 & ATP Binding Domain	German, British, Sardinian	15, 38
432	c.2975C>T (p.Pro992Leu)	13	Missense	Between TMS6 & ATP Binding Domain	Japanese, Turkish, Taiwanese, German, Chinese-Han, Polish, Chinese (Hong Kong), Taiwanese, Korean, Thai, Chinese	1, 3, 17, 22, 24, 34, 35, 47, 53, 55, 56, 57, 60, 79, 96, 107, 119
433	c.2975C>A (p.Pro992His)	13	Missense	Between TMS6 & ATP Binding Domain	Indian (North)	71
434	c.2977dupA (p.Thr993Asnfs*35)	13	Duplication	Between TMS6 & ATP Binding Domain	Indian (North), Indian	71, 125
435	c.2978C>T (p.Thr993Met)	13	Missense	Between TMS6 & ATP Binding Domain	Sardinian	88
436	c.2984T>C (p.Val995Ala)	13	Missense	Between TMS6 & ATP Binding Domain	Unknown	60
437	c.2986_3008delinsTATGTGG (p.Met996Tyrfs*21)	13	Deletion/Insertion	Between TMS6 & ATP Binding	Polish	53

				Domain		
438	c.2987T>C (p.Met996Thr)	13	Missense	Between TMS6 & ATP Binding Domain	Scottish	38
439	c.2993G>A (p.Gly998Asp)	13	Missense	Between TMS6 & ATP Binding Domain	Egyptian	25
440	c.2997dupC (p.Gly1000Argfs*28)	13	Duplication	Between TMS6 & ATP Binding Domain	Yemeni, Egyptian	16, 25
441	c.2998G>A (p.Gly1000Arg)	13	Missense	Between TMS6 & ATP Binding Domain	British, Sardinian	38, 88
442	c.3007G>A (p.Ala1003Thr)	13	Missense	Between TMS6 & ATP Binding Domain	Italian, Japanese, Greek, Cypriot, Mediterranean, Indian (North), Bulgarian, Indian, Indian (West)	1, 27, 28, 47, 48, 70, 71, 104, 126
443	c.3008C>T (p.Ala1003Val)	13	Missense	Between TMS6 & ATP Binding Domain	Turkish, Indian (South)	18, 44, 62
444	c.3010C>T (p.Gln1004*)	13	Nonsense	Between TMS6 & ATP Binding Domain	Chinese	7
445	c.3011A>C (p.Gln1004Pro)	13	Missense	Between TMS6 & ATP Binding Domain	European	39
446	c.3026_3028del (p.Ile1009del)	13	Deletion	Between TMS6 & ATP Binding Domain	Indian	116
447	c. 3028A>G (p.Lys1010Glu)	13	Missense	Between TMS6 & ATP Binding Domain	Chinese	127
448	c.3029A>G (p.Lys1010Arg)	13	Missense	Between TMS6 & ATP Binding Domain	Indian (South)	44
449	c.3029A>C (p.Lys1010Thr)	13	Missense	Between TMS6 & ATP Binding Domain	Japanese	104
450	c.3029_3030insT (p.Lys1010Nfs*18)	13	Insertion	Between TMS6 & ATP Binding Domain	Chinese-Han	17
451	c.3031_3032insC (p.Gly1011Alafs*17)	13	Insertion	Between TMS6 & ATP Binding Domain	Indian (North)	71
452	c.3034G>C (p.Gly1012Arg)	13	Missense	Between TMS6 & ATP Binding Domain	Greek	15
453	c.3035G>T (p.Gly1012Val)	13	Missense	Between TMS6 & ATP Binding Domain	Italian	15
454	c.3053C>T (p.Ala1018Val)	13	Missense	Between TMS6 & ATP Binding Domain	Sardinian, Czech, Italian, Chinese-Han Sardinian	1, 15, 23, 47, 88, 90
455	c.3056A>C (p.His1019Pro)	13	Missense	Between TMS6 & ATP Binding Domain	Chinese	9
456	c.3060+5G>T	13	Splice site	Between TMS6 & ATP Binding Domain	Spanish	26
457	c.3061-3C>A	13	Splice site	Between TMS6 & ATP Binding Domain	Chinese	9
458	c.2963G>T (p.Gly988Val)	13	Missense	ATP Binding Domain	Chinese (Han)	30
459	c.3041 C>T (p.Pro1014Leu)	13	Missense	ATP Binding Domain	Chinese	32
460	c.3061G >A (p.Lys1020Lys)	13	Missense	ATP Binding Domain	Iranian	105
461	c.3061-12T>A	14	Splice site	ATP Binding Domain	Spanish-Grand Canaria, Spanish	69, 80
462	c.3061-3C>A	14	Splice site	ATP Binding Domain	Chinese-Han	23
463	c.3061-1G>A	14	Splice site	ATP Binding Domain	Mediterranean	19
464	c.3062T>A (p.Ile1021Lys)	14	Missense	ATP Binding	Danish	72

				Domain		
465	c.3071T>C (p.Val1024Ala)	14	Missense	ATP Binding Domain	Korean	55
466	c.3074T>G (p.Met1025Arg)	14	Missense	ATP Binding Domain	Chinese-Han	23
467	c.3080A>C (p.Asp1027Ala)	14	Missense	ATP Binding Domain	Unknown	128
468	c.3082_3085delinsCG (p.Lys1028Argfs*40)	14	Deletion	ATP Binding Domain	North European (French Canadian)	39
469	c.3083_3084del (p.Lys1028Asnfs*40)	14	Deletion	ATP Binding Domain	British, French	16, 40
470	c.3083_3085delinsG (p.Lys1028Serfs*40)	14	Deletion	ATP Binding Domain	Italian, N. European, Middle Eastern	14, 51
471	c.3085A>G (p.Thr1029Ala)	14	Missense	ATP Binding Domain	Unknown	128
472	c.3085_3086del (p.Thr1029Trpfs*39)	14	Deletion	ATP Binding Domain	British, N. European	49, 50
473	c.3086C>T (p.Thr1029Ile)	14	Missense	ATP Binding Domain	Korean, Japanese	55, 101
474	c.3087delT (p.Gly1030Alafs*91)	14	Deletion	ATP Binding Domain	Chinese	68
475	c.3088delAC	14	Deletion	ATP Binding Domain	British	49, 50
476	c.3089G>A (p.Gly1030Asp)	14	Missense	ATP Binding Domain	Indian (West), Chinese	28, 30
477	c.3091A>T (p.Thr1031Ser)	14	Missense	ATP Binding Domain	German	43
478	c.3091A>G (p.Thr1031Ala)	14	Missense	ATP Binding Domain	Korean, Indian (East)	55, 75, 76
479	c.3097A>G (p.Thr1033Ala)	14	Missense	ATP Binding Domain	Pakistani	16
480	c.3098C>G (p.Thr1033Ser)	14	Missense	ATP Binding Domain	Czech/Slovak	6
481	c.3098C>T (p.Thr1033Ile)	14	Missense	ATP Binding Domain	Chinese	7
482	c.3104G>T (p.Gly1035Val)	14	Missense	ATP Binding Domain	Japanese, Korean	3, 55
483	c.3106G >A (p.Val1036Ile)	14	Missense	ATP Binding Domain	Turkish	110
484	c.3111delC (p.Arg1038Gly-fs*83)	14	Missense	ATP Binding Domain	Turkish	110
485	c.3113G>A (p.Arg1038Lys)	14	Missense	ATP Binding Domain	Finnish	67
486	c.3121C>T (p.Arg1041Trp)	14	Missense	ATP Binding Domain	Italian, American, Taiwanese	1, 2, 8, 36, 47
487	c.3122G>C (p.Arg1041Pro)	14	Missense	ATP Binding Domain	Italian, Chinese-Han	5, 18, 62
488	c.3128T>C (p.Leu1043Pro)	14	Missense	ATP Binding Domain	Sardinian, British, American	16, 19, 36
489	c.3140delA (p.Asp1047Valfs*75)	14	Deletion	ATP Binding Domain	Czech/Slovak, Polish, Chinese-Han Chinese	6, 23, 24, 53
490	c.3140A>T (p.Asp1047Val)	14	Missense	ATP Binding Domain	Chinese-Han Chinese	23, 117
491	c.3147delC (p.Thr1050Hisfs*71)	14	Deletion	ATP Binding Domain	Indian, Indian (East), Indian (West)	28, 49, 50
492	c.3155C>T (p.Pro1052Leu)	14	Missense	ATP Binding Domain	British	16
493	c.3182G>A (p.Gly1061Glu)	14	Missense	ATP Binding Domain	Pakistani, Turkish, Greek, Russian, Indian (South), Spanish, Indian, Hungarian, Italian, Indian (West)	11, 15, 16, 18, 26, 28, 41, 44, 62, 83, 129, 130
494	c.3191A>C (p.Glu1064Ala)	14	Missense	ATP Binding Domain	Jewish, Ashkenazi Jewish, American	4, 36, 46, 60, 131
495	p.Glu1064Lys	14	Missense	ATP Binding Domain	American	36
496	c.3193G>C (p.Ala1065Pro)	14	Missense	ATP Binding Domain	Spanish, European	100, 132
497	c.3203A>G (p.Glu1068Gly)	14	Missense	ATP Binding Domain	Italian	18, 62
498	c.3206C> A (p.His1069Asn)	14	Missense	ATP Binding Domain	Iranian	105
499	c.3207C>A (p.His1069Gln)	14	Missense	ATP Binding	Russian, N. American,	1, 4, 14, 16,

				Domain	Italian, Turkish, Albanian, Dutch, Polish, Swedish, Ukranian, Greek, German, French, British, N. European, N. European, Middle Eastern, Polish, Austrian, Polish, Swedish, German, Cuban, Ashkenazi Jewish, Moroccan-Jewish, Greek, French, Italian, British, Hungarian, German, Russian, Italian, Mediterranean, Sardinian, Swedish, British, Hungarian, Czech, Spanish, Bulgarian, French, Egyptian, Brazlian, Greek (Kalymnos), Dutch/German	18, 25, 26, 40, 42, 43, 46, 47, 48, 49, 50, 51, 57, 60, 62, 70, 88, 89, 90, 97, 113, 123, 128, 129, 131, 133, 134, 135, 136, 137, 138, 139
500	c.3106G>A (p.Val1036Ile)	14	Missense	ATP Binding Domain	Turkish	111
501	c.3111delC (p.Arg1038Glyfs*83)	14	Deletion	ATP Binding Domain	Turkish	111
502	c.3149delC	14	Deletion	ATP Binding Domain	Indian	49, 50
503	c.3157dupC (p.Leu1053Profs*16)	14	Duplication	ATP Binding Domain	Chinese, Indian (West)	28, 32
504	c.3188C>T (p.Ala1063Val)	14	Missense	ATP Binding Domain	Egyptian	84
505	p.His1070Gln	14	Missense	ATP Binding Domain	Eastern European, German, French, British	133
506	p.Thr1076Ile	14	Missense	ATP Binding Domain	Egyptian	140
507	c.3236G>T (p.Cys1079Phe)	14	Missense	ATP Binding Domain	Chinese	78
508	c.3243+5G>A	15	Splice site	ATP Binding Domain	Indian (West)	28
509	c.3247C>T (p.Leu1083Phe)	15	Missense	ATP Binding Domain	Korean, Japanese, Unknown	77, 97, 130
510	c.3244G>T (p.Glu1082*)	15	Nonsense	ATP Binding Domain	Italy	33
511	c.3244-2A>G	15	Splice-site	ATP Binding Domain	Italy, Chinese	33, 78
512	c.3263T>A (p.Leu1088*)	15	Nonsense	ATP Binding Domain	Czech/Slovak , Brazlian	6, 8
513	c.3266G>A (p.Gly1089Glu)	15	Missense	ATP Binding Domain	Turkish	19
514	c.3266G>T (p.Gly1089Val)	15	Missense	ATP Binding Domain	Sardinian	1, 47
515	c.3272G>A (p.Cys1091Tyr)	15	Missense	ATP Binding Domain	Korean	55
516	c.3282C>G (p.Phe1094Leu)	15	Missense	ATP Binding Domain	Brazlian, Indian (South)	8
517	c.3284A>C (p.Gln1095Pro)	15	Missense	ATP Binding Domain	British, Polish	16, 53
518	c.3293C>G (p.Pro1098Arg)	15	Missense	ATP Binding Domain	Chinese-Han	17
519	c.3295G>A (p.Gly1099Ser)	15	Missense	ATP Binding Domain	Greek, Spanish, Italian	15, 26, 41
520	c.3301G>A (p.Gly1101Arg)	15	Missense	ATP Binding Domain	Indian, Indian (west)	28, 49, 50
521	c.3044T>C (p.Leu1015Pro)	15	Missense	ATP Binding Domain	Chinese	86
522	c.3305T>C (p.Ile1102Thr)	15	Missense	ATP Binding Domain	Indian, Greek, Cypriot, Arabic, Mediterranean, Czech, Indian (East), Indian (west)	27, 28, 48, 49, 50, 75, 76

523	c.3309delG (p.Cys1104Alafs*17)	15	Deletion	ATP Binding Domain	Chinese-Han	17
524	c.3310T>A (p.Cys1104Ser)	15	Deletion	ATP Binding Domain	Indian	141
525	c.3311G>T (p.Cys1104Phe)	15	Missense	ATP Binding Domain	Turkish	18, 62
526	c.3311G>A (p.Cys1104Tyr)	15	Missense	ATP Binding Domain	Indian (North)	71
527	c.3316G>A (p.Val1106Ile)	15	Missense	ATP Binding Domain	Chinese	59
528	c.3317T>A (p.Val1106Asp)	15	Missense	ATP Binding Domain	North European, Middle Eastern	51, 130
529	c.3332G>A (p.Gly1111Asp)	15	Missense	ATP Binding Domain	Czech/Slovak	6, 90
530	c.3337C>A (p.Leu1113Met)	15	Missense	ATP Binding Domain	Indian	141
531	c.3344A>G/G>A (p.Arg1115His/p.His1115Arg)	15	Missence	ATP Binding Domain	Unknown	60
532	c.3350_3353del (p.Glu1117Alafs*3)	15	Deletion	ATP Binding Domain	Polish	53
533	c.3359T>A (p.Leu1120*)	15	Nonsense	ATP Binding Domain	Spanish	26
534	c.3365_3366ins11	15	Insertion	ATP Binding Domain	Italian	15
535	c.3373_3377delinsTCT (p.His1126Profs*3)	15	Deletion/Insertion	ATP Binding Domain	Egyptian	25
536	c.3376delC (p.His1126Thrfs*2)	15	Deletion	ATP Binding Domain	Italian, Russian, N. American, Ukranian, N. European, Middle Eastern, German, Cuban, French, Portugese, British, German, Spanish-Grand Canaria, Hungarian, Mediterranean, Chinese-Han, Brazlian, Polish, Spanish, Bulgarian, Brazlian	8, 14, 16, 17, 26, 27, 42, 43, 49, 50, 51, 53, 57, 70, 80, 89, 97, 129, 139
537	c.3373_3377delAGTCAinsTC (p.His1126fs)	15	Deletion-Insertion	ATP Binding Domain	Egyptian	84
538	c.3310 T>C (p.Cys1104Arg)	15	Missense	ATP Binding Domain	Chinese	32
539	c.G>A3311 (p.Cys1104Thr)	15	Missence	ATP Binding Domain	Mediterranean	142
540	c.3377_3378 delAC (p.His1126Profs*3)	15	Deletion	ATP Binding Domain	Chinese	9
541	c.3402delC (p.Ala1135Glnfs*13)	15	Deletion	ATP Binding Domain	Venezuelan	143
542	c.3403delC	15	Deletion	ATP Binding Domain	Ukrainian	133
543	c.3406 G>A	15	Unknown	ATP Binding Domain	Chinese	58
544	c.3412+1G>A	15	Splice site	ATP Binding Domain	Indian (East)	75, 76
545	c.3418delT (p.Val1140Ala-fs)	16	Deletion	ATP Binding Domain	Indian	71
546	c.3419C>T (p.Ala1140Val)	16	Missense	ATP Binding Domain	Chinese	59
547	c.3419delT (p.Gln1142Argfs*6)	16	Deletion	ATP Binding Domain	Indian (North)	71
548	c.3424C>T(p.Gln1142*)	16	Nonsense	ATP Binding Domain	Italian, American	18, 36, 62
549	c.3424dupC (p.Gln1142Profs*11)	16	Duplication	ATP Binding Domain	Indian (North)	71
550	c.3426G>C (p.Gln1142His)	16	Missense	ATP Binding Domain	Taiwanese, Chinese-Han	5, 23, 35
551	c.3436G>A (p.Val1146Met)	16	Missense	ATP Binding Domain	Sardinian, Mediterranean	1, 18, 47, 62

552	c.3443T>C (p.Ile1148Thr)	16	Missense	ATP Binding Domain	Greek, German, Chinese-Han, Greek (Crete), Korean, Egyptian, Thai, Chinese	1, 17, 24, 25, 29, 34, 42, 47, 55, 90
553	c.3446G>C (p.Gly1149Ala)	16	Missense	ATP Binding Domain	Filipino	39
554	c.3446G>A (p.Gly1149Glu)	16	Missense	ATP Binding Domain	Chinese	32, 58, 78
555	c.3449delA (p.Asn1150Thrfs*5)	16	Deletion	ATP Binding Domain	British, Brazilian	8, 16
556	c.3451C>T (p.Arg1151Cys)	16	Missense	ATP Binding Domain	Sardinian, Turkish, Chinese	7, 15, 110, 111
557	c.3452G>A (p.Arg1151His)	16	Missense	ATP Binding Domain	Turkish	18, 62
558	c.3457T>C (p.Trp1153Arg)	16	Missense	ATP Binding Domain	N. European, Middle Eastern	51
559	c.3458G>A (p.Trp1153*)	16	Missense	ATP Binding Domain	Indian (West)	28
560	c.3459G>T (p.Trp1153Cys)	16	Missense	ATP Binding Domain	Taiwanese	35
561	c.3467G>A (p.Arg1156His)	16	Missense	ATP Binding Domain	Chinese-Han	23
562	c.3472_3482del (p.Gly1158Phe*2)	16	Deletion	ATP Binding Domain	Polish	4
563	c.3473G>T (p.Gly1158Val)	16	Missense	ATP Binding Domain	Danish	72
564	c.3490G>A (p.Asp1164Asn)	16	Missense	ATP Binding Domain	Italian	39
565	c.3502G>T (p.Ala1168Ser)	16	Missense	ATP Binding Domain	Korean	82
566	c.3502G>C (p.Ala1168Pro)	16	Missense	ATP Binding Domain	Taiwanese	119
567	c.3505A>G (p.Met1169Val)	16	Missense	ATP Binding Domain	Unknown	130
568	c.3506T>C (p.Met1169Thr)	16	Missense	ATP Binding Domain	Turkish	18, 62
569	c.3517G>A (p.Glu1173Lys)	16	Missense	ATP Binding Domain	Italian, Taiwanese, Chinese-Han, French, Chinese	5, 18, 24, 40, 62, 102
570	c.3518A>G (p.Glu1173Gly)	16	Missense	ATP Binding Domain	Irish	39
571	c.3526G>A (p.Gly1176Arg)	16	Missense	ATP Binding Domain	German, Greek, Cypriot, Czech, Greek (Crete)	29, 43, 48, 90
572	c.3527G>A (p.Gly1176Glu)	16	Missense	ATP Binding Domain	Scottish	38
573	c.3532A>G (p.Thr1178Ala)	16	Missense	ATP Binding Domain	Taiwanese, Indian (East), Chinese-Han Thai	23, 34, 75, 76, 119
574	c.3538delA (p.Ile1180Serfs*12)	16	Deletion	ATP Binding Domain	Japanese	101
575	c.3547G>A (p.Ala1183Thr)	16	Missense	ATP Binding Domain	Italian	1, 47
576	c.3548C>G (p.Ala1183Gly)	16	Missense	ATP Binding	Italian	69

				Domain		
577	c.3551T>C (p.Ile1184Thr)	16	Missense	ATP Binding Domain	Polish	54
578	c.3552dupT (p.Asp1185*)	16	Nonsense	ATP Binding Domain	N. American	133
579	c.3556G>A (p.Gly1186Ser)	16	Missense	ATP Binding Domain	Japanese, Korean, Indian (Western and eastern)	13, 55, 114
580	c.3556G>T (p.Gly1186Cys)	16	Missense	ATP Binding Domain	Jewish	4
581	c.3556+1G>A	16	Splice site	ATP Binding Domain	British, North European	49, 50
582	c.3556+1G>T	16	Splice site	ATP Binding Domain	Bashkortostan	129
583	c.3557-2A>G	16	Splice site	ATP Binding Domain	Chinese	7
584	c.3559+1G>A	17	Splice site	ATP Binding Domain	British	49, 50
585	c.3565_3566delTG	17	Deletion	ATP Binding Domain	Thai	34
586	c.3577G>C (p.Ala1193Pro)	17	Missense	ATP Binding Domain	Taiwanese	119
587	c.3584C>T (p.Ala1195Val)	17	Missense	ATP Binding Domain	Chinese	9
588	c.3588dupC (p.Ala1197Argfs*62)	17	Duplication	ATP Binding Domain	N. European, Middle Eastern	51
589	c.3598C>T (p.Gln1200*)	17	Nonsense	ATP Binding Domain	French	40
590	c.3599A>C (p.Gln1200Pro)	17	Missense	ATP Binding Domain	French	97
591	c.3620A>G (p.His1207Arg)	17	Missense	ATP Binding Domain	Egyptian	25
592	c.3627_3630del (p.Gln1210Alafs*8)	17	Deletion	ATP Binding Domain	Swedish, North European, Middle Eastern	49, 50, 51
593	c.3627_3632del (p.Gln1210_Ser1211del)	17	Deletion	ATP Binding Domain	German	37
594	c.3629del4	17	Deletion	ATP Binding Domain	Swedish	49, 50
595	c.3638G>T (p.Gly1213Val)	17	Missense	ATP Binding Domain	Kurdish-Jewish	46
596	p.Gly1213Ser	17	Missense	ATP Binding Domain	American	36
597	c.3643G>T (p.Asp1215Tyr)	17	Missense	ATP Binding Domain	Japanese	144, 145
598	c.3644A>G (p.Asp1215Gly)	17	Missense	ATP Binding Domain	Indian (Eastern and Western)	13
599	c.3646G>A (p.Val1216Met)	17	Missense	ATP Binding Domain	Turkish, German, Taiwanese, Chinese, Spanish, Korean, Chinese-Han	1, 23, 26, 42, 46, 47, 55, 59,102
600	c.3649_3654del (p.Val1217_Leu1218del)	17	Deletion	ATP Binding Domain	British, Finnish, Druze-Israel, Mediterranean, Bulgarian, Hungarian, Bulgarian	1, 47, 49, 50, 67, 70, 83
601	c.3659C>T (p.Thr1220Met)	17	Missense	ATP Binding Domain	Turkish, German, Polish, Italian, Egyptian	15, 25, 42, 53, 19
602	c.3661_3663delGGG	17	Deletion	ATP Binding Domain	Chinese	81
603	c.3662G>A (p.Gly1221Glu)	17	Missense	ATP Binding	British	38

				Domain		
604	c.3664G>A (p.Asp1222Asn)	17	Missense	ATP Binding Domain	Japanese	101
605	c.3664G>T (p.Asp1222Tyr)	17	Missense	ATP Binding Domain	Russian	4
606	c.3665A>T (p.Asp1222Val)	17	Missense	ATP Binding Domain	Turkish	18, 62
607	c.3677C>T (p.Thr1266Ile)	17	Missense	ATP Binding Domain	Chinese	9
608	c.3679G>C (p.Ala1227Pro)	17	Missense	ATP Binding Domain	Chinese	9
609	c.3682A>T (p.Arg1228*)	17	Nonsense	ATP Binding Domain	Chinese	78
610	c.3683G>C (p.Arg1228Thr)	17	Missense	ATP Binding Domain	Lebanese	39
611	c.3688A>G (p.Ile1230Val)	17	Missense	ATP Binding Domain	European	39
612	c.3693-3697delins19bp	17	Deletion/Insertion	ATP Binding Domain	American	36
613	c.3694A>C (p.Thr1232Pro)	17	Missense	ATP Binding Domain	Brazilian, Spanish	8, 26
614	c.3699+1G>C	17	Splice site	ATP Binding Domain	Spanish	26
615	c.3699+27T>C	17	Splice site	ATP Binding Domain	Mediterranean	18, 49
616	c.3700delG	17	Spice site	ATP Binding Domain	Chinese	9, 81
617	c.3700-2A>T	18	Splice site	ATP Binding Domain	Taiwanese	35, 102
618	c.3700-1G>A	18	Splice site	ATP Binding Domain	Chinese-Han	23
619	c.3700-1delG	18	Splice site	ATP Binding Domain	Chinese-Han	17
620	c.3707T>C (p.Ile1236Thr)	18	Missense	ATP Binding Domain	Turkish (Bulgaria)	70
621	c.3713_3714del (p. Lys1238Serfs*20)	18	Deletion	ATP Binding Domain	North European, Brazilian, Middle Eastern,	8, 51, 139
622	c.3715G>T (p.Val1239Phe)	18	Missense	ATP Binding Domain	Chinese	7
623	c.3716T>G (p.Val1239Gly)	18	Missense	ATP Binding Domain	British	16
624	c.3722C>T (p.Ala1241Val)	18	Missense	ATP Binding Domain	Indian (South)	44
625	c.3731delT (p.Leu1244Argfs*86)	18	Deletion	ATP Binding Domain	Egyptian	25
626	c.3733C >A (p.Pro1245Thr)	18	Deletion	ATP Binding Domain	Turkish	110, 111
627	c.3734G>T (p.1245Leu)	18	Missense	ATP Binding Domain	Egyptian	84
628	c.3741C>G (p.His1247Gln)	18	Missense	ATP Binding	Indian (Eastern and Western)	13

				Domain		
629	c.3742_3743dup (p.Lys1248Thrfs*83)	18	Duplication	ATP Binding Domain	Italian	15
630	c.3744G>C (p.Lys1248Asn)	18	Missense	ATP Binding Domain	Chinese-Han	17
631	c.3754G>A (p.Val1252Ile)	18	Missense	ATP Binding Domain	European	132
632	c.3763C>A (p.Leu1255Ile)	18	Missense	ATP Binding Domain	Korean	82
633	c.3765insAC	18	Insertion	ATP Binding Domain	Chinese	10
634	c.3767insCA	18	Insertion	ATP Binding Domain	Chinese	58
635	c.3766_3767dupCA (p.Gln1256Profs*75)	18	Duplication	ATP Binding Domain	Chinese	7
636	c.3767A>G (p.Gln1256Arg)	18	Missense	ATP Binding Domain	Indian (North), Indian	71, 126
637	c.3770_3771insG (p.Asn1257Lysfs*2)	18	Insertion	ATP Binding Domain	Indian (North)	71
638	c.3772A>T (p.Lys1258*)	18	Nonsense	ATP Binding Domain	Brazlian	8
639	c.3784G>T (p.Val1262Phe)	18	Missense	ATP Binding Domain	Italian	18, 62
640	c.3794_3803del (p.Val1265Glyfs*62)	18	Deletion	ATP Binding Domain	Czech/Slovak	6
641	c.3796G>A (p.Gly1266Arg)	18	Missense	ATP Binding Domain	British, Hungarian, American, Chinese	16, 32, 36
642	c.3796_3797GG>AA (p.Gly1266Lys)	18	Missense	ATP Binding Domain	French, British, German	49, 50
643	c.3797G>A (p.Gly1266Glu)	18	Missense	ATP Binding Domain	Chinese	10
644	c.3797G>T (p.Gly1266Val)	18	Missense	ATP Binding Domain	American	4
645	c.3799 G>A (p.Asp1267Asn)	18	Missense	ATP Binding Domain	Chinese	32
646	c.3800A>C (p.Asp1267Ala)	18	Missense	ATP Binding Domain	Japanese, Korean	82, 114, 138
647	c.3800A>G (p.Asp1267Gly)	18	Missense	ATP Binding Domain	Latvian	146
648	c.3800A>T (p.Asp1267Val)	18	Missense	ATP Binding Domain	Japanese	39
649	c.3802G>A (p.Gly1268Arg)	18	Missense	ATP Binding Domain	Chinese-Han	147
650	c.3809A>C (p.Asn1270Thr)	18	Missense	ATP Binding Domain	Bulgarian, Indian (West)	28
651	c.3809A>G (p.Asn1270Thr)	18	Missense	ATP Binding Domain	Sicilian, Italian, Turkish, Italian, Costa Rican, Japanese, Korean, French, Spanish-Grand Canaria, Taiwanese, Japanese, German, Chinese-Han, Korean, Bulgarian, Brazilian Korean, Indian	4, 5, 8, 14, 23, 25, 35, 36, 44, 49, 50, 56, 59, 67, 80, 82, 97, 102, 104, 114, 119, 128, 133, 147

					(South), Taiwanese, Chinese-Han, Egyptian, Chinese-Han, Bulgarian, American	
652	c.3809A>G (p.Asn1270Ser)	18	Missense	ATP Binding Domain	Czech/Slovak	6
653	c.3817C>T (p.Pro1273Leu)	18	Missense	ATP Binding Domain	Turkish, Polish, Hungarian, Korean, Hungarian, Egyptian, Chinese - Han	19, 23, 25, 53, 55, 83, 138
654	c.3818C>T (p.Pro1273Leu)	18	Missense	ATP Binding Domain	Egyptian Indian (West)	28, 84
655	p.1273Ser	18	Missense	ATP Binding Domain	American	36
656	c.3818C>A (p.Pro1273Gln)	18	Missense	ATP Binding Domain	Taiwanese, Egyptian, Chinese-Han, Thai	23, 25, 34, 119
657	c.3829C>T (p.Gln1277*)	18	Nonsense	ATP Binding Domain	Turkish (Bulgarian)	73
658	c.3832G>A (p.Ala1278Thr)	18	Missense	ATP Binding Domain	Indian (Eastern and Western)	13
659	c.3833C>T (p.Ala1278Val)	18	Missense	ATP Binding Domain	Sardinian, American	36, 148
660	c.3835G>T (p.Asp1279Tyr)	18	Missense	ATP Binding Domain	Bulgarian	70
661	c.3836A>G (p.Asp1279Gly)	18	Missense	ATP Binding Domain	Taiwanese	102
662	c.3839_3840insTAC (p.Met1280delinsIleThr)	18	Insertion	ATP Binding Domain	Indian (North)	71
663	c.3839insTAC (p.Met1280Ile)	18	Insertion	ATP Binding Domain	Indian	71
664	c.3841G>T (p.Gly1281Cys)	18	Missense	ATP Binding Domain	Chinese-Han	23
665	c.3842G>A (p.Gly1281Asp)	18	Missense	ATP Binding Domain	Hungarian, American	36, 83
666	c.3843insT	18	Insertion	ATP Binding Domain	American,Chinese	9, 36
667	c.3843dupT (p.Val1282Cysfs*22)	18	Duplication	ATP Binding Domain	Chinese	32
668	c.3851_3876del	18	Deletion	ATP Binding Domain	Chinese	52
669	c.3852_3875del (p.Gly1285_Ile1292del)	18	Deletion	ATP Binding Domain	Sardinian	14
670	c.3859G>A (p.Gly1287Ser)	18	Missense	ATP Binding Domain	British	38
671	c.3863C>G (p.Thr1288Arg)	18	Missense	ATP Binding Domain	Sicilian	149
672	c.3863C>T (p.Thr1288Met)	18	Missense	ATP Binding Domain	Italian	15
673	c.3871G>A (p.Ala1291Thr)	18	Missense	ATP Binding Domain	Chinese	81

674	c.3877G>A (p.Glu1293Lys)	18	Missense	ATP Binding Domain	Chinese	33
675	c.3884C>T (p.Ala1295Val)	18	Missense	ATP Binding Domain	Chinese	24
676	c.3886G>A (p.Asp1296Asn)	18	Missense	Between ATP Binding Domain & TMS7	Japanese	150
677	c.3889 G>A (p.Val1297Ile)	18	Missense	Between ATP Binding Domain & TMS7	Chinese	32
678	c.3890T>A (p.Val1297Asp)	18	Missense	Between ATP Binding Domain & TMS7	Indian (South)	44
679	c.3892_3894del (p.Val1298del)	18	Deletion	Between ATP Binding Domain & TMS7	Japanese	151
680	c.3895C>T (p.Leu1299Phe)	18	Missense	Between ATP Binding Domain & TMS7	Indian (South), Indian (West)	28, 44
681	c.3895delC (p.Ile1300Serfs*30)	18	Deletion	Between ATP Binding Domain & TMS7	Indian (South)	44
682	c.3896T>G (p.Leu1299Arg)	18	Missense	Between ATP Binding Domain & TMS7	Chinese	7
683	c.3901_3902insA p.Arg1301Lysfs*3	18	Insertion	Between ATP Binding Domain & TMS7	Chinese	7
684	c.3901_3902 insA	18	Insertion	Between ATP Binding Domain & TMS7	Chinese	9
685	c.3903+1delG	18	Splice site	Between ATP Binding Domain & TMS7	German	43
686	c.3903+6T>C	18	Splice site	Between ATP Binding Domain & TMS7	Indian (South)	44
687	c.3903+5G>A	18	Splice site	Between ATP Binding Domain & TMS7	Chinese	7
688	c.3904-2A>G	19	Splice site	Between ATP Binding Domain & TMS7	Italian, Sicilian, Greek, Egyptian	1, 4, 14, 25, 41, 47
689	c.3912G>T (p.Leu1304Phe)	19	Missense	Between ATP Binding Domain & TMS7	Danish	72
690	c.3928A>C (p.Ser1310Arg)	19	Missense	Between ATP Binding Domain & TMS7	Italian	19
691	c.3955C>T (p.Arg1319stop)	19	Nonsense	Between ATP Binding Domain & TMS7	British, Italian, North European, Middle Eastern, British, Mediterranean, Sicilian, Spanish, Brazilian, Egyptian, Chinese- Han, American	4, 8, 14, 18, 23, 25, 26, 36, 49, 50, 51, 62, 139, 152,
692	c.3960G>C (p.Arg1320Ser)	19	Missense	Between ATP Binding Domain & TMS7	Chinese-Han, Anglo Saxon, Italian, Chinese	23, 24

693	p.Arg1320*	19	Nonsense	Between ATP Binding Domain & TMS7	British	49, 50
694	c.3965G>C (p.Arg1322Pro)	19	Missense	Between ATP Binding Domain & TMS7	Anglo Saxon, Italian	4, 15
695	c.3971A>C (p.Asn1324Thr)	19	Missense	TMS7	Latvian	146
696	c.3973dupC (p.Leu1325Profs*8)	19	Duplication	TMS7	Greek, Cypriot	48
697	c.3979C>G (p.Leu1327Val)	19	Missense	TMS7	Turkish	18, 62
698	c.3982G>A (p.Ala1328Thr)	19	Missense	TMS7	Taiwanese	39
699	c.3992A>C (p.Tyr1331Ser)	19	Missense	TMS7	Irish	38
700	c.3992T>G (p.Tyr1331*)	19	Missense	TMS7	Chinese	127
701	c.3994A>G (p.Asn1332Asp)	19	Missense	TMS7	Egyptian	25
702	c.3996C>A (p.Asn1332Lys)	19	Missense	TMS7	Italian	15
703	c.4001T>A (p.Val1334Asp)	19	Missense	TMS7	Chinese-Han	23
704	c.4003 G>C (p.Gly1335Arg)	19	Missense	TMS7	Chinese	32
705	c.4006delA	19	Missense	TMS7	Chinese	10
706	c.4007T>C (p.Ile1336Thr)	19	Missense	TMS7	Japanese	97
707	c.4021G>A (p.Gly1341Ser)	19	Missense	TMS7	Saudi Arabian, Indian (South), Indian (West)	28, 44, 88, 153, 154
708	c.4021G>C (p.Gly1341Arg)	19	Missense	TMS7	Egyptian	25
709	c.4021+3A>G	19	Splice site	TMS7	Indian (South)	44
710	c.4021+87_4125-1del	19	Deletion	TMS7	Turkish	155
711	c.4022G>T (p.Gly1341Val)	20	Missense	TMS7	Ukranian, Hungarian	38, 83
712	c.4022G>A (p.Gly1341Asp)	20	Missense	TMS7	Egyptian	84
713	p.Ile1348Asn	20	Missense	TMS7	Taiwanese	2
714	c.4051C>T (p.Gln1351*)	20	Nonsense	TMS8	European (German, Polish), Polish, German, Hungarian	53, 83, 94, 156
715	c.4054C>T (p.Pro1352Ser)	20	Missense	TMS8	Bulgarian	70
716	c.4055C>T (p.Pro1352Leu)	20	Missense	TMS8	Bulgarian	70
717	c.4055C>G (p.Pro1352Arg)	20	Missense	TMS8	Italian	15
718	c.4057T>C/A (p.Trp1353Arg)	20	Missense	TMS8	Italian	4
719	c.4058G>A (p.Trp1353*)	20	Nonsense	TMS8	British	16
720	c.4059G>A (p.Trp1353*)	20	Nonsense	TMS8	Chinese	7
721	p.Gly1355Asp	20	Missense	TMS8	Taiwanese	2
722	c.4063G>A (p.Gly1355Ser)	20	Missense	TMS8	N. European, Middle Eastern, Czech/Slovak	6, 51
723	c.4070C>T (p.Ala1357Val)	20	Missense	TMS8	Indian (West)	28
724	c.4072G>T(p.Ala1358Ser)	20	Missense	TMS8	Italian	1, 47
725	c.4077G>C (p.Met1359Ile)	20	Missense	TMS8	British	39
726	c.4085_4086del (p.Ser1362Phefs*15)	20	Deletion	TMS8	German	56, 57
727	c.4088C>T (p.Ser1363Phe)	20	Missense	TMS8	Turkish	18, 62
728	c.4089-4090delTG	20	Deletion	TMS8	American	36

729	c.4091_4092del (p.Ser1365Cysfs*12)	20	Deletion	TMS8	British, North European, British, Finnish	4, 16, 40, 49, 50
730	c.4094delTG	20	Deletion	TMS8	British	49, 50
731	c.4094_4097delCTGT	20	Deletion	TMS8	Chinese	81
732	c.4094_4097del (p.Ser1365Trpfs*27)	20	Deletion	TMS8	Chinese-Han	17
733	c.4097_4098del (p.Val1366Glyfs*11)	20	Deletion	TMS8	Japanese	114
734	c.4103T>C (p.Leu1368Pro)	20	Missense	TMS8	Bulgarian	70
735	c.4106C>T (p.Ser1369Leu)	20	Missense	TMS8	Italian	15
736	c.4112T>C (p.Leu1371Pro)	20	Missense	TMS8	Chinese-Han, Thai	23, 34
737	c.4114C>T (p.Gln1372*)	20	Nonsense	TMS8	Chinese-Han, Thai	17, 34
738	c.4118T>C (p.Leu1373Pro)	20	Missense	TMS8	Japanese	97
739	c.4118T>G (p.Leu1373Arg)	20	Missense	TMS8	Brazilian	8
740	c.4124G>C (p.Cys1375Ser)	20	Missense	TMS8	Ukranian	38
741	IVS20+5 G>A	20	Splice site	TMS8	Chinese	32
742	IVS20+6T>C	20	Splice site	TMS8	Egyptian	84
743	IVS20-2A>G	20	Splice site	TMS8	Danish	72
744	c.4125-1G>A	21	Splice site	After TMS8	Greek	41
745	c.4135C>T (p.Pro1379Ser)	21	Missense	After TMS8	Italian	38
746	c.4153dupG (p.Glu1385Glyfs*24)	21	Duplication	After TMS8	Chinese	63
747	c.4162delG (p.Ala1388Argfs*5)	21	Deletion	After TMS8	Chinese	7
748	p.Met1392Lys	21	Missense	After TMS8	Taiwanese	2
749	c.4195delC (p.Gln1399Argfs*6)	21	Deletion	After TMS8	Saudi Arabian	157, 158, 159
750	c.4196A>G (p.Gln1399Arg)	21	Missense	After TMS8	Saudi Arabian	153
751	c.4230G>A (p.Trp1410*)	21	Nonsense	After TMS8	Egyptian	25
752	c.4272T>G (p.Tyr1424*)	21	Nonsense	After TMS8	Chinese	7
753	c.4301C>T (p.Thr1434Met)	21	Missense	C Terminus	Italian, Egyptian	18, 25, 62
754	c.4310dupA (p.Pro1438Alafs*11)	21	Duplication	C Terminus	Indian	71,125
755	c.4311insA (p.Lys1437Lys)	21	Insertion	C Terminus	Indian	71
756	p.Ala1445Pro	21	Missense	C Terminus	Taiwanese	2
757	c.4374_4375del (p.Arg1459Glyfs*2)	21	Deletion	C Terminus	Serbian	15
758	c.4396T>G (p.Stop1466ArgextX2)	21	Extension	C Terminus	Greek, Sardinian, Greek, Greek, Cypriot	1, 41, 47, 48

References

1. Loudianos G, Dessi V, Lovicu M, Angius A, Nurchi A, Sturniolo GC et al. Further delineation of the molecular pathology of Wilson disease in the Mediterranean population. Hum. Mutat. 1998;12: 89-94.
2. Wan L, Tsai CH, Hsu CM, Huang CC, Yang CC, Liao CC et al. Mutation analysis and characterization of alternative splice variants of the Wilson disease gene *ATP7B*. Hepatology. 2010;52:1662-1670.
3. Nanji MS, Nguyen VT, Kawasoe JH, Inui K, Endo F, Nakajima T et al. Haplotype and mutation analysis in Japanese patients with Wilson disease. Am. J. Hum. Genet. 1997;60:1423-1429.

4. Shah AB, Chernov I, Zhang HT, Ross BM, Das K, Lutsenko S et al. Identification and analysis of mutations in the Wilson disease gene (*ATP7B*): population frequencies, genotype-phenotype correlation, and functional analyses. Am. J. Hum. Genet. 1997;61: 317-328.
5. Wu ZY, Wang N, Lin MT, Fang L, Murong SX, Yu L. Mutation analysis and the correlation between genotype and phenotype of Arg778Leu mutation in chinese patients with Wilson disease. Arch. Neurol. 2001;58: 971-976.
6. Vrabelova S, Letocha O, Borsky M, Kozak L. Mutation analysis of the *ATP7B* gene and genotype/phenotype correlation in 227 patients with Wilson disease. Mol. Genet. Metab. 2005;86: 277-285.
7. Dong Y, Ni W, Chen WJ, Wan B, Zhao GX, Shi ZQ. Spectrum and Classification of *ATP7B* variants in a large cohort of Chinese patients with Wilson's disease guides genetic diagnosis. Theranostics. 2016;6: 638-649.
8. Deguti MM, Genschel J, Cancado EL, Barbosa ER, Bochow B, Mucenic M et al. Wilson disease: novel mutations in the *ATP7B* gene and clinical correlation in Brazilian patients. Hum. Mutat. 2004;23: 398.
9. Hua R, Hua F, Jiao Y, Pan Y, Yang X, Peng S et al. Mutational analysis of *ATP7B* in Chinese Wilson disease patients. Am. J. Transl. Res. 2016;8: 2851-2861.
10. Li K, Zhang WM, Lin S, Wen L, Wang ZF, Xie D et al. Mutational analysis of *ATP7B* in north Chinese patients with Wilson disease. J. Hum. Genet. 2013;58: 67-72.
11. Gupta A, Aikath D, Neogi R, Datta S, Basu K, Maity B et al. Molecular pathogenesis of Wilson disease: haplotype analysis, detection of prevalent mutations and genotype-phenotype correlation in Indian patients. Hum. Genet. 2005;118: 49-57.
12. Ray K, Gupta A. Gene symbol: *ATP7B*. Disease: Wilson's disease. Hum. Genet. 2005;118: 541.
13. Mukherjee S, Dutta S, Majumdar S, Biswas T, Jaiswal P, Sengupta M et al. Genetic defects in Indian Wilson disease patients and genotype-phenotype correlation. Parkinsonism. Relat. Disord. 2014; 20: 75-81.
14. Figus A, Angius A, Loudianos G, Bertini C, Dessi V, Loi A et al. Molecular pathology and haplotype analysis of Wilson disease in Mediterranean populations. Am. J. Hum. Genet. 1995;57: 1318-1324.
15. Lepori MB, Lovicu M, Dessi V, Zappu A, Incollu S, Zancan L et al. Twenty-four novel mutations in Wilson disease patients of predominantly Italian origin. Genet. Test. 2007;11: 328-332.
16. Curtis D, Durkie M, Balac (Morris) P, Sheard D, Goodeve A, Peake I et al. A study of Wilson disease mutations in Britain. Hum. Mutat. 1999;14: 304-311.
17. Gu YH, Kodama H, Du SL, Gu QJ, Sun HJ, Ushijima H. Mutation spectrum and polymorphisms in *ATP7B* identified on direct sequencing of all exons in Chinese Han and Hui ethnic patients with Wilson's disease. Clin. Genet. 2003;64: 479-484.
18. Loudianos G, Dessi V, Lovicu M, Angius A, Altuntas B, Giacchino R et al. Mutation analysis in patients of Mediterranean descent with Wilson disease: identification of 19 novel mutations. J. Med. Genet. 1999;36: 833-836.
19. Loudianos G, Dessi V, Angius A, Lovicu M, Loi A, Deiana M et al. Wilson disease mutations associated with uncommon haplotypes in Mediterranean patients. Hum. Genet. 1996;98: 640-642.
20. Ye S, Gong L, Shui QX, Zhou LF. Wilson disease: identification of two novel mutations and clinical correlation in Eastern Chinese patients. World. J. Gastroenterol. 2007;13: 5147-5150.
21. Genschel J, Sommer G, Haas R, Buettner C, Bochow B, Manns M et al. Three novel mutations, c314C>A, C778insC, and c1285+2T>A, in exon 2 of the Wilson disease gene. Hum. Mutat. 2000;16: 1023-1033.
22. Lam CW, Mak CM. Allele dropout in PCR-based diagnosis of Wilson disease: mechanisms and solutions. Clin. Chem. 2006;52: 517-520.
23. Mak CM, Lam CW, Tam S, Lai CL, Chan LY, Fan ST et al. Mutational analysis of 65 Wilson disease patients in Hong Kong Chinese: identification of 17 novel mutations and its genetic heterogeneity. J. Hum. Genet. 2008;53: 55-63.
24. Gu S, Yang H, Qi Y, Deng X, Zhang L, Guo Y et al. Novel ATPase Cu2+ transporting beta polypeptide mutations in Chinese families with Wilson's disease. PLoS One. 2013;8: e66526.
25. Abdelghaffar TY, Elsayed SM, Elsobky E, Bochow B, Buttner J, Schmidt H. Mutational analysis of *ATP7B* gene in Egyptian children with Wilson disease: 12 novel mutations. J. Hum. Genet. 2008;53: 681-687.
26. Margarit E, Bach V, Go´mez D,Bruguera M, Jara P, Queralt R et al. Mutation analysis of Wilson disease in the Spanish population -identification of a prevalent substitution and eight novel mutations in the *ATP7B* gene. Clin. Genet. 2005;68: 61-68.
27. Loudianos G, Kostic V, Solinas P, Lovicu M, Dessi V, Svetel M et al. Characterization of the molecular defect in the *ATP7B* gene in Wilson disease patients from Yugoslavia. Genet. Test. 2003;7: 107-112.
28. Aggarwal A, Chandhok G, Todorov T, Parekh S, Tilve S, Zibert A et al. Wilson disease mutation pattern with genotype-phenotype correlations from Western India: confirmation of p.C271* as a common Indian mutation and identification of 14 novel mutations. Ann. Hum. Genet. 2013;77: 299-307.
29. Dedoussis GV, Genschel J, Sialvera TE, Bochow B, Manolaki N, Manios Y et al. Wilson disease: high prevalence in a mountainous area of Crete. Ann. Hum. Genet. 2005;69: 268-274.
30. Cheng, N., submitted to http://www.wilsondisease.med.ualberta.ca/database.asp.
31. Okada T, Shiono Y, Hayashi H, Satoh H, Sawada T, Suzuki A et al. Mutational analysis of *ATP7B* and genotype-phenotype correlation in Japanese with Wilson's disease. Hum. Mutat. 2000;15: 454-462.
32. Li XH, Lu Y, Ling Y, Fu QC, Xu J, Zang GQ et al. Clinical and molecular characterization of Wilson's disease in China: identification of 14 novel mutations. BMC Med. Genet. 2011;12: 1-13.
33. Lepori MB, Zappu A, Incollu S, Dessi V, Mameli E, Demelia L et al. Mutation analysis of the A*TP7B* gene in a new group of Wilson's disease patients: contribution to diagnosis. Mol. Cell. Probes. 2012;26: 147-150.
34. Panichareon B, Taweechue K, Thongnoppakhun W, Aksornworanart M, Pithukpakorn M, Yenchitsomanus PT et al. Six novel *ATP7B* mutations in Thai patients with Wilson disease. Eur. J. Med. Genet. 2011;54: 103-107.

35. Tsai CH, Tsai FJ, Wu JY, Chang JG, Lee CC, Lin SP et al. Mutation analysis of Wilson disease in Taiwan and description of six new mutations. Hum. Mutat. 1998;12: 370-376.
36. Kuppala D, Deng J, Brewer GJ, Wang MW, Borjigin J. Wilson disease mutations in the American population: identification of five novel mutations in *ATP7B*. The Open Hepatology Journal. 2009;1: 1-4.
37. Wildenauer, D. personal communication.
38. Cox DW, Prat L, Walshe JM, Heathcote J, Gaffney D. Twenty-four novel mutations in Wilson disease patients of predominantly European ancestry. Hum. Mutat. 2005;26: 1-7.
39. Davies LP, Macintyre G, Cox DW. New mutations in the Wilson disease gene, *ATP7B*: implications for molecular testing. Genet. Test. 2008;12: 139-145.
40. Chappuis P, Callebert J, Quignon V, Woimant F, Laplanche JL. Late neurological presentations of Wilson disease patients in French population and identification of 8 novel mutations in the *ATP7B* gene. J. Trace. Elem. Med. Biol. 2007;21: 37-42.
41. Loudianos G, Lovicu M, Solinas P, Kanavakis E, Tzetis M, Manolaki N et al. Delineation of the spectrum of Wilson disease mutations in the Greek population and the identification of six novel mutations. Genet. Test. 2000;4: 399-402.
42. Haas R, Gutierrez-Rivero B, Knoche J, Boker K, Manns MP, Schmidt HH. Mutation analysis in patients with Wilson disease: identification of 4 novel mutations. Mutation in brief no. 250. Hum. Mutat. 1999;14: 1-8.
43. Duc HH, Hefter H, Stremmel W, Castaneda-Guillot C, Hernandez Hernandez A, Cox DW et al. His1069Gln and six novel Wilson disease mutations: analysis of relevance for early diagnosis and phenotype. Eur. J. Hum. Genet. 1998;6: 616-623.
44. Santhosh S, Shaji RV, Eapen CE, Jayanthi V, Malathi S, Chandy M et al. *ATP7B* mutations in families in a predominantly Southern Indian cohort of Wilson's disease patients. Indian. J. Gastroenterol. 2006;25: 277-282.
45. Asifa M, submitted to http://www.wilsondisease.med.ualberta.ca/database.asp.
46. Kalinsky H, Funes A, Zeldin A, Pel-Or Y, Korostishevsky M, Gershoni-Baruch R et al. Novel *ATP7B* mutations causing Wilson disease in several Israeli ethnic groups. Hum. Mutat. 1998;11: 145-151.
47. Loudianos G, Dessi V, Lovicu M, Angius A, Kanavakis E, Tzetis M et al. Haplotype and mutation analysis in Greek patients with Wilson disease. Eur. J. Hum. Genet. 1998;6: 487-491.
48. Butler P, McIntyre N, Mistry PK. Molecular diagnosis of Wilson disease. Mol. Genet. Metab. 2001;72: 223-230.
49. Thomas GR, Forbes JR, Roberts EA, Walshe JM, Cox DW. The Wilson disease gene: spectrum of mutations and their consequences. Nat. Genet. 1995;9: 210-217.
50. Thomas GR, Roberts EA, Walshe JM, Cox DW. Haplotypes and mutations in Wilson disease. Am. J. Hum. Genet. 1995;56: 1315-1319.
51. Waldenstrom E, Lagerkvist A, Dahlman T, Westermark K, Landegren U. Efficient detection of mutations in Wilson disease by manifold sequencing. Genomics. 1996;37: 303-309.
52. Wei Z, Huang Y, Liu A, Diao S, Yu Q, Peng Z et al. Mutational characterization of *ATP7B* gene in 103 Wilson's disease patients from Southern China: identification of three novel mutations. Neuroreport. 2014;25: 1075-1080.
53. Gromadzka G, Schmidt HH, Genschel J, Bochow B, Rodo M, Tarnacka B et al. Frameshift and nonsense mutations in the gene for ATPase 7B are associated with severe impairment of copper metabolism and with an early clinical manifestation of Wilson's disease. Clin. Genet. 2005;68: 524-534.
54. Prat Davies LM, Bugbee D. submitted to http://www.wilsondisease.med.ualberta.ca/database.asp.
55. Park S, Park JY, Kim GH, Choi JH, Kim KM, Kim JB et al. Identification of novel *ATP7B* gene mutations and their functional roles in Korean patients with Wilson disease. Hum. Mutat. 2007;28: 1108-1113.
56. Caca K, Ferenci P, Kuhn HJ, Polli C, Willgerodt H, Kunath B et al. High prevalence of the H1069Q mutation in East German patients with Wilson disease: rapid detection of mutations by limited sequencing and phenotype-genotype analysis. J. Hepatol. 2001;35: 575-581.
57. Huster D, Hoppert M, Lutsenko S, Zinke J, Lehmann C, Mossner J et al. Defective cellular localization of mutant ATP7B in Wilson's disease patients and hepatoma cell lines. Gastroenterol. 2003;124: 335-345.
58. Liu Y, Zhou H, Guo H, Bai Y. Genetic and clinical analysis in a cohort of patients with Wilson's disease in Southwestern China. Arch. Med. Res. 2015;46: 164-169.
59. Liu XQ, Zhang YF, Liu TT, Hsiao KJ, Zhang JM, Gu XF et al. Correlation of *ATP7B* genotype with phenotype in Chinese patients with Wilson disease. World. J. Gastroenterol. 2004;10: 590-593.
60. de Bie P, Muller P, Wijmenga C, Klomp LW. Molecular pathogenesis of Wilson and Menkes disease: correlation of mutations with molecular defects and disease phenotypes. J. Med. Genet. 2007; 44: 673-688.
61. Hamza I, Schaefer M, Klomp LW, Gitlin JD. Interaction of the copper chaperone HAH1 with the Wilson disease protein is essential for copper homeostasis. Proc. Natl. Acad. Sci. U S A. 1999;96: 13363-13368.
62. Loudianos G, Dessi V, Lovicu M, Angius A, Pirastu M, Cao A. Haplotype and mutation analysis in Mediterranean patients with Wilson disease. J. Trace. Elem. Exp. Med. 1999;12: 315-319.
63. Yang J, Chan P. Gene symbol: *ATP7B*. Disease: Wilson's disease. Hum. Genet. 2005;118: 539.
64. Kroll CA, Ferber MJ, Dawson BD, Jacobson RM, Mensink KA, Lorey F et al. Retrospective determination of ceruloplasmin in newborn screening blood spots of patients with Wilson disease. Mol. Genet. Metab. 2006;89: 134-138.
65. Hofer H, Willheim-Polli C, Knoflach P, Gabriel C, Vogel W, Trauner M et al. Identification of a novel Wilson disease gene mutation frequent in upper Austria: a genetic and clinical study. J. Hum. Genet. 2012;57: 564-567..
66. Paptheodorou, A., submitted to http://www.wilsondisease.med.ualberta.ca/database.asp.
67. Kemppainen R, Palatsi R, Kallioinen M, Oikarinen A. A homozygous nonsense mutation and a combination of two mutations of the Wilson disease gene in patients with different lysyl oxidase activities in cultured fibroblasts. J. Invest. Dermatol. 1997;108: 35-39.
68. Fan Y, Yu L, Han Y, Ren M, Yang R, Zhao S. Identification of three novel insertion/deletion mutations in Wilson disease's gene. Biochem. Genet. 2004;42: 377-384.
69. Loudianos G, Lovicu M, Dessi V, Tzetis M, Kanavakis E, Zancan L et al. Abnormal mRNA splicing resulting from consensus sequence splicing mutations of *ATP7B. Hum. Mutat.* 2002;20: 260-266.
70. Todorov, T. *et al*. Spectrum of mutations in the Wilson disease gene (*ATP7B*) in the Bulgarian population. *Clin. Genet*. 68, 474-476 (2005).
71. Kumar S, Thapa BR, Kaur G, Prasad R. Identification and molecular characterization of 18 novel mutations in the *ATP7B* gene from Indian Wilson disease patients: genotype. Clin. Genet. 2005;67: 443-445.
72. Moller LB, Horn N, Jeppesen TD, Vissing J, Wibrand F, Jennum P et al. Clinical presentation and mutations in Danish patients with Wilson disease. Eur. J. Hum. Genet. 2011;19: 935-941.

73. Todorov T., submited to http://www.wilsondisease.med.ualberta.ca/database.asp.
74. Shimizu N, Kawase C, Nakazono H, Hemmi H, Shimatake H, Aoki T. A novel RNA splicing mutation in Japanese patients with Wilson disease. Biochem. Biophys. Res. Commun. 1995;217: 16-20.
75. Gupta A, Chattopadhyay I, Dey S, Nasipuri P, Das SK, Gangopadhyay PK et al. Molecular pathogenesis of Wilson disease among Indians: a perspective on mutation spectrum in *ATP7B* gene, prevalent defects, clinical heterogeneity and implication towards diagnosis. Cell. Mol. Neurobiol. 2007;27: 1023-1033.
76. Gupta A, Maulik M, Nasipuri P, Chattopadhyay I, Das SK, Gangopadhay PK et al. Molecular diagnosis of Wilson disease using prevalent mutations and informative single-nucleotide polymorphism markers. Clin. Chem. 2007;53: 1601-1608.
77. Kim EK, Yoo OJ, Song KY, Yoo HW, Choi SY, Cho SW et al. Identification of three novel mutations and a high frequency of the Arg778Leu mutation in Korean patients with Wilson disease. Hum. Mutat. 1998;11: 275-278.
78. Wang, L. H. *et al*. Mutation analysis of 73 southern Chinese Wilson's disease patients: identification of 10 novel mutations and its clinical correlation. *J. Hum. Genet.* 56, 660-665 (2011).
79. Seidel J. Caca K, Schwab SG, Berr F, Wildenauer DB, Mentzel HJ et al. Disturbed copper transport in humans. Part 2: mutations of the *ATP7B* gene lead to Wilson disease (WD). Cell. Mol. Biol. 2001;47: 149-157.
80. Garcia-Villarreal L, Daniels S, Shaw SH, Cotton D, Galvin M, Geskes J et al. High prevalence of the very rare Wilson disease gene mutation Leu708Pro in the Island of Gran Canaria (Canary Islands, Spain): a genetic and clinical study. Hepatology. 2000;32: 1329-1336.
81. Zong Y, Kong X. Mutation analysis of 35 Wilson's disease pedigrees. Zhonghua. Yi. Xue. Yi. Chuan. Xue. Za. Zhi. 2016;33: 30-33.
82. Yoo HW. Identification of novel mutations and the three most common mutations in the human *ATP7B* gene of Korean patients with Wilson disease. Genet. Med. 2002;4: 43S-48S.
83. Folhoffer A, Ferenci P, Csak T, Horvath A, Hegedus D, Firneisz G et al. Novel mutations of the *ATP7B* gene among 109 Hungarian patients with Wilson's disease. Eur. J. Gastroenterol. Hepatol. 2007;19: 105-111.
84. Ghaffar TYA, Elsayed SM, Elnaghy S, Shadeed A, Elsobky ES, Schmidt H. Phenotypic and genetic characterization of a cohort of pediatric Wilson disease patients. BMC Pediatr. 2011;11: 56.
85. Yuan ZF. *et al*. Novel mutations of the *ATP7B* gene in Han Chinese families with pre-symptomatic Wilson's disease. *World. J. Pediatr*. 11, 255-260 (2015).
86. Zhu Y, Deng SY, Wan CM. A young boy with elevated aminotransferases in physical examination--Two novel missense mutations associated with Wilson's disease were found. Zhongguo. Dang. Dai. Er. Ke. Za. Zhi. 2015;17: 741-743.
87. Lovicu M, Lepori MB, Incollu S, Dessi V, Zappu A, Iorio R et al. RNA analysis of consensus sequence splicing mutations: implications for the diagnosis of Wilson disease. Genet. Test. Mol. Biomarkers. 2009;13: 185-191.
88. Zappu A, Magli O, Lepori MB, Dessi V, Diana S, Incollu S et al. High incidence and allelic homogeneity of Wilson disease in 2 isolated populations: a prerequisite for efficient disease prevention programs. J. Pediatr. Gastroenterol. Nutr. 2008;47: 334-338.
89. Firneisz G, Lakatos PL, Szalav F, Polli C, Glant TT, Ferenci P. Common mutations of *ATP7B* in Wilson disease patients from Hungary. Am. J. Med. Genet. 2002;108: 23-28.
90. Vrabelova S, Vanova P, Kopeckova L, Trunecka P. Molecular analysis of Wilson disease. Cas. Lek. Cesk. 2002;141: 642-645.
91. Al Jumah M, Majumdar R, Al Rajeh S, Awada A, Al Zaben, Traif A et al. A clinical and genetic study of 56 Saudi Wilson disease patients: identification of Saudi-specific mutations. Eur. J. Neurol. 2004;11: 121-124.
92. Caprai S, Loudianos G, Massei F, Gori L, Lovicu, Maggiore G. Direct diagnosis of Wilson disease by molecular genetics. J. Pediatr. 2006;148: 138-140.
93. Xu P, Liang X, Jankovic J, Le W. Identification of a high frequency of mutation at exon 8 of the *ATP7B* gene in a Chinese population with Wilson disease by fluorescent PCR. Arch. Neurol. 2001;58: 1879-1882.
94. Genschel J, Czlonkowska A, Sommer G, Buettner C, Bochow B, Lochs H et al. Three novel mutations (P760L, L1305P, Q1351Stop) causing Wilson disease. Hum. Mutat. 2001;17: 156.
95. Eapen CE, Santhosh S, Shaji RV, Chandy M, Chandy GM. Gene symbol: *ATP7B*. Disease: Wilson's disease. Hum. Genet. 2004;114: 606.
96. Forbes JR, Cox DW. Functional characterization of missense mutations in *ATP7B*: Wilson disease mutation or normal variant? Am. J. Hum. Genet. 1998;63: 1663-1674.
97. Bost M, Lachaux A, Accominotti M, Vandenberghe A. Mutation screening and genotype-phenotype correlation in 32 families with Wilson disease. J. Trace. Elem. Exp. Med. 1999;12: 321-329.
98. Forbes JR, Cox DW. Copper-dependent trafficking of Wilson disease mutant ATP7B proteins. Hum. Mol. Genet. 2000;9: 1927-1935.
99. Pendlebury ST, Rothwell PM, Dalton A, Burton EA. Strokelike presentation of Wilson disease with homozygosity for a novel T766R mutation. Neurology. 2004;63: 1982-1983.
100. Brage A, Tome S, García A, Carracedo A, Salas A. Clinical and molecular characterization of Wilson disease in Spanish patients. Hepatol. Res. 2007;37: 18-26.
101. Shimizu N, Nakazono H, Takeshita Y, Ikeda C, Fujii H, Watanabe A et al. Molecular analysis and diagnosis in Japanese patients with Wilson's disease. Pediatr. Int. 1999;41: 409-413.
102. Lee CC, Wu JY, Tsai FJ, Kodama H, Abe T, Yang CF et al. Molecular analysis of Wilson disease in Taiwan: identification of one novel mutation and evidence of haplotype-mutation association. J. Hum. Genet.2000;45: 275-279.
103. Mak CM, Tam S, Fan ST, Liu CL, Lam CW. Wilson's disease: a patient undiagnosed for 18 years. Hong. Kong. Med. J. 2006;12: 154-158.
104. Okada T, Morise T, Takeda Y, Mabuchi H. A new variant deletion of a copper-transporting P-type ATPase gene found in patients with Wilson's disease presenting with fulminant hepatic failure. J. Gastroenterol. 2000;35: 278-283.
105. Zali N, Mohebbi SR, Esteghamat S, Chiani M, Haghighi MM, Hosseini-Asl SMK et al. Prevalence of A*TP7B* gene mutations in Iranian patients with Wilson disease. Hepat. Mon. 2011;11: 890-894.
106. Chuang LM, Wu HP, Jang MH, Wang TR, Sue WC, Lin BJ et al. High frequency of two mutations in codon 778 in exon 8 of the *ATP7B* gene in Taiwanese families with Wilson disease. J. Med. Genet. 1996;33: 521-523.
107. Zhang DF, Teng JF. Direct sequencing of mutations in the copper-transporting P-type adenosine triphosphate (*ATP7B*) gene for diagnosis and pathogenesis of Wilson's disease. Genet. Mol. Res. 2016;15: 1-9.
108. Dastsooz H, Dehghani SM, Imanieh MH, Haghighat M, Moini M, Fardaei M. A new *ATP7B* gene mutation with severe condition in two unrelated Iranian families with Wilson disease. Gene. 2013;514: 48-53.
109. Manoochehri J, Dehshiri RM, Faraji H, Mohammadi S, Dastsooz H, Moradi T et al. Family screening for a novel *ATP7B* gene mutation, c.2335T>G, in the south of Iran. Iran. J. Ped. Hematol. Oncol. 2014;4: 26-31.

110. Simsek PO, Akman SA, Cakmur R, Terzioglu O. Mutation analysis of ATP7B gene in Turkish Wilson disease patients: identification of five novel mutations. Eur. J. Med. Genet. 2013;56: 175-179.
111. Papur, O.S., submitted to http://www.wilsondisease.med.ualberta.ca/database.asp
112. Owada M, Suzuki K, Fukushi M, Yamauchi K, Kitagawa T. Mass screening for Wilson's disease by measuring urinary holoceruloplasmin. J. Pediatr. 2002;140: 614-616.
113. Kok KF, Hoevenaars B, Waanders E, Drenth JP. Value of molecular analysis of Wilson's disease in the absence of tissue copper deposits: a novel *ATP7B* mutation in an adult patient. Neth. J. Med. 2008;66: 348-350.
114. Yamaguchi A, Matsuura A, Arashima S, Kikuchi Y, Kikuchi K. Mutations of *ATP7B* gene in Wilson disease in Japan: identification of nine mutations and lack of clear founder effect in a Japanese population. Hum. Mutat. 1998;11: S320-322.
115. Santhosh S, Shaji RV, Eapen CE, Jayanthi V, Malathi S, Finny P et al. Genotype phenotype correlation in Wilson's disease within families-a report on four south Indian families. World. J. Gastroenterol. 2008;14: 4672-4676.
116. Balakrishnan P, Kabra M, Arora NK, Kalra V. Gene symbol: *ATP7B*. Hum. Genet. 2007;120: 917.
117. Seto WK, Mak CM, But D, Hung L, Lam CW. Mutational analysis for Wilson's disease. Lancet. 2009;374; 662.
118. Lee JY, Kim YH, Kim TW, Oh SY, Kim DS, Shin BS. New novel mutation of the *ATP7B* gene in a family with Wilson disease. J. Neurol. Sci. 2012;313: 129-131.
119. Wan L, Tsai CH, Tsai Y, Hsu CM, Lee CC, Tsai FJ. Mutation analysis of Taiwanese Wilson disease patients. Biochem. Biophys. Res. Commun. 2006;345: 734-738..
120. Ye XN, Mao LP, Lou YJ, Tong HY. Hemolytic anemia as first presentation of Wilson's disease with uncommon *ATP7B* mutation. Int. J. Clin. Exp. Med. 2015;8: 4708-4711.
121. A-Tobi M, Kashoob M, Joshi S, Bayoumi R. A novel splice-site allelic variant is responsible for Wilson disease in an Omani family. Sultan. Qaboos. Univ. Med. J. 2000;11: 357-362.
122. Sundaresan S, Eapen CE, Shaji RV, Chandy M, Kurian G, Chandy G. Screening for mutations in *ATP7B* gene using conformation-sensitive gel electrophoresis in a family with Wilson's disease. Med. Sci. Monit. 2007;13: CS38-CS40.
123. Olsson C, Waldenstrom E, Westermark K, Landegre U, Syvanen AC. Determination of the frequencies of ten allelic variants of the Wilson disease gene (*ATP7B*), in pooled DNA samples. Eur. J. Hum. Genet. 2000;8: 933-938.
124. Chen L, Li X, Zheng Z, Lu X, Lin M, Pan C et al. A novel *ATP7B* gene mutation in a liver failure patient with normal ceruloplasmin and low serum alkaline phosphatase. Gene. 2014;538: 204-206.
125. Prasad R, Kaur G, Kumar S, Thapa BR. Two novel mutations (2976INSA, 4311insA) of *ATP7B* in a patient with Wilson's disease coexisting with glucose-6-phosphate dehydrogenase deficiency. J. Gastroenterol. Hepatol. 2005;20: 660-662.
126. Kumar S, Thapa BR, Kaur G, Prasad R. Familial gene analysis for Wilson disease from north-west Indian patients. Ann. Hum. Biol. 2006;33: 177-186.
127. Zhu HW, Tao ZB, Su G, Jin QY, Zhao LT, Zhu JR et al. Identification of two novel mutations in the *ATP7B* gene that cause Wilson's disease. World. J. Pediatr. 2017;13: 387-391.
128. Iidaa M, Teradaa K, Sambongic Y, Wakabayashic T, Miuraa N, Koyama K et al. Analysis of functional domains of Wilson disease protein (ATP7B) in Saccharomyces cerevisiae. FEBS. Lett.1998;428: 281-285.
129. Karunas AS, Mersiianova IV, Poliakov AV, Evgrafov OV, Khusnutdinova EK. Analysis of mutations and haplotypes of polymorphic markers in patients with Wilson-Konovalov disease from Bashkir. Genetika. 2000;36: 972-979.
130. Hsi G, Cullen LM, Macintyre G, Chen MM, Glerum DM, Cox DW. Sequence variation in the ATP-binding domain of the Wilson disease transporter, ATP7B, affects copper transport in a yeast model system. Hum. Mutat.2008;29: 491-501.
131. Morgan CT, Tsivkovskii R, Kosinsky YA, Efremov RG, Lutsenko S. The distinct functional properties of the nucleotide-binding domain of ATP7B, the human copper-transporting ATPase: analysis of the Wilson disease mutations E1064A, H1069Q, R1151H, and C1104F. J. Biol. Chem. 2004;279: 36363-36371.
132. Misrahi, M. submitted to http://www.wilsondisease.med.ualberta.ca/database.asp.
133. Tanzi RE, Petrukhin K, Chernov I, Pellequer JL, Wasco W, Ross B et al. The Wilson disease gene is a copper transporting ATPase with homology to the Menkes disease gene. Nat. Genet. 1993;5: 344-350..
134. Houwen RH, Juyn J, Hoogenraad TU, Ploos van Amstel JK, Berger R. H714Q mutation in Wilson disease is associated with late, neurological presentation. J. Med. Genet. 1995;32: 480-482.
135. Czlonkowska A, Rodo M, Gajda J, Ploos van Amstel HK, Juyn J, Houwen RH. Very high frequency of the His1069Gln mutation in Polish Wilson disease patients. J. Neurol. 1997;244: 591-592.
136. Maier-Dobersberger T, Ferenci P, Polli C, Balać P, Dienes HP, Kaserer K et al. Detection of the His1069Gln mutation in Wilson disease by rapid polymerase chain reaction. Ann. Intern. Med. 1997;127: 21-26.
137. Ivanova-Smolenskaya IA, Ovchinnikov IV, Karabanov AV, Deineko NL, Poleshchuk VV, Markova ED et al. The His1069Gln mutation in the *ATP7B* gene in Russian patients with Wilson disease. J. Med. Genet. 1999;36: 174.
138. Okkeri J, Bencomo E, Pietila M, Haltia T. Introducing Wilson disease mutations into the zinc-transporting P-type ATPase of Escherichia coli. The mutation P634L in the 'hinge' motif (GDGXNDXP) perturbs the formation of the E2P state. Eur. J. Biochem. 2002;269: 1579-1586.
139. Machado AAC, Deguti MM, Genschel J, Cançado ELR, Bochow B, Schmidt H et al. Neurological manifestations and *ATP7B* mutations in Wilson's disease. Parkinsonism. Relat. Disord. 2008;14: 246-249.
140. El-Mougy FA, Sharaf SA, Elsharkawy MM, Mandour IA, El-Essawy RA, Eldin AM et al. Gene mutations in Wilson disease in Egyptian children: report on two novel mutations. Arab. J. Gastroenterol. 2014;15: 114-118.
141. Guggilla SR, Senagari JR, Rao PN, Madireddi S. Spectrum of mutations in the ATP binding domain of ATP7B gene of Wilson Disease in a regional Indian cohort. Gene. 2015;569: 83-87.
142. Loudianos G, Dessi V, Lovicu M, Angius A, Figus A, Lilliu F et al. Molecular characterization of wilson disease in the Sardinian population-evidence of a founder effect. Hum. Mutat. 1999;14: 294-303.
143. Paradisi I, De Freitas L, Arias S. Most frequent mutation c.3402delC (p.Ala1135GlnfsX13) among Wilson disease patients in Venezuela has a wide distribution and two old origins. Eur. J. Med. Genet. 2015;58: 59-65.
144. Shiono Y, Wakusawa S, Hayashi H, Takikawa T, Yano M, Okada T et al. Iron accumulation in the liver of male patients with Wilson's disease. Am. J. Gastroenterol. 2001;96: 3147-3151.
145. Hayashi H, Wakusawa S, Yano M, Okada T. Genetic background of Japanese patients with adult-onset storage diseases in the liver. Hepatol. Res. 2007;37: 777-783.
146. Wu ZY, Zhao GX, Chen WJ, Wang N, Wan B, Lin MT et al. Genetic variation spectrum in *ATP7B* gene identified in Latvian patients with Wilson disease. Mol. Genet. Genomic. Med. 2005;4: 405-409.
147. Wu, Z. Y. et al. Mutation analysis of 218 Chinese patients with Wilson disease revealed no correlation between the canine copper toxicosis gene MURR1 and Wilson disease. J. Mol. Med. (Berl.) 84, 438-442 (2006).

148. Orru S, Thomas G, Loizedda A, Cox DW, Contu L. 24 bp deletion and Ala1278 to Val mutation of the *ATP7B* gene in a Sardinian family with Wilson disease. Hum. Mutat. 1997;10: 84-85.
149. Leggio L, Addolorato G, Loudianos G, Abenavoli L, Lepori MB, Vecchio FM et al. A new mutation of Wilson's disease P-type ATPase gene in a patient with cirrhosis and coombs-positive hemolytic anemia. Dig. Dis. Sci. 2006;51: 34-38.
150. Ohya K, Abo W, Tamaki H, Suawara C, Endo T, Nomachi S et al. Presymptomatic diagnosis of Wilson disease associated with a novel mutation of the *ATP7B* gene. Eur. J. Pediatr. 2002;161: 124-126.
151. Kusuda Y, Hamaguchi K, Mori T, Shin R, Seike M, Sakata T. Novel mutations of the *ATP7B* gene in Japanese patients with Wilson disease. J. Hum. Genet. 2000;45: 86-91.
152. Prella M, Baccala R, Horisberger JD, Belin D, Di Raimondo F, Invernizzi R et al. Haemolytic onset of Wilson disease in a patient with homozygous truncation of *ATP7B* at Arg1319. Br. J. Haematol. 2001;114: 23-232.
153. Al Jumah M, Majumdar R, Al Rajeh S, Awada A, Al Zaben A, Al Traif et al. A clinical and genetic study of 56 Saudi Wilson disease patients: identification of Saudi-specific mutations. Eur. J. Neurol. 2004;11: 121-124.
154. Majumdar R, Al-Jumah M, Zaidan R. A rare homozygous missense mutation in *ATP7B* exon 19 in a case of Wilson disease. Eur. Neurol. 2004;51: 52-54.
155. Moller LB, Ott P, Lund C, Horn N. Homozygosity for a gross partial gene deletion of the C-terminal end of *ATP7B* in a Wilson patient with hepatic and no neurological manifestations. Am. J. Med. Genet. A. 2005;138: 340-343.
156. Beyersdorff A, Findeisen A. Morbus Wilson: Case report of a two-year-old child as first manifestation. *Scand. J.* Gastroenterol. 2006;41: 496-497.
157. Majumdar R, Al Jumah M, Al Rajeh S, Fraser M, Al Zaben A, Awada A et al. A novel deletion mutation within the carboxyl terminus of the copper-transporting ATPase gene causes Wilson disease. J. Neurol. Sci. 2000;179: 140-143.
158. Hsi G, Cullen LM, Moira GD, Cox DW. Functional assessment of the carboxy-terminus of the Wilson disease copper-transporting ATPase, ATP7B. Genomics. 2004;83: 473-481.
159. Cater MA, La Fontaine S, Shield K, Deal Y, Mercer JF. ATP7B mediates vesicular sequestration of copper: insight into biliary copper excretion. Gastroenterology. 2006;130: 493-506.
